# Facile synthesis of oligoyne amphiphiles and their rotaxanes†
†Electronic supplementary information (ESI) available: Fig. S1–S4 and a comprehensive account of all experimental details, including synthetic procedures, analytical data, and NMR spectra of all novel compounds. See DOI: 10.1039/c4sc03154g



**DOI:** 10.1039/c4sc03154g

**Published:** 2014-10-24

**Authors:** Stephen Schrettl, Emmanuel Contal, Tobias N. Hoheisel, Martin Fritzsche, Sandor Balog, Ruth Szilluweit, Holger Frauenrath

**Affiliations:** a Ecole Polytechnique Fédérale de Lausanne (EPFL) , Institute of Materials , Laboratory of Macromolecular and Organic Materials , EPFL – STI – IMX – LMOM, MXG 037, Station 12 , 1015 Lausanne , Switzerland . Email: holger.frauenrath@epfl.ch; b ETH Zürich , Department of Materials , Vladimir-Prelog-Weg 1-5/10 , 8093 Zurich , Switzerland; c Adolphe Merklé Institute , Route de l'Ancienne Papeterie , CP209 , 1723 Marly , Switzerland

## Abstract

Carbon-rich organic compounds containing a series of conjugated triple bonds (oligoynes) are relevant synthetic targets, but an improved access to oligoynes bearing functional groups would be desirable. Here, we report the straightforward synthesis of two series of oligoyne amphiphiles with glycoside or carboxylate polar head groups, investigate their self-assembly behavior in aqueous media, and their use as precursors for the formation of oligoyne rotaxanes with cyclodextrin hosts. To this end, we employed mono-, di-, or triacetylenic building blocks that gave access to the corresponding zinc acetylides *in situ* and allowed for the efficient elongation of the oligoyne segment in few synthetic steps *via* a Negishi coupling protocol. Moreover, we show that the obtained oligoyne derivatives can be deprotected to yield the corresponding amphiphiles. Depending on their head groups, the supramolecular self-assembly of these amphiphiles gave rise to different types of carbon-rich colloidal aggregates in aqueous media. Furthermore, their amphiphilicity was exploited for the preparation of novel oligoyne cyclodextrin rotaxanes using simple host–guest chemistry in water.

## Abstract 2

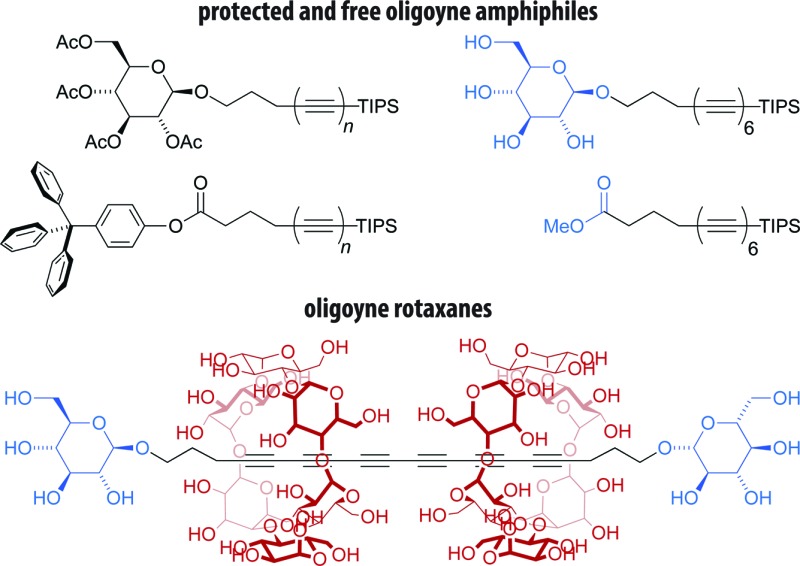
Carbon-rich organic compounds containing a series of conjugated triple bonds (oligoynes) are relevant synthetic targets, but an improved access to oligoynes bearing functional groups would be desirable.

## Introduction

Carbon-rich organic compounds containing a series of conjugated triple bonds (oligoynes) have attracted considerable interest because they were found in a variety of natural products,^1,2^ are under consideration as molecular wires,^3,4^ show intriguing non-linear optical properties,^5,6^ and have recently been used as molecular precursors for the preparation of carbon nanomaterials at room temperature.^7–9^ Moreover, homologous series of oligoynes have been investigated spectroscopically in an oligomer approach towards the properties of the elusive carbon allotrope carbyne (C≡C)_∞_.^10–15^ Terminal substituents with a high steric demand have been employed as protecting groups that hinder the metastable^16,17^ conjugated triple bonds from coming into close contact. In this way, derivatives of ever increasing length have been prepared. Notable examples include the investigations by Bohlmann,^18^ Jones,^19^ Walton,^20,21^ Gladysz,^12,22^ Hirsch,^10,11,23^ Cox,^17^ and, in particular, Tykwinski^5,14^ who recently introduced the supertrityl protecting group that provided sufficient steric hindrance to stabilize oligoynes with up to 22 conjugated C≡C bonds. Since the oligoynes in these examples did not carry any labile functional groups, harsh reagents and conditions were typically employed in their preparation, such as the Fritsch–Buttenberg–Wiechell rearrangement.^24–28^ In order to gain access to oligoynes bearing additional chemical functional groups, significantly milder reaction conditions are required, and synthetic approaches have traditionally been based on transition-metal-catalyzed couplings such as the Cadiot–Chodkiewicz^11,29–34^ and Sonogashira^35–41^ reaction. The direct bond formation between sp-hybridized carbon atoms by these methods has been successfully applied in the synthesis of diynes.^1^ The preparation of unsymmetric higher oligoynes, however, is typically plagued by low conversions, selectivity issues, and side product formation, as alkynes and haloalkynes are prone to undergo oxidative, and reductive homocoupling reactions under these conditions. The Negishi reaction^42–44^ offers a viable alternative but has rarely been applied in the direct cross-coupling of sp-hybridized carbons.^45,46^ We recently reported a heterocoupling protocol based on the Negishi coupling that allowed us to prepare unsymmetric glycosylated oligoynes.^47^ The required zinc acetylide was prepared *in situ* from a stable trimethylsilyl derivative and coupled with a bromoacetylene to yield the desired heterocoupling products up to the tetrayne in high yields. When further extending our approach to oligoynes beyond the tetrayne, however, we experienced issues of product stability that we now found to be associated to the use of a single methylene group as a spacer and the lability of the chosen trimethylsilyl protecting group at the oligoyne terminus.

**Fig. 1:**
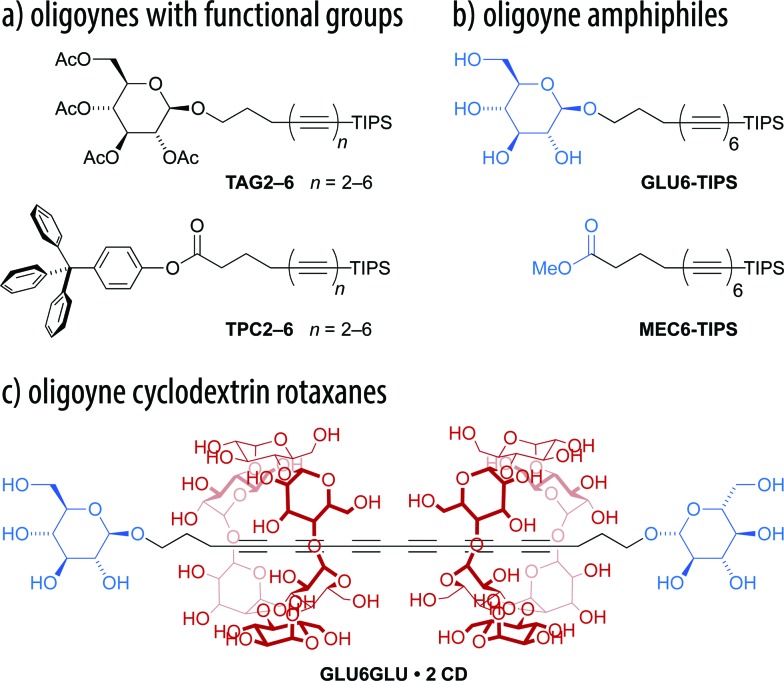
(a) Oligoyne derivatives with chemical functional groups such as the tetra-*O*-acetyl β-d-glucosides **TAG2–6** and the tritylphenyl carboxylates **TPC2–6** can be deprotected to obtain (b) the corresponding amphiphilic oligoyne derivatives **GLU6-TIPS** and **MEC6-TIPS**. These may either be used to prepare carbon-rich colloidal aggregates or (c) oligoyne cyclodextrin rotaxanes **GLU6GLU·2 CD**.

Here, we demonstrate the straightforward synthesis of two series of oligoynes bearing glycoside and carboxylate groups, the tetra-*O*-acetyl β-d-glucosides **TAG2–6** and the tritylphenyl carboxylates **TPC2–6** (Fig. 1). We were able to successfully prepare these compounds up to the hexayne derivatives on the multi-gram scale. To this end, we synthesized derivatives with an extended spacer length between the oligoynes and the functional group, and used a sterically more demanding TIPS protecting group at the oligoyne terminus. Moreover, we employed mono-, di-, or triacetylenic building blocks that could be conveniently prepared on a large scale, gave access to the corresponding zinc acetylides *in situ* and were compatible with the Negishi protocol. This allowed for the efficient and concise elongation of the oligoyne segment in few synthetic steps. We found that the obtained oligoyne derivatives could be deprotected to yield the corresponding amphiphiles, such as the hexayne glucoside **GLU6-TIPS** or the methyl carboxylate **MEC6-TIPS** (Fig. 1). These amphiphiles gave rise to carbon-rich colloidal aggregates by supramolecular self-assembly in aqueous media. Finally, we exploited their amphiphilicity for the preparation of the novel hexayne cyclodextrin rotaxane **GLU6GLU·2 CD** (Fig. 1) using simple host–guest chemistry in water. Hence, we have developed the tools to access and handle amphiphilic oligoyne derivatives with chemical functional groups, which may in turn be utilized for the further preparation of carbon-rich compounds or nanomaterials.

## Results and discussion

### Synthesis of oligoyne derivatives with functional groups

**Scheme 1:**
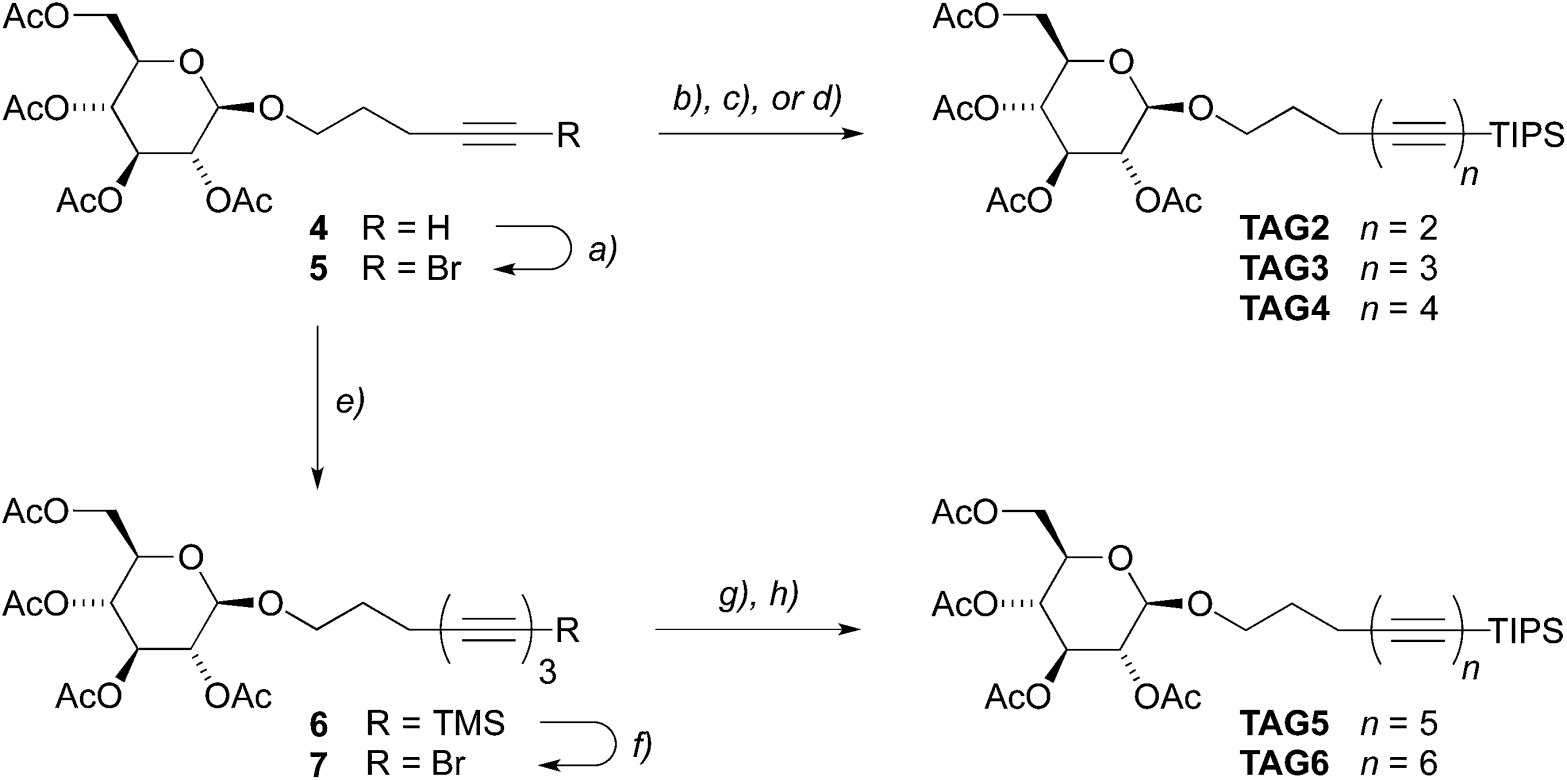
Synthesis of the glycosylated oligoynes **TAG2–6** up to the hexaynes. Reagents and conditions: (a) AgNO_3_, NBS, MeCN, 93%; (b) TIPS–C≡C–ZnCl **1**, PdCl_2_(dppf)·DCM, THF/toluene, 87%; (c) TIPS–(C≡C)_2_–ZnCl **2a**, PdCl_2_(dppf)·DCM, THF/toluene, 78%; (d) TIPS–(C≡C)_3_–ZnCl **3**, PdCl_2_(dppf)·DCM, THF/toluene, 51%; (e) TMS–(C≡C)_2_–ZnCl **2b**, PdCl_2_(dppf)·DCM, THF/toluene, 72%; (f) AgF, NBS, MeCN, 81%; (g) TIPS–(C≡C)_2_–ZnCl **2a**, PdCl_2_(dppf)·DCM, THF/toluene, 42%; (h) TIPS–(C≡C)_3_–ZnCl **3**, PdCl_2_(dppf)·DCM, THF/toluene, 55%.

**Scheme 2:**
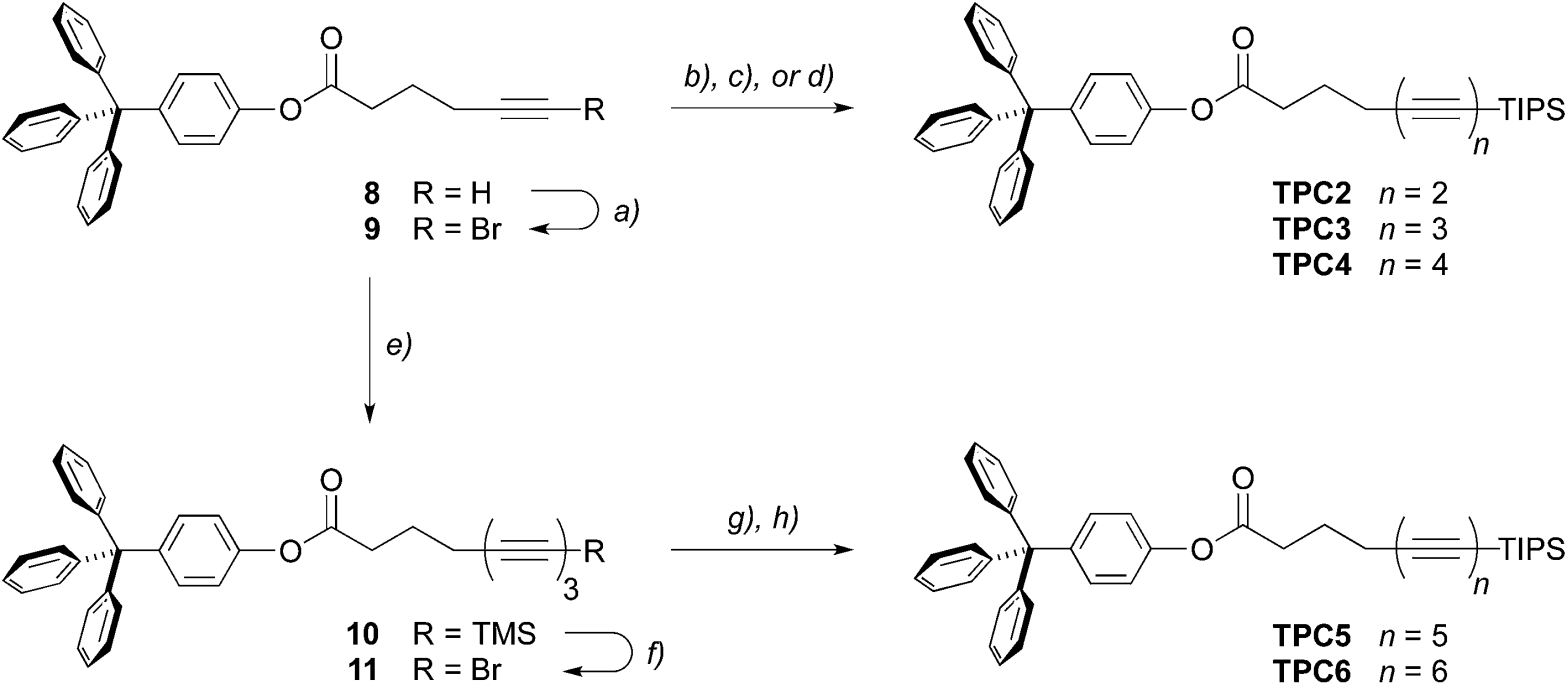
Synthesis of the oligoyne tritylphenyl carboxylates **TPC2–6** up to the hexaynes. Reagents and conditions: (a) AgNO_3_, NBS, DCM/MeCN, 96%; (b) TIPS–C≡C–ZnCl **1**, PdCl_2_(dppf)·DCM, THF/toluene, 68%; (c) TIPS–(C≡C)_2_–ZnCl **2a**, PdCl_2_(dppf)·DCM, THF/toluene, 51%; (d) TIPS–(C≡C)_3_–ZnCl **3**, PdCl_2_(dppf)·DCM, THF/toluene, 65%; (e) TMS–(C≡C)_2_–ZnCl **2b**, PdCl_2_(dppf)·DCM, THF/toluene, 71%; (f) AgF, NBS, DCM; (g) TIPS–(C≡C)_2_–ZnCl **2a**, PdCl_2_(dppf)·DCM, THF/toluene, 59% over two steps starting from **10**; (h) TIPS–(C≡C)_3_–ZnCl **3**, PdCl_2_(dppf)·DCM, THF/toluene, 44% over two steps starting from **10**.

The synthetic approach we have applied here for the preparation of oligoynes bearing chemical functional groups extends upon the facile coupling of bromoacetylenes with zinc acetylides similar to the Negishi coupling conditions developed previously in our laboratory.^47^ During scale-up of our previously reported approach, we noticed a significantly decreased yield for large (>10 g) batch sizes and oligoynes longer than tetraynes. For the present investigation, we therefore reduced the electronic effects of the substituents on the oligoyne moiety by increasing the spacer length, and used the sterically more demanding triisopropylsilyl (TIPS) group for the preparation of longer oligoynes. Moreover, we decided to utilize the silyl-protected oligoyne zinc acetylides R–(C≡C)_*m*_–ZnCl **1–3** (R = TIPS, TMS; *m* = 1–3) as building blocks in order to reduce the number of synthetic steps. To this end, the silylated precursors TIPS-C≡C–H and TIPS-(C≡C)_2_–H were deprotonated with *n*-BuLi followed by the addition of ZnCl_2_ in THF to furnish TIPS–C≡C–ZnCl **1** and TIPS–(C≡C)_2_–ZnCl **2a**. Similarly, the precursors TIPS–(C≡C)_2_–TMS,^48^ TMS–(C≡C)_2_–TMS,^49,50^ and TIPS–(C≡C)_3_–TMS^48^ were monolithiated by the selective removal of one TMS group using MeLi·LiBr.^51^ Subsequent transmetalation with ZnCl_2_ gave TIPS–(C≡C)_2_–ZnCl **2a**, TMS–(C≡C)_2_–ZnCl **2b**, as well as TIPS–(C≡C)_3_–ZnCl **3**, respectively. The resulting solutions of the zinc acetylides were then added to solutions containing the palladium catalyst and the bromoacetylenes.^39,40,52^ In this way, we were able to synthesize the series of diynes to tetraynes carrying the protected tetra-*O*-acetyl β-d-glucosides **TAG2–4** (Scheme 1), in only two steps from commercial precursors, and in isolated yields for the final coupling step of 87%, 78%, and 51% on a 1 g scale. In the same way, the corresponding tritylphenyl carboxylates **TPC2–4** (Scheme 2) were obtained in isolated yields of 68% (1 g scale), 51% (9 g scale), and 65% (1 g scale), respectively. The key to access the higher homologues was the efficient conversion of the bromoalkynes **5** or **9** to the corresponding TMS-protected triynes **6** and **10** (77% and 71% on a 10 g scale, respectively) by Negishi coupling reactions with TMS–(C≡C)_2_–ZnCl **2b**, and their subsequent desilylative bromination^39,40^ to the desired bromotriynes **7** and **11**. As both bromotriynes were found to rapidly degrade in the solid state,^53^ we prepared **7** directly before applying it to subsequent coupling steps, and **11** was even only generated *in situ*. The final Negishi coupling reactions with either the diyne **2a** or the triyne **3** then furnished the tetra-*O*-acetyl β-d-glucosyl pentaynes and hexaynes **TAG5–6** (42% and 55% on reaction scales of 1 g and 0.5 g, respectively) as well as the corresponding tritylphenyl carboxylates **TPC5–6** (59% and 44% over two steps on reaction scales of 0.3 g and 2 g, respectively). We furthermore increased the batch size for the preparation of **TAG6** to 6.5 g to test the scalability of the employed procedure and obtained the desired hexayne in 43% yield.

The developed synthetic pathway has, hence, provided convenient access to unsymmetric oligoynes up to the hexaynes with different functional groups on the preparative scale. The new acetylenic building blocks significantly facilitated the procedure, and the obtained products were found to be considerably more stable than the previously reported oligoynes with a methylene spacer and a TMS protecting group.^47^ No decomposition was observed in the neat state, except for the hexayne **TAG6** that slowly degraded at 4 °C in the bulk over a period of six weeks and was, therefore, stored in a dilute Et_2_O solution at –20 °C. Due to the expected general sensitivity of higher oligoynes a storage at temperatures of –20 °C is generally advisable to ensure the integrity of the compounds over the long term.

### Spectroscopic properties of the oligoyne derivatives

**Fig. 2:**
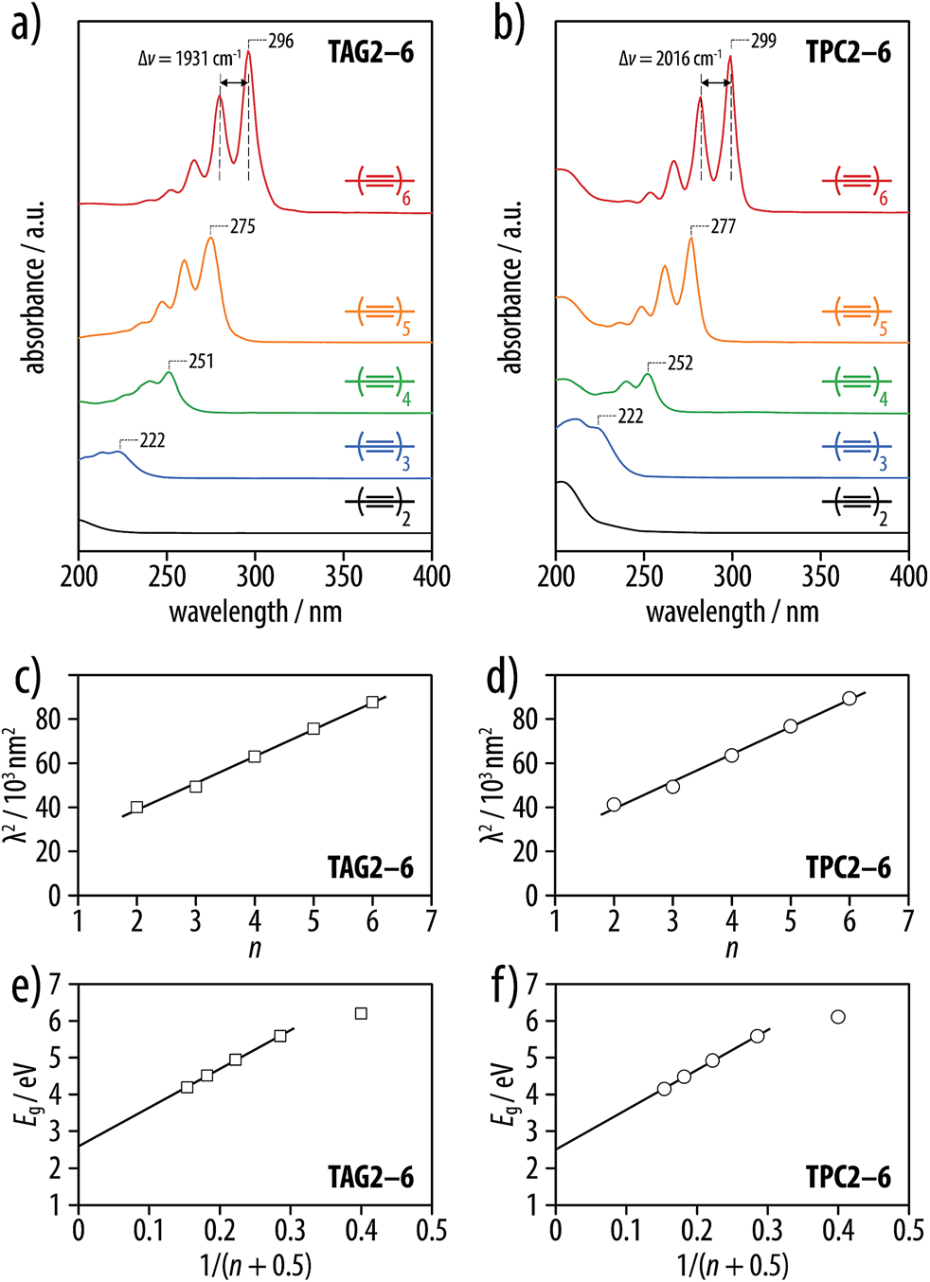
(a) UV-vis spectra of the tetra-*O*-acetyl β-d-glucosyl oligoynes **TAG2–6** in acetonitrile (concentrations in the range of *c* = 3.03–4.21 μmol L^–1^), and (b) of the corresponding tritylphenyl carboxylates **TPC2–6** in cyclohexane (concentrations in the range of *c* = 5.86–6.55 μmol L^–1^). (c and d) Lewis–Calvin plots of the UV-vis absorption maxima of the two series with linear regressions with slopes of *k* = 12.2 × 10^3^ and 12.4 × 10^3^ nm^2^ (*R* ^2^ = 0.996 and 0.991). (e and f) Plots of the energy of the optical transition *versus* 1/(*n* + 0.5), with the number of triple bonds *n*. An extrapolation of the linear regression to *n* = ∞ yields values of 480 nm (2.6 eV) and 498 nm (2.5 eV), respectively, for the saturation wavelength of an infinitely long chain of conjugated triple bonds (*R* ^2^ = 0.999 in both cases).

**Fig. 3:**
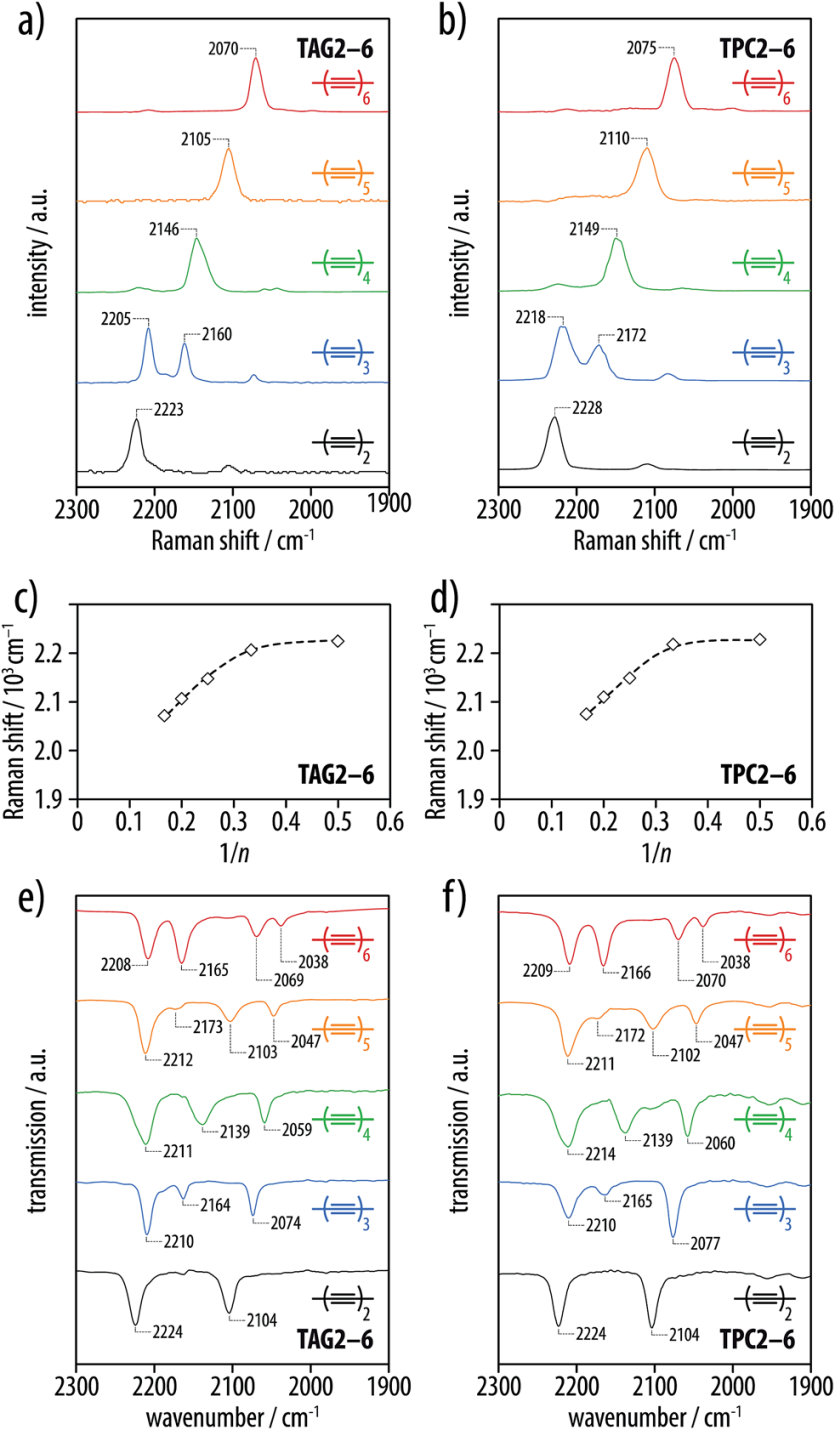
IR and Raman spectra for the series of tetra-*O*-acetyl β-d-glucosyl oligoynes **TAG2–6** and the corresponding tritylphenyl carboxylates **TPC2–6**. (a and b) Solid-state Raman spectra, (c and d) plots of the Raman band positions *versus* 1/*n* (the lines serve as guides to the eye), and (e and f) solid-state IR spectra of **TAG2–6** and **TPC2–6**.

**Fig. 4:**
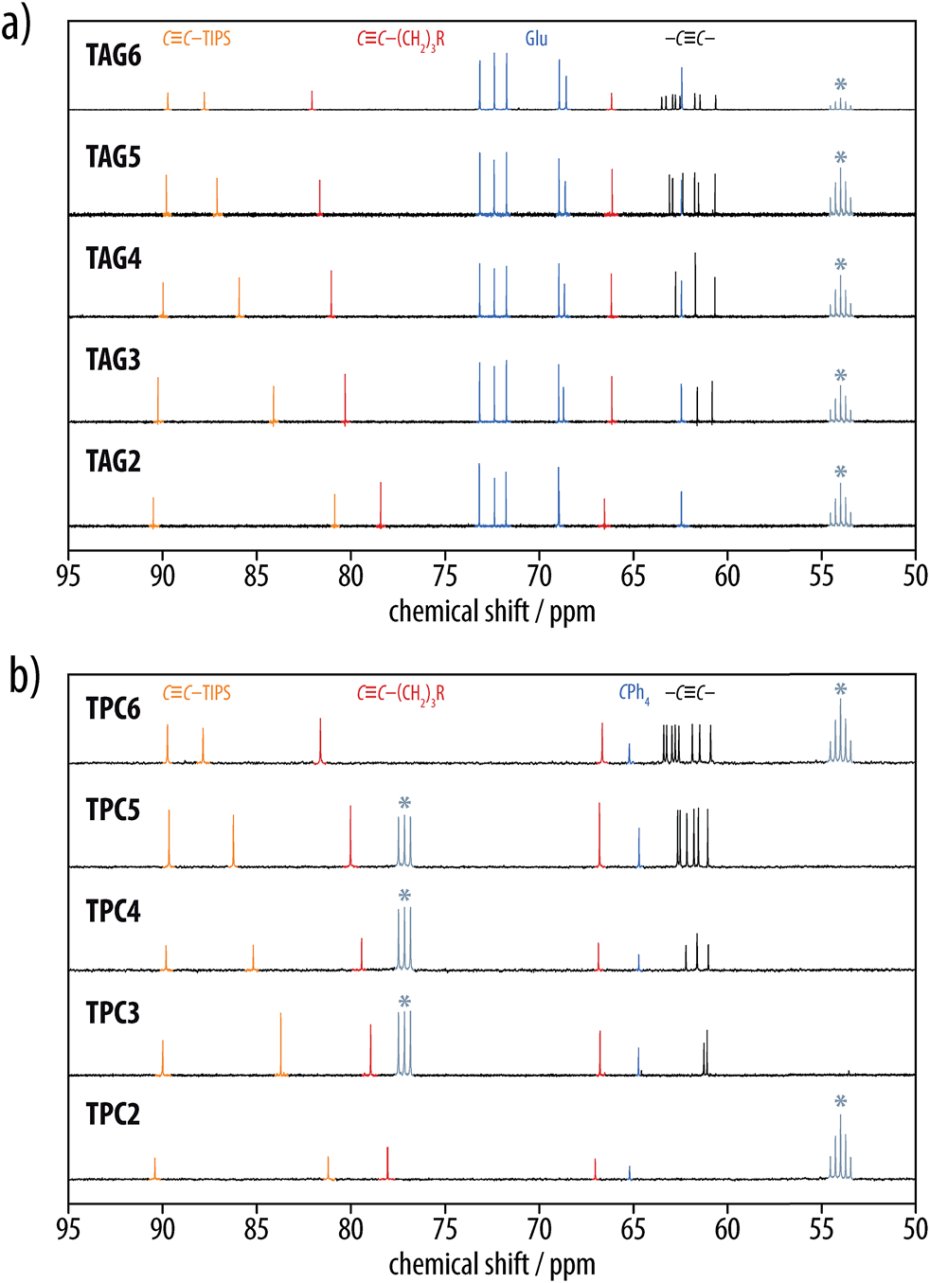
Acetylene region of the ^13^C NMR spectra of (a) the glucosylated oligoynes **TAG2–6** and (b) the corresponding tritylphenyl carboxylates **TPC2–6**. The resonances for the internal acetylene carbons converged to chemical shifts between 65–60 ppm (black), the acetylene carbons neighboring the TIPS group appeared above 80 ppm (orange), and the acetylene carbons next to the propylene spacer were observed at chemical shifts of 66 and 78–82 ppm (red), respectively (glucoside and tritylphenyl peaks highlighted in blue; * = denotes residual solvent signals from either CD_2_Cl_2_ or CDCl_3_).

The NMR, UV-vis, Raman, and IR spectra of the tetra-*O*-acetyl β-d-glucosyl oligoynes **TAG2–6** and the corresponding tritylphenyl carboxylates **TPC2–6** showed trends similar to what had previously been described for series of symmetric oligoynes.^5,11,13^ The UV-vis spectra for the respective members of the two series **TAG2–6** in acetonitrile and **TPC2–6** in cyclohexane were almost identical (Fig. 2a and b), and plots of the highest wavelength absorptions according to Lewis and Calvin^10,20^ (Fig. 2c and d) as well as extrapolations of the highest wavelength absorptions according to Wegner^54^ (Fig. 2e and f) gave results in excellent agreement with previously published values. The solid state Raman spectra of **TAG2–6** and **TPC2–6** (Fig. 3a and b) revealed bands at positions that matched reasonably well with the vibronic fine structure observed in the optical spectra. Consistent with previously reported data,^6,11^ the positions of the Raman bands shifted toward lower wavenumbers with an increasing number of triple bonds, as illustrated by the plots of the Raman shifts *versus* the inverse number of triple bonds 1/*n* (Fig. 3c and d). The solid state IR spectra of corresponding members of the **TAG2–6** and **TPC2–6** series exhibited the same peak patterns for the oligoyne vibrations in the range of 2250–2050 cm^–1^ (Fig. 3e and f). Finally, the ^13^C-NMR spectra of these series revealed resonances for the internal acetylene carbons converging to chemical shifts between 65 and 60 ppm (Fig. 4). In both series, the signals of the two acetylene carbons next to the silyl end groups appeared above 80 ppm, while the resonances of the two acetylene carbons neighboring the propylene spacer were observed at chemical shifts of 66 and 78–82 ppm, respectively.

### Preparation of carbon-rich amphiphiles

**Scheme 3:**
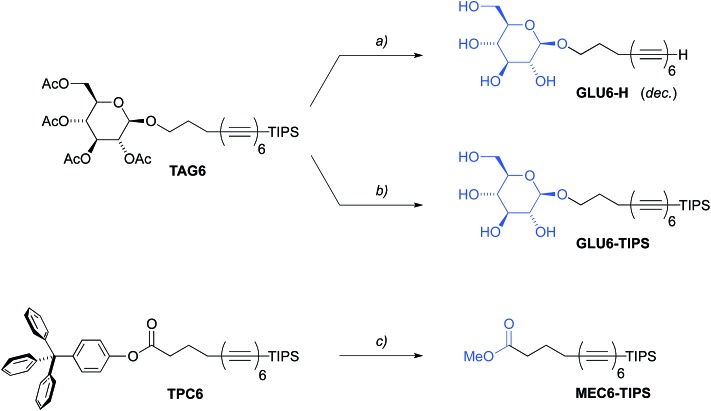
Synthesis of oligoyne amphiphiles. Reagents and conditions: (a) CsF, NaOMe, dioxane/MeOH, r.t., 2 h; (b) NaOMe, dioxane/MeOH, r.t., 2 h; then Amberlite (H^+^), 30 min; (c) NaOMe, DCM/MeOH, r.t., 5 h; then Amberlite (H^+^), 1 h. Conversions were quantitative in all cases, but the products were kept in solution and isolated yields not determined.

The obtained oligoynes serve as protected precursors of oligoyne amphiphiles that undergo supramolecular self-assembly into carbon-rich colloidal aggregates in aqueous dispersion and can be used for the formation of novel oligoynes rotaxanes with cyclodextrin hosts.^7,8^ We first investigated the complete deprotection of the tetra-*O*-acetyl β-d-glucosyl hexayne **TAG6**, that is, the deacetylation of the glucosyl head group by treatment with substoichiometric amounts of NaOMe in methanol according to Zemplén,^55^ and the simultaneous or subsequent desilylation with cesium fluoride (Scheme 3). Solutions of the desilylated compound **Glu6-H**, however, quickly turned dark brown, indicating the fast degradation of the hexayne moieties. While this highlighted the propensity of the deprotected hexaynes to possibly undergo carbonization after deprotection, the rapid process was difficult to control. We therefore decided to investigate the presumably less reactive derivatives with a deprotected hydrophilic head group but an intact terminal silyl group. To this end, the hexaynes **TAG6** and **TPC6** were both deprotected using NaOMe in MeOH-containing solutions, resulting in the glucosylated amphiphile **GLU6-TIPS** and the methyl carboxylate **MEC6-TIPS**, respectively (Scheme 3).

**Fig. 5:**
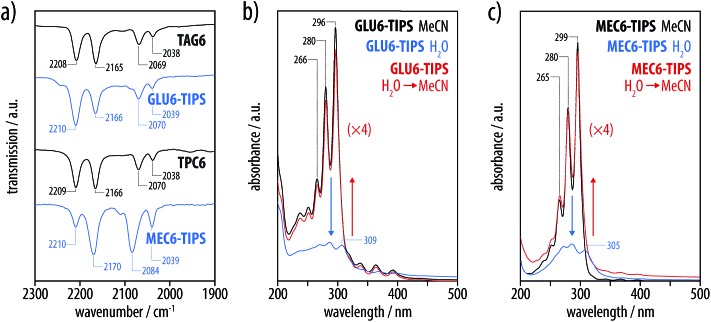
(a) Comparison of the solid-state IR spectra of the protected hexaynes **TAG6** and **TPC6** with the corresponding deprotected glucosyl hexaynes **GLU6-TIPS** and methyl carboxylates **MEC6-TIPS**. (b) Solution-phase UV-vis spectra of deprotected glucosyl hexayne **GLU6-TIPS** and (c) of the hexayne methyl carboxylates **MEC6-TIPS** in acetonitrile (black, *c* = 3.83 μmol L^–1^, *c* = 4.97 μmol L^–1^, respectively), in aqueous dispersion at the same concentration (blue, *c* = 3.83 μmol L^–1^, *c* = 4.97 μmol L^–1^, respectively), and upon diluting the latter with a three-fold excess of MeCN (red, *c* = 0.96 μmol L^–1^, *c* = 1.24 μmol L^–1^, respectively; shown four times magnified, for the sake of comparison).

**Fig. 6:**
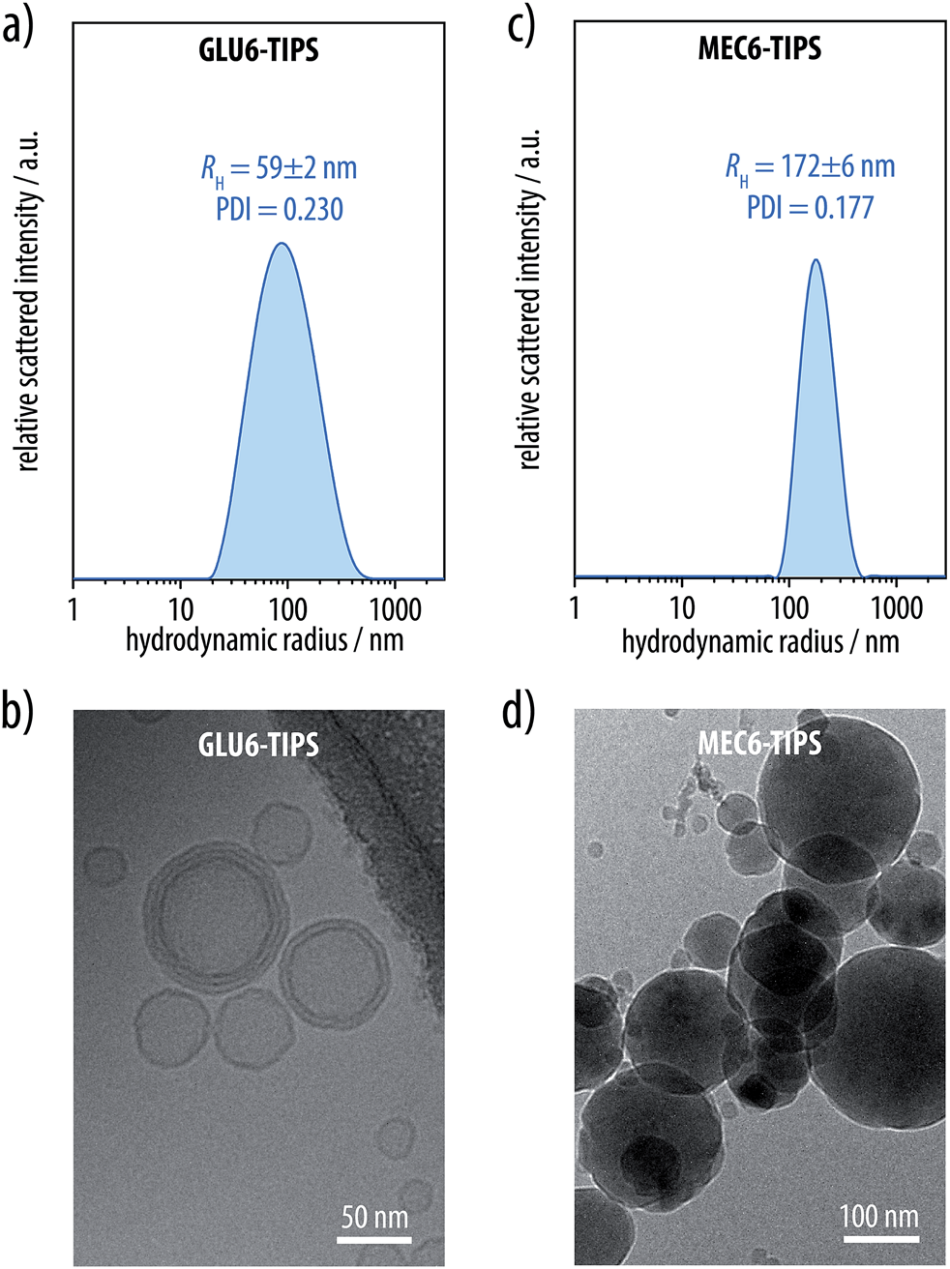
(a) Dynamic light scattering (DLS) size distributions by intensity of aqueous dispersions of the glucosylated hexayne **GLU6-TIPS** indicated the presence of colloidal aggregates with sizes of 59 (±2) nm (PDI 0.230). (b) Cryogenic transmission electron microscopy (cryo-TEM) images of aqueous dispersions of **GLU6-TIPS** revealed the presence of a mixture of unilamellar and multilamellar spherical vesicles with a broad variation of diameters. (c) DLS of the methyl carboxylate **MEC6-TIPS** in aqueous dispersion showed colloidal aggregates with a size of 172 (±6) nm (PDI 0.177). (d) Cryo-TEM images of these dispersions showed filled nanospheres that supposedly represent compact droplets of **MEC6-TIPS**.

Our investigations by IR and UV-vis spectroscopy (Fig. 5), first of all, confirmed that the hexayne segments remained intact in both cases. Moreover, UV-vis spectroscopy proved that both molecules underwent a reversible aggregation without premature degradation of the hexayne moiety in aqueous media. Thus, both the glucosylated hexayne **GLU6-TIPS** and the methyl carboxylate **MEC6-TIPS** showed the UV-vis absorption bands characteristic for molecularly disperse solutions in MeCN, which gave way to a broad and less intense signal with a poorly defined fine structure in aqueous dispersion (Fig. 5b and c). Diluting these dispersions with a three-fold excess of MeCN re-established the spectroscopic fine structure of the molecularly disperse species in both cases, proving the reversibility of the process. While the protected oligoyne derivatives **TAG6** and **TCP6** were completely insoluble in aqueous media, both the glucosylated hexayne **GLU6-TIPS** and the methyl carboxylate **MEC6-TIPS** readily formed opalescent colloidal dispersions in water, as corroborated by dynamic light scattering (DLS) experiments in combination with cryogenic transmission electronic microscopy (cryo-TEM) imaging. From DLS, we determined particle sizes of 59 (±2) nm (PDI 0.230) for **GLU6-TIPS** (Fig. 6a) and depolarized dynamic light scattering on the dispersions was practically zero, so that shape anisotropy can be excluded. According to representative cryogenic transmission electron microscopy (cryo-TEM) images (Fig. 6b), the dispersions contained a mixture of unilamellar and multilamellar spherical vesicles with a broad variation of diameters in the range of 20–120 nm. These vesicles exhibited a uniform wall thickness on the order of 4–5 nm, suggesting that the vesicle membranes were formed from bilayers of the molecules. By contrast, dispersions of **MEC6-TIPS** contained significantly larger spherical particles with a diameter of 172 (±6) nm (PDI 0.177) according to DLS (Fig. 6c) and depolarized DLS. Cryo-TEM images of these dispersion revealed what looked like filled nanospheres with a broad distribution of diameters in the range of 20–250 nm (Fig. 6d). Since the latter are up to two orders of magnitude larger than the extended molecular length of **MEC6-TIPS** of about 2.6 nm, one has to conclude that these nanospheres can not be micelles but represent compact droplets of **MEC6-TIPS**. This implies that the amphiphilicity of the methyl carboxylates was not sufficient to stabilize well-defined colloidal structures.

### Rotaxane formation with α-cyclodextrin in aqueous solution

Rotaxanes of π-conjugated oligomers and polymers have previously been investigated as potential “insulated molecular wires”,^56^ and cyclodextrins have frequently been used as hosts for small linear hydrophobic guests.^57–60^ Likewise, the stabilization of oligoynes by encapsulation *via* rotaxane formation has already been discussed,^56,61^ but only few examples are known to date.^62–64^ In contrast to these examples of oligoyne rotaxanes that relied on the coordination of the copper catalyst to the macrocyclic host,^63,64^ we demonstrate here that the amphiphilic nature of the deprotected oligoynes can be exploited for the straightforward preparation of oligoyne rotaxanes by simple host–guest chemistry with α-cyclodextrin in hydrophilic media.

**Scheme 4:**
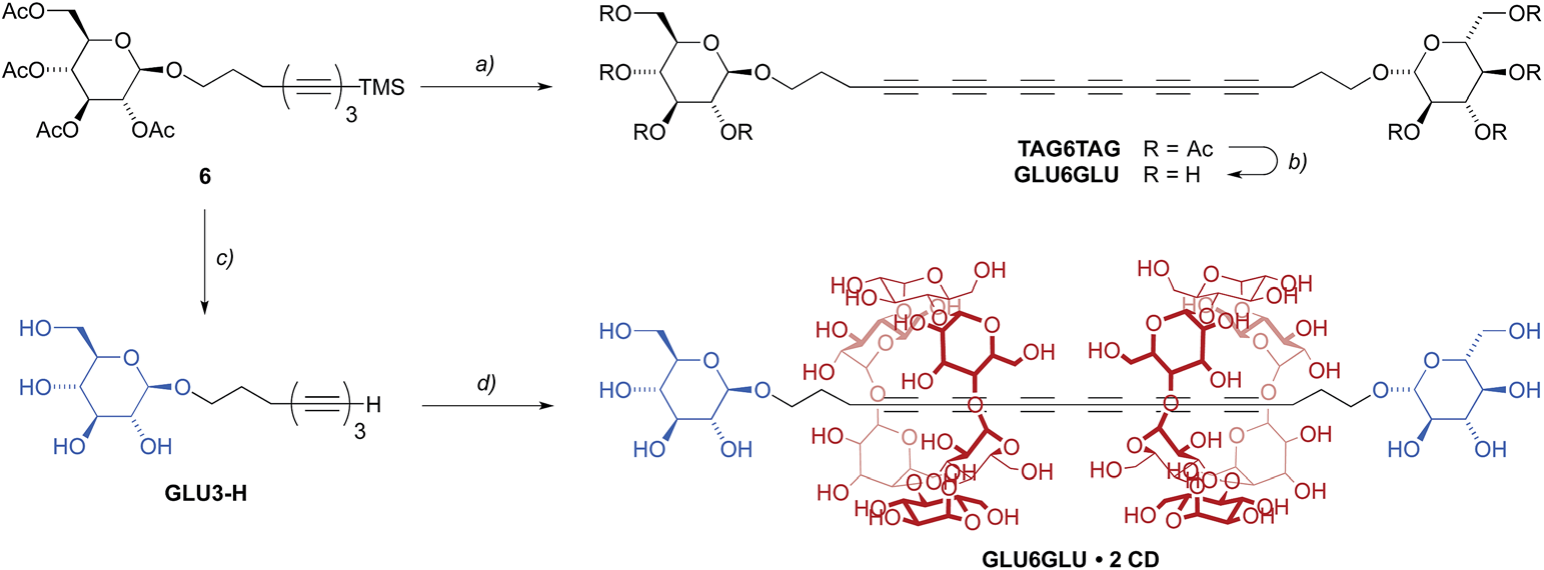
Synthesis of hexayne rotaxane **GLU6GLU·2 CD** and hexayne **GLU6GLU**. Reactions and conditions: (a) CsF, THF/H_2_O; then Cu(OAc)_2_, 2,6-lutidine, DCM, 73%; (b) NaOMe, 1,4-dioxane/MeOH (5 : 1), r.t., 3 h (yield not determined); (c) NaOMe, Et_2_O/MeOH (4 : 1), r.t., 2 h; then Amberlite (H^+^) (product not isolated); (d) α-cyclodextrin (0.1 M in H_2_O), 45 °C, 3 h; then CuBr_2_, TMEDA, r.t., 2 days, 21% over two steps.

To this end, the reaction of the TMS-protected triyne **6** with NaOMe in an ether–methanol mixture (4 : 1) resulted in the complete deacetylation and simultaneous desilylation, furnishing the corresponding glucoyslated triyne amphiphile **GLU3-H** after neutralization with Amberlite ion exchange resin (Scheme 4). The highly reactive amphiphile was not isolated but immediately added into an aqueous solution containing an excess of α-cyclodextrin at 45 °C. The resulting mixture was stirred for several hours in order to ensure the formation of the corresponding triyne pseudo-rotaxane. However, all attempts to isolate the pseudo-rotaxane failed in our hands, possibly due to de-threading of the α-cyclodextrin from the triyne upon work-up. Therefore, we decided to perform an *in situ* oxidative homocoupling under Glaser–Galbraith conditions with a solution of **GLU3-H** in the presence of α-cyclodextrin by the addition of CuBr_2_ and TMEDA followed by stirring of the mixture for 48 h at room temperature (Scheme 4).^65,66^ After purification by Sephadex column chromatography in water and preparative reverse-phase HPLC in water/acetonitrile, we thus obtained the corresponding [3]rotaxane **GLU6GLU·2 CD** in 21% isolated yield, which features two α-cyclodextrin hosts threaded onto one diglucosylated hexayne guest. For comparison, we also prepared the analogous free diglucosylated **TAG6TAG** by desilylation of **6** and simultaneous homocoupling with Cu(OAc)_2_. The latter was then converted into the deprotected hexayne **GLU6GLU** by deacetylation with NaOMe/MeOH (Scheme 4).

**Fig. 7:**
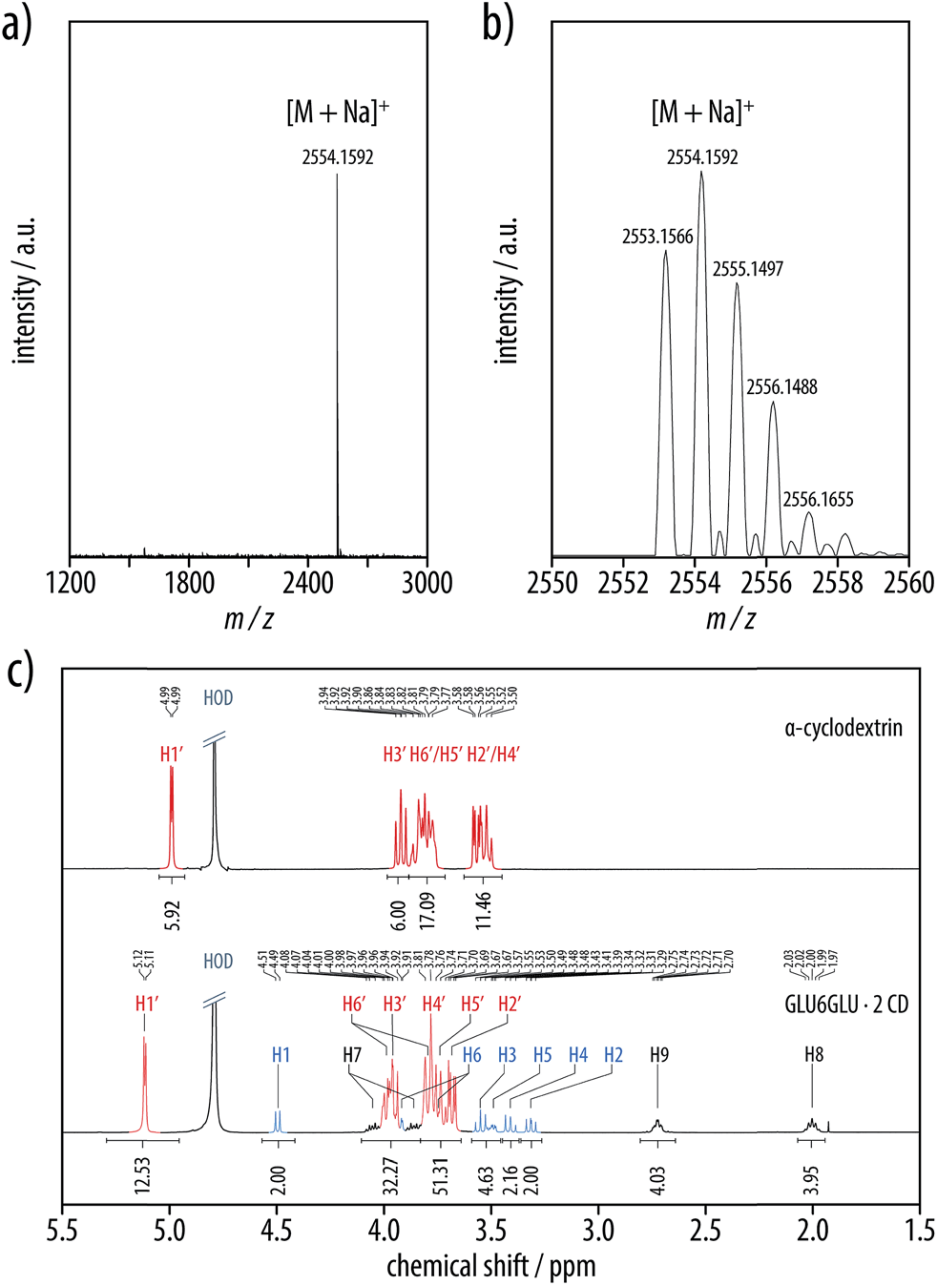
(a and b) MALDI-TOF mass spectrum of **GLU6GLU·2 CD**; the lowest mass peak of *m*/*z* 2553.2 Dalton ([M + Na]^+^) matched the expected monoisotopic mass of the proposed [3]rotaxane. (c) The comparison of the ^1^H-NMR spectra (400 MHz, 297.2 K, D_2_O) of the native α-cyclodextrin (top) and the [3]rotaxane **GLU6GLU·2 CD** (bottom) highlighted the shifts of the α-cyclodextrin proton signals that can be attributed to the threading onto the π-conjugated guest.

**Fig. 8:**
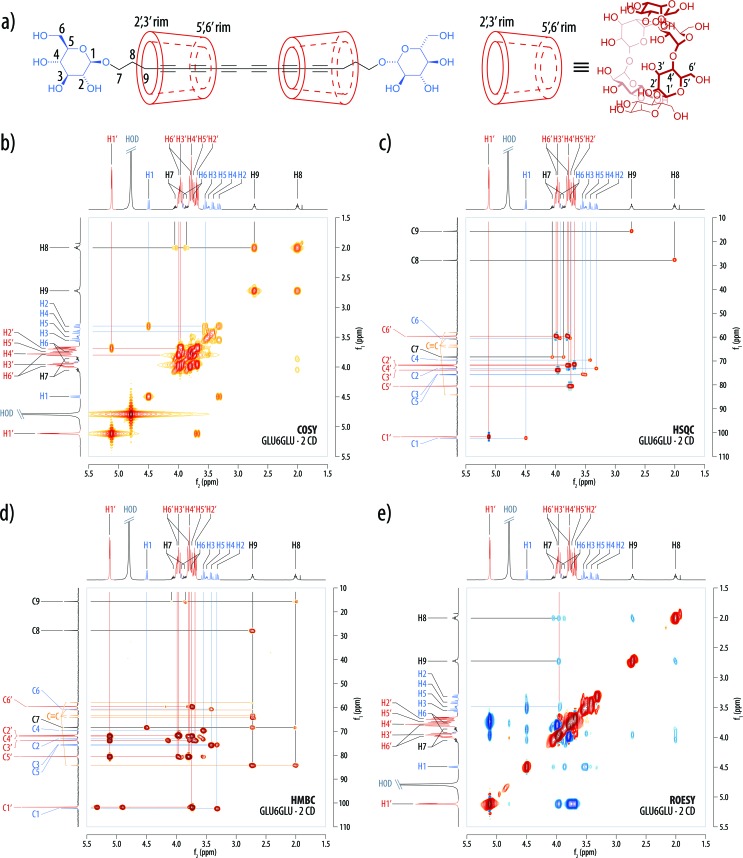
Structure of the [3]rotaxane **GLU6GLU·2 CD** and the corresponding 2D NMR spectra (all 400 MHz, 297.2 K, D_2_O). (a) The proposed structure features a tail-to-tail arrangement of the two threaded α-cyclodextrin hosts. The assignment of the signals in the ^1^H and ^13^C-NMR spectra was established by means of (b) the ^1^H,^1^H-COSY NMR spectrum, (c) the ^1^H,^13^C-HSQC NMR spectrum, and (d) the ^1^H,^13^C-HMBC NMR spectrum. (e) The ^1^H,^1^H-ROESY NMR spectrum of the [3]rotaxane **GLU6GLU·2 CD** revealed through-space contacts between H3′ of the wider 2,3-rim of the α-cyclodextrin host with the protons H9, H8, and H3 of the hexayne glycosyl host. See ESI Fig. S1–S4 for enlarged versions and further details.†

The structure of the [3]rotaxane **GLU6GLU·2 CD** was unambiguously proven by mass spectrometry and NMR spectroscopy. Thus, MALDI-TOF mass spectra exhibited a lowest mass peak with a matching monoisotopic mass of *m*/*z* 2553.2 Dalton ([M + Na]^+^) and the expected peak distribution (Fig. 7a and b). The ^1^H NMR spectra in D_2_O (Fig. 7c) showed the peaks of the α-cyclodextrin hosts and the amphiphilic hexayne guest with integration values (α-cyclodextrin/hexayne 2 : 1) consistent with the proposed molecular structure of **GLU6GLU·2 CD** (Fig. 8a). The anomeric H1′ signal of native α-cyclodextrin at *δ* = 4.99 ppm had been shifted downfield to *δ* = 5.11 ppm, which can be attributed to the threading of the α-cyclodextrin onto the π-conjugated guest. Moreover, the number of peaks in the ^13^C NMR spectra of the product was consistent with a symmetric (head-to-head or tail-to-tail) arrangement of the two threaded α-cyclodextrin hosts. All ^1^H and ^13^C NMR signals were unambiguously assigned by means of ^1^H,^1^H correlation spectroscopy (COSY), heteronuclear single quantum coherence (HSQC) spectroscopy, and heteronuclear multiple-bond correlation (HMBC) spectroscopy (Fig. 8b–d). Notably, the diastereotopic protons in the CH_2_ groups at C6 and C6′ of both the guest and host as well as the one at C7 of the propylene spacer showed a strong splitting not observed in the free molecules. Moreover, rotating-frame nuclear Overhauser effect correlation spectroscopy (ROESY) measurements proved through-space contacts between H3′ of the wider 2,3-rim of the α-cyclodextrin host with the two proton pairs H8 and H9 of the propylene spacers and H3 of the guest's glucosyl residue (Fig. 8e). Thus, the observed through-space contacts combined with the absence of desymmetrization in the 1D spectra allowed us to unambiguously conclude that the isolated product exclusively comprised the isomer with a tail-to-tail arrangement of the two threaded α-cyclodextrin hosts, while the other two possible isomers with a head-to-head or head-to-tail arrangement of the α-cyclodextrin hosts were absent. Although the low isolated yield of the **GLU6GLU·2 CD** product did not allow us to exclude the formation of the other isomers, a similarly specific formation of only one of the three possible isomers of a [3]rotaxane had already been demonstrated in previous examples of α-cyclodextrin host complexes with π-conjugated guests.^67,68^

**Fig. 9:**
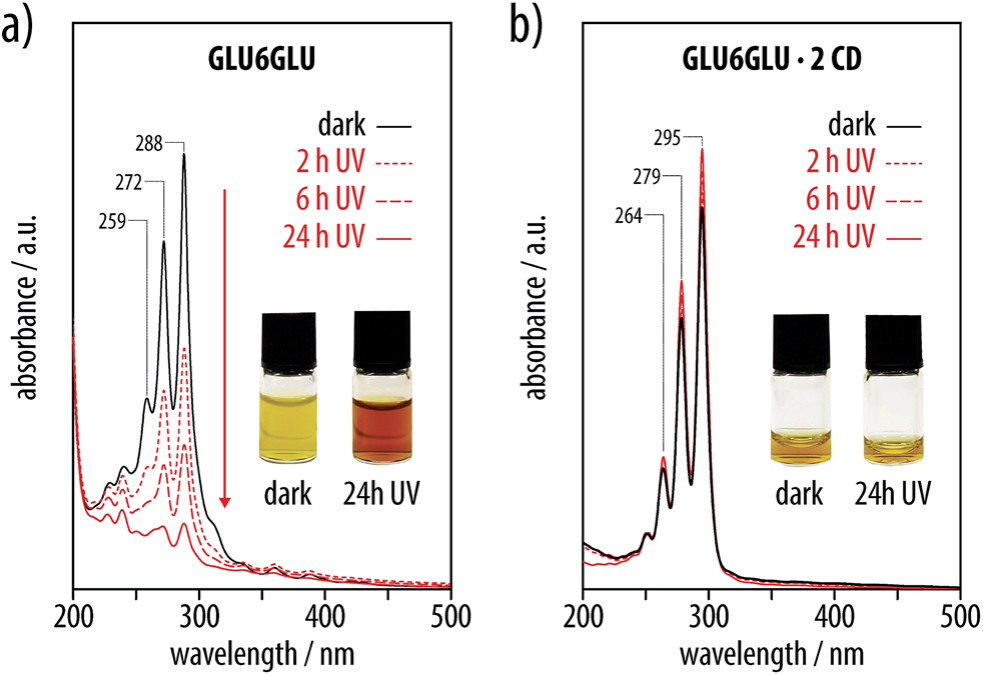
UV-vis spectra of aqueous solutions of (a) free hexayne **GLU6GLU** and (b) the corresponding [3]rotaxane **GLU6GLU·2 CD** upon UV irradiation after different irradiation times (ambient conditions, 250 W Ga-doped medium-pressure Hg lamp, 250–600 nm). While the unprotected hexayne moiety of **GLU6GLU** underwent photo-degradation, **GLU6GLU·2 CD** was stable under these conditions (the apparent increase in absorption is due to partial evaporation of the solvent).

In marked contrast to the free hexayne **GLU6GLU**, the corresponding [3]rotaxane **GLU6GLU·2 CD** was remarkably stable and did not show any signs of decomposition in aqueous solution or in bulk over months at room temperature, at elevated temperatures, or upon extended exposure to daylight. In order to further elucidate this stabilizing effect of the rotaxane formation, we subjected aqueous solutions of **GLU6GLU** and **GLU6GLU·2 CD** to intense UV irradiation using a 250 W Ga-doped medium pressure mercury lamp, and recorded their UV-vis spectra after different irradiation times (Fig. 9). As expected, the solution of **GLU6GLU** showed a drastic color change from yellow to brown over time, and the corresponding UV-vis spectra confirmed its decomposition. In marked contrast, solutions of the hexayne [3]rotaxane **GLU6GLU·2 CD** did not show a color change even after 24 h of UV irradiation. Moreover, the intensity, shape, and fine structure of its UV absorption band remained unaltered. Apparently, the α-cyclodextrin hosts served as a sheath and thus effectively prevented any photo-degradation or polymerization of the hexayne moieties.

## Conclusions

In conclusion, we developed a concise synthetic pathway that allowed us to prepare two novel series of unsymmetric oligoyne derivatives bearing functional groups up to the hexaynes on the multi-gram scale. The improved molecular design and the newly introduced acetylenic building blocks that complement our Negishi coupling protocol allowed for the efficient and concise elongation of the oligoyne segment, significantly facilitating the synthetic access to this class of compounds. Moreover, we were able to deprotect the obtained molecules to obtain reactive, carbon-rich amphiphiles with different polar head groups that self-assembled to form carbon-rich colloidal aggregates in aqueous dispersion. Furthermore, the amphiphilicity of the glucose oligoynes was used for the preparation of a water-soluble hexayne rotaxane with two threaded α-cyclodextrin hosts. This encapsulation in a host–guest complex was found to shield and stabilize the otherwise reactive oligoyne segments against photochemical degradation or cross-linking. The oligoyne amphiphiles described here may hence serve as carbon-rich precursors for the low-temperature preparation of novel types of carbon nanostructures from colloidal dispersions or self-assembled monolayers. Alternatively, their facile self-assembly into host–guest complexes in polar media represents a novel paradigm for the stabilization of compounds with conjugated triple bonds. This may pave the way for the preparation of shielded molecular wires based on oligoynes that may further serve as protected oligomer models for carbyne.
