# Supplementary material for: Facile synthesis of oligoyne amphiphiles and their rotaxanes
Source: Chem Sci. 2014 Oct 24;6(1):564–74. doi: 10.1039/c4sc03154g (PMC5492104; doi:10.1039/c4sc03154g)
Supplement: Supplementary file 1 [file SC-006-C4SC03154G-s001.pdf]

Electronic Supplementary Material (ESI) for Chemical Science.
This journal is © The Royal Society of Chemistry 2014

Facile Synthesis of Oligoyne Amphiphiles and their Rotaxanes

Electronic Supplementary Information

Stephen Schrettl,[1,‡] Emmanuel Contal,[1,‡] Tobias N. Hoheisel,[1,2] Martin Fritzsche,[1]

Sandor Balog,[3] Ruth Szilluweit,[1] Holger Frauenrath[1,]*

[1] Ecole Polytechnique Fédérale de Lausanne (EPFL)
Institute of Materials
Laboratory of Macromolecular and Organic Materials

EPFL – STI – IMX – LMOM
MXG 037, Station 12
1015 Lausanne, Switzerland

holger.frauenrath@epfl.ch

[2] ETH Zürich
Department of Materials
Vladimir-Prelog-Weg 1-5/10
8093 Zurich, Switzerland

[3] Adolphe Merklé Institute
Route de l'Ancienne Papeterie, CP209
1723 Marly, Switzerland

‡ both authors contributed equally to this publication

Table of Contents

1. Supplementary Figures S1–S4 2

2. Experimental Details 6

3. NMR Spectra 23

4. References 46

1. Supplementary Figures S1–S4

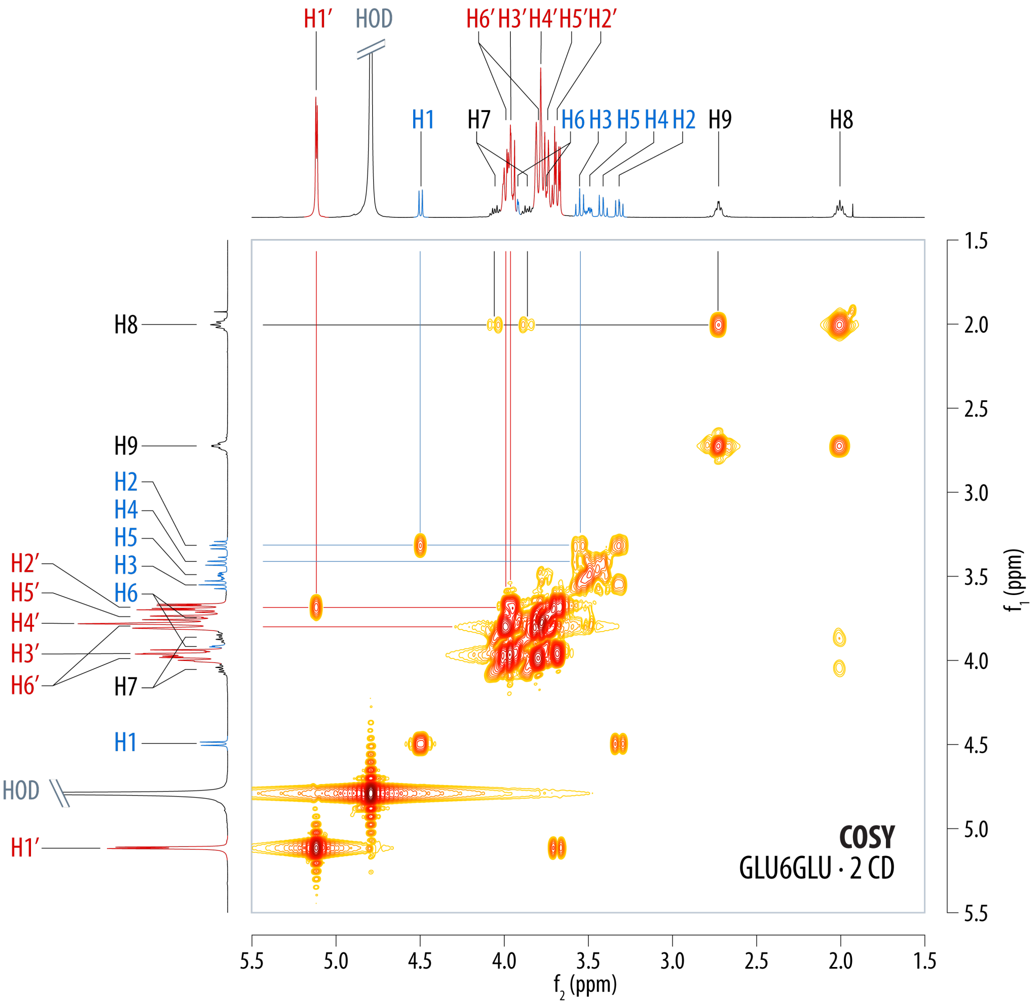

Supplementary Figure S1. $^{1}H,^{1}H$-COSY NMR spectrum (400 MHz, 297.2 K, D_2O) of the [3]rotaxane **GLU6GLU · 2 CD**. The cross-peaks observed between H8 and H7 indicated a strong splitting of the diastereotopic protons of the CH_2 group at H7, contrary to the free molecule **GLU6GLU**.

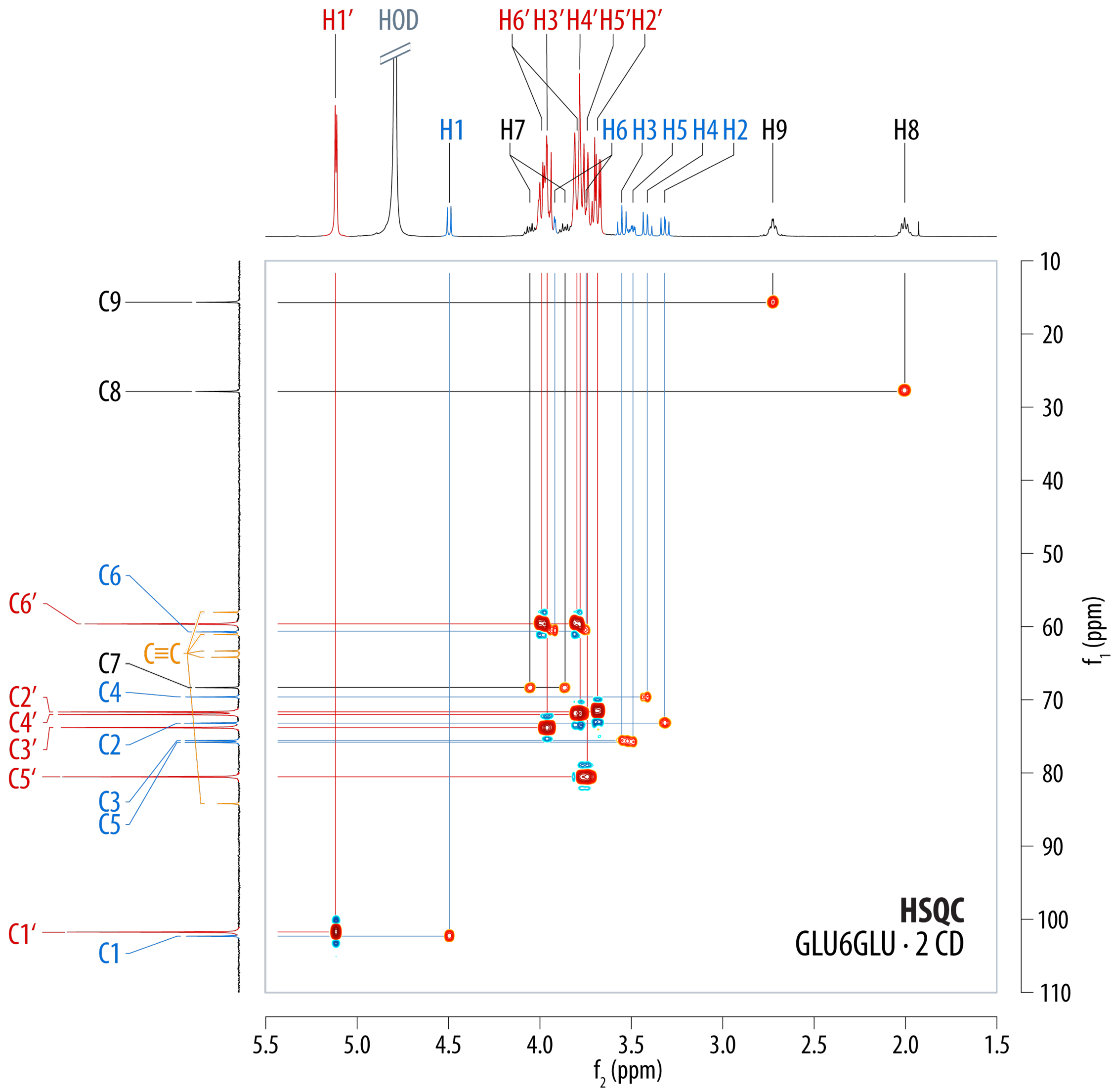

Supplementary Figure S2. $^{1}H,^{13}C$-HSQC NMR spectrum (400 MHz, 297.2 K, D_2O) of the [3]rotaxane **GLU6GLU · 2 CD**. The assignment of the signals was strongly corroborated by the cross-peaks in the HSQC spectrum; the splitting of the signal of the diastereotopic CH_2 protons H7 was confirmed, in addition to the splitting observed for the CH_2 proton signals of H6 (on the oligoyne guest) and H6’ (on the cyclodextrin host).

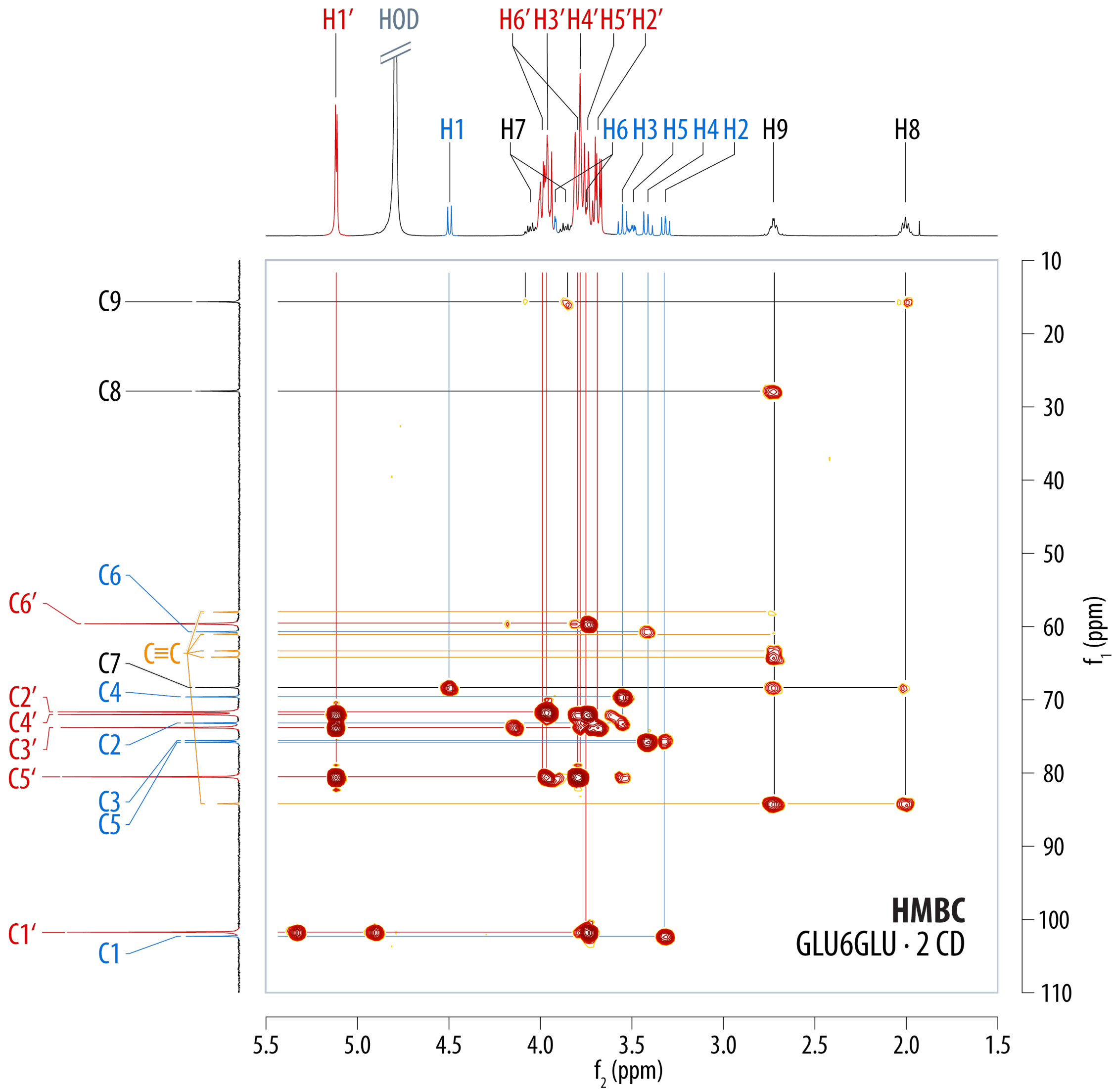

Supplementary Figure S3. $^{1}H,^{13}C$-HMBC NMR spectrum (400 MHz, 297.2 K, D_2O) of the [3]rotaxane **GLU6GLU · 2 CD**. The tentative assignment of the signals according to the $^{1}H,^{1}H$-COSY and $^{1}H,^{13}C$-HSQC NMR spectra was further confirmed by the cross-peaks observed in the HMBC spectrum, which also revealed the coupling of the propylene spacer CH_2 protons with the acetylenic carbons.

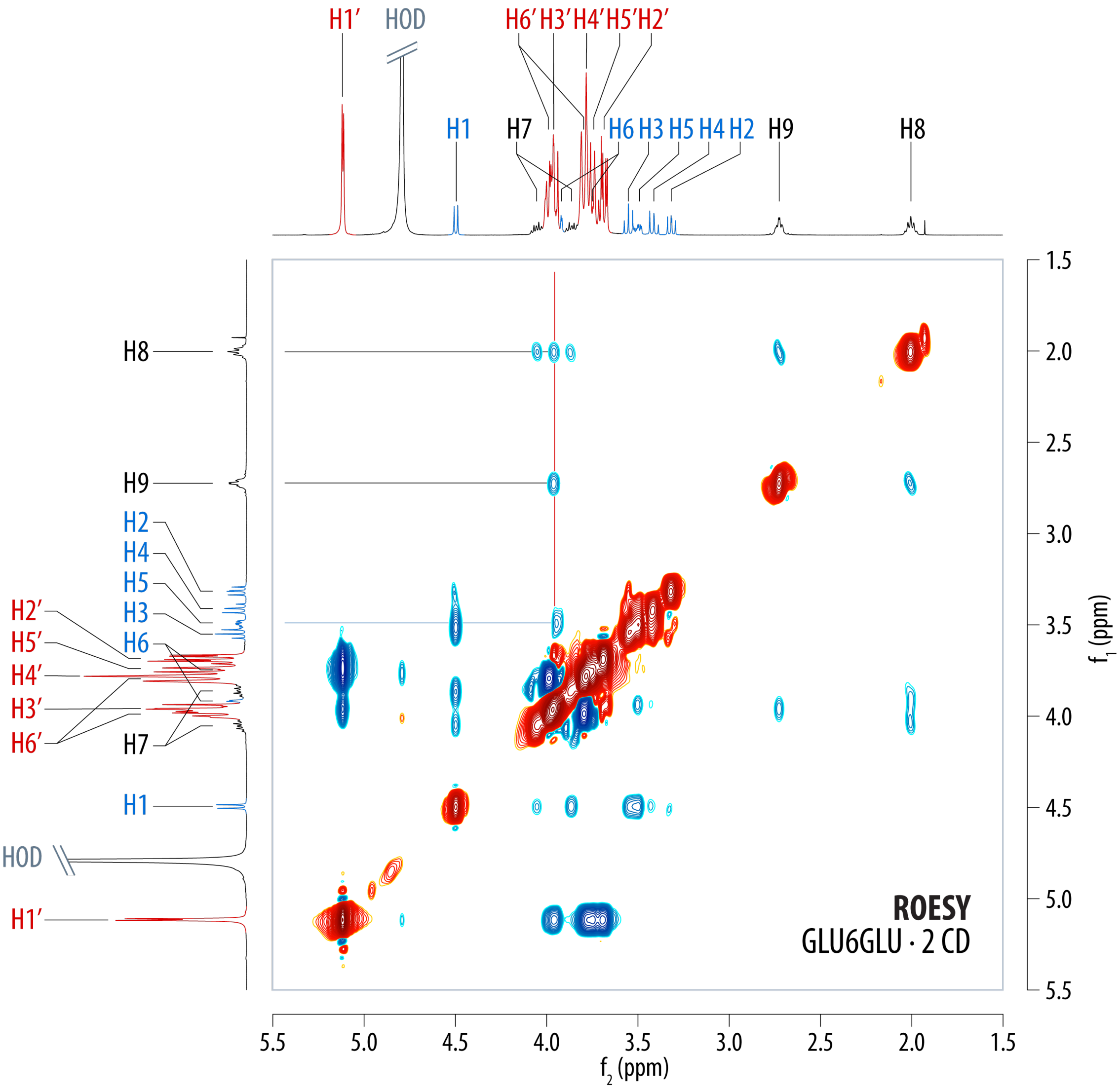

Supplementary Figure S4. $^{1}H,^{1}H$-ROESY NMR spectrum (400 MHz, 297.2 K, D_2O) of the [3]rotaxane **GLU6GLU · 2 CD**. In addition to multiple through-space contacts within the host and guest molecules, respectively, the ROESY NMR spectrum also revealed through-space contacts between H3' of the wider 2,3-rim of the α-cyclodextrin host with the protons H9, H8, and H3 of the hexayne guest.

2. Experimental Details

2.1 Instrumentation

NMR Spectroscopy was carried out at 297.2 K on Bruker Avance III 400 or 300 spectrometers at frequencies of 400.13 MHz or 300.23 MHz, respectively, for ^{1}H nuclei and 100.62 MHz or 75.49 MHz, respectively, for ^{13}C nuclei. Spectra were calibrated to the residual solvent peak of CD_2Cl_2 (5.32 ppm ^{1}H NMR; 54.00 ppm ^{13}C NMR), $CDCl_3$ (7.26 ppm ^{1}H NMR; 77.16 ppm ^{13}C NMR), DMSO-d_6 (2.50 ppm ^{1}H NMR; 39.52 ppm ^{13}C NMR) or D_2O (4.79 ppm ^{1}H NMR). Rotating frame ^{1}H,^{1}H nuclear overhauser effect spectroscopy (ROESY) was performed with a mixing time of 200 ms.

High resolution mass spectra were recorded as service measurements at the mass spectrometry service of the Institute of Chemical Sciences and Engineering at EPFL or at the mass spectrometry service of the Institute of Organic Chemistry at the Department of Chemistry and Applied Biosciences at ETH Zurich. Mass spectrometry at EPFL was carried out on either on a Shimadzu Biotech AXIMA-CFR plus for MALDI-TOF, a Waters Q-TOF Ultima for ESI-TOF, or a Thermo Scientific LTQ FT-ICR MS for APPI, whereas mass spectrometry at ETH Zurich was performed on an Ionspec Ultima 494 as well as a Bruker Daltonics maXis for HiRes-ESI-MS, and a Micromass Autospec device for HiRes-EI-MS.

Melting points were measured on a BÜCHI Melting Point M-560 device.

UV/Vis spectra were recorded on a JASCO V670 using 1 cm quartz cuvettes from Hellma. IR spectra were recorded on a JASCO FT/IR 6300 spectrometer using the Miracle Acessory from PIKE. Raman spectra were recorded on a JASCO FT/IR 6300 spectrometer equipped with an RFT-6000 Raman accessory with a 1064 nm laser. Optical rotation values were measured on a JASCO P-2000 at a wavelength of λ = 589 nm and with a path length of 10 cm.

Preparative reverse-phase high-performance liquid chromatography (HPLC) was performed on a Waters 600 automated gradient controller pump module, a Waters Prep Degasser, a Waters 2487 dual λ absorbance detector (at UV 220 and 330 nm, respectively), and a Waters Fraction Collector III with a preparative column (Waters Atlantis dC18 OBD 5 µm, 30 × 150 mm). Elution was carried out with a linear AB gradient, (*A*) dd H_2O, (*B*) MeCN containing 0.1 % v/v acetic!? acid (pH = 4) and a flow rate of 20 mL min^{-1}.

2.2 Materials and Methods

Preparation of Aqueous Dispersions. Aqueous dispersions of **GLU6-TIPS** and **MEC6-TIPS** were prepared by injecting stock solutions of the compounds in organic solvent (400 µL, 10 mg/mL in

1,4-dioxane/methanol 4:1) into MilliQ water (1600 µL). The obtained aqueous dispersions exhibited concentrations of 2 mg/mL of **GLU6-TIPS** or **MEC6-TIPS**. These dispersions were vortexed four times for 30 s every 10 min.

Cryogenic Transmission Electron Microscopy (Cryo-TEM). Cryo-TEM images were recorded on a CM12 EM electron microscope (Philips, Eindhoven, Netherlands) operating at 100 keV. The operation temperature was –180 °C, and a cryo-specimen holder Gatan 626 (Warrendale, PA, USA) was used. Digital micrographs were recorded with a Gatan MultiScan CCD-camera. Samples for cryo-TEM imaging were prepared by the 'plunge freezing' method. For this purpose, a holey or lacey carbon TEM grid was fixed between tweezers, and 5 µL of the sample dispersion were placed onto the grid. The tweezers were subsequently mounted into a 'plunge freezing' apparatus and, after blotting, the grid was immediately dropped into a container filled with liquid ethane that was surrounded by liquid nitrogen.

UV-Vis Spectroscopy with Aqueous Dispersions. For UV-Vis spectroscopy, the aqueous dispersions of **GLU6-TIPS** and **MEC6-TIPS** were further diluted with MilliQ water to a final concentration of 2 µg/mL (c = 3.83 µmol/L, 4.97 µmol/L, respectively). In order to prove the reversibility of the aggregation into colloidal aggregates, the 0.5 mL of the aqueous dispersions were subsequently diluted with 1.5 mL of MeCN, to a final concentration of c = 0.96 µmol/L and c = 1.24 µmol/L, respectively.

Dynamic Light Scattering (DLS). The size distribution of samples was determined by dynamic light scattering using a Nano-ZS (Malvern Instruments). For the measurements, the aqueous dispersions of **GLU6-TIPS** and **MEC6-TIPS** (c = 2 mg/mL, in dioxane/MeOH/water 4:1:20) were further diluted with MilliQ water (800 µL). The scattered intensity fluctuations were collected at a fixed angle of 173° by averaging 3 runs.

Depolarized Dynamic Light Scattering (DDLS). The DDLS measurements were performed at 21 °C, using a commercial goniometer instrument (3D LS Spectrometer, LS Instruments AG, Switzerland). The incident light was formed by a linearly polarized and collimated laser beam (HeNe, 632.8 nm, 21 mW), and the scattered light was collected by single-mode optical fibers having integrated collimation optics. Collected light was coupled into two high-sensitivity APD detectors (Perkin Elmer, Single Photon Counting Module), and their outputs were fed into a two-channel multiple-tau correlator (Correlator.com). The signal-to-noise ratio was improved by cross-correlating these two channels. With respect to the primary beam, depolarized scattering was observed via cross-polarizers. The incoming laser beam passed through a Glan-Thompson polarizer with an extinction ratio of 10^{-6}, and another Glan-Thompson polarizer, with an extinction ratio of 10^{-8}, was mounted in front of the collection optics.

UV Irradiation. The UV irradiation was carried out using a 250 W Ga-doped medium-pressure Hg lamp with multiple strong bands between wavelengths of 600–250 nm (UV-Light Technology, Birmingham, United Kingdom). The lamp was placed 50 cm away from the samples for the illumination.

2.3 Synthetic Procedures and Analytical Data

General Procedures. Unless otherwise noted, all reactions were carried out in dried Schlenk glassware in an inert argon atmosphere. Chromatography solvents were purchased as reagent grade and distilled once prior to use. For reactions, acetonitrile, dichloromethane, tetrahydrofuran, and toluene were purchased as HPLC grade and dried using a solvent purification system from Innovative Technologies. Diethyl ether and methanol were purchased dry over molecular sieves from Acros. All reagents were commercially obtained and used without further purification. β-D-Glucose pentaacetate (98%), boron trifluoride etherate (48% BF_3), *N*-bromosuccinimide (99%), *n*-butyl lithium (2.5 M in hexanes), methyl lithium lithium bromide complex (2.2 M in diethyl ether) and $ZnCl_2$ (0.7 M in THF) were purchased from Acros Organics, 4-pentyn-1-ol (97%) and silver nitrate (>99%) were purchased from Sigma-Aldrich, and $PdCl_2$(dppf) · DCM was purchased from Boron Molecular. TIPS-C≡C-H was purchased from Fluorochem. The acetylenic building blocks TIPS-$(C≡C)_2$-TMS,[1] TMS-$(C≡C)_2$-TMS,[2,3] and TIPS-$(C≡C)_3$-TMS[1] as well as 4-tritylphenol,[4 378–398] were synthesized according previously published procedures. The zinc acetylides TIPS-C≡C-ZnCl **1,** TIPS-$(C≡C)_2$-ZnCl **2a**, TMS-$(C≡C)_2$-ZnCl **2b**, and TIPS-$(C≡C)_3$-ZnCl **3** were prepared in situ as described in the procedures below. **Caution:** The stability of oligoyne derivatives is strongly dependent on the end groups; Although no explosions were encountered during the work described below, great care should be taken when carrying out similar reactions.

TLC analyses were performed on TLC plates from Merck (Silica gel 60 F_{254}). UV-light (254 nm) or anisaldehyde staining was used for detection. Column chromatography was conducted on Geduran silica gel Si 60 from Merck (40-60 µm) or on Acros ultrapure silica gel 60A (40-60 µm). For MALDI-TOF mass spectroscopy either α-cyano-4-hydroxycinnamic acid (CCA), 2,5-dihydroxybenzoic acid (DHB), or *trans*-2[3-(4-*tert*-butylphenyl)-2-methyl-2-propenylidene]malononitrile (DCTB) were used as the matrix materials.

Pent-4'-ynyl 2,3,4,6-tetra-*O*-acetyl-β-D-glucopyranoside 4. β-D-Glucose pentaacetate (47.25 g, 121.05 mmol) and 4-pentyn-1-ol (15.71 g, 186.76 mmol) were dissolved in DCM (600 mL). The mixture was cooled to 0°C, and boron trifluoride etherate (35.4 g, 249.44 mmol) was added. The cooling bath was removed, and the reaction was stirred for 7 h. The mixture was washed three times

with saturated aqueous $NaHCO_3$ solution and once with saturated aqueous NaCl solution. The organic phase was dried over $MgSO_4$, and column chromatography (silica gel, heptane/EtOAc 1:2) yielded **4** (23.55 g, 47%) as a colorless syrup which solidified after two days. ^{1}H NMR (400 MHz, CD_2Cl_2): δ = 5.19 (dd, *J* = 9.6 Hz, 1H, *H3*), 5.03 (dd, *J* = 9.9 Hz, 1H, *H4*), 4.91 (dd, *J* = 8.0 Hz, 9.7 Hz, 1H, *H2*), 4.51 (d, *J* = 8.0 Hz, 1H, *H1*), 4.24 (dd, *J* = 4.8 Hz, 12.3 Hz, 1H, *H6*), 4.09 (dd, *J* = 2.5 Hz, 12.3 Hz, 1H, *H6*), 3.92 (dt, *J* = 9.8 Hz, 5.7 Hz, 1H, OC*H*H), 3.71 (ddd, *J* = 10.0 Hz, 4.8 Hz, 2.5 Hz, 1H, *H5*), 3.62 (ddd, *J* = 9.8 Hz, 7.7 Hz, 5.4 Hz, 1H, OCH*H*), 2.23 (dt, *J* = 7.4 Hz, 2.7 Hz, 2H, CCCH_2), 2.05 (s, 3H, CH_3), 2.02 (s, 3H, CH_3), 1.99 (s, 3H, CH_3), 1.98 (t, *J* = 2.7 Hz, CC*H*), 1.96 (s, 3H, CH_3), 1.83-1.70 (m, 2H, CCCH$_2$CH_2). ^{13}C NMR (101 MHz, CD_2Cl_2) δ = 171.0, 170.5, 169.9, 169.8 (4 *C*=O), 101.5 (*C*1), 83.9 (*C*≡C), 73.2 (*C*3), 72.3 (*C5*), 71.8 (*C2*), 69.1 (*C4*), 69.0 (O*C*H$_2$), 69.0 (*C*≡C), 62.5 (*C6*), 28.9 (OCH$_2$*C*H$_2$), 21.0, 21.0, 21.0, 21.0 (4 CO*CH3*), 15.3 (CC*C*H$_2$). IR (ATR) ν 3290, 3273, 2957, 2886, 1737, 1430, 1367, 1253, 1219, 1169, 1056, 1031 (cm^{-1}). [a]$_D^{23}$ 126.7 (c 0.22, MeCN). HRMS (ESI): calcd $C_{19}H_{30}NO_{10}$: ([M+NH$_4$]$^+$) 432.1864; found 409.1870 R$_f$: 0.39 (EtOAc/*n*-heptane 1:1).

5'-Bromopent-4'-ynyl 2,3,4,6-tetra-*O*-acetyl-β-D-glucopyranoside 5. Pent-4'-ynyl 2,3,4,6-tetra-*O*-acetyl-β-D-glucopyranoside **4** (12.49 g, 30.15 mmol) was dissolved in MeCN (250 mL), and the flask was wrapped with aluminum foil. *N*-Bromosuccinimide (5.15 g, 31.60 mmol) and $AgNO_3$ (2.55 g, 15.01 mmol) were added, and the mixture was stirred for 15 h. The reaction was quenched by the addition of a concentrated aqueous KCl solution (1.33 g, 17.77 mmol KCl in 5 mL DI water). The mixture was filtered through a paper filter and precipitated into deionized water (3 L). The mixture was stirred for 2 d, and the precipitate was filtered and redissolved with acetone. The solution was concentrated, DCM was added, and the solution was washed once with water and once with saturated aqueous NaCl solution. The organic phase was dried over $MgSO_4$ and evaporated *in vacuo* to yield 5'-bromopent-4'-ynyl 2,3,4,6-tetra-*O*-acetyl-β-D-glucopyranoside **5** (13.77 g, 93%) as a colorless solid. ^{1}H NMR (400 MHz, CD_2Cl_2) δ = 5.19 (dd, *J* = 9.6 Hz, 1H, *H3*), 5.03 (dd, *J* = 9.7 Hz, 1H, *H4*), 4.91 (dd, *J* = 9.7, 8.0 Hz, 1H, *H2*), 4.50 (d, *J* = 8.0 Hz, 1H, *H1*), 4.24 (dd, *J* = 12.3, 4.9 Hz, 1H, *H6*), 4.09 (dd, *J* = 12.3, 2.5 Hz, 1H, *H6*), 3.90 (dt, *J* = 9.9, 5.6 Hz, 1H, OC*H*H), 3.72 (ddd, *J* = 10.0, 4.8, 2.5 Hz, 1H, *H5*), 3.59 (ddd, *J* = 9.8, 7.7, 5.4 Hz, 1H, OCH*H*), 2.27 (t, *J* = 7.0 Hz, 2H, CCCH_2), 2.05 (s, 3H, CH_3), 2.02 (s, 3H, CH_3), 1.99 (s, 3H, CH_3), 1.97 (s, 3H, CH_3), 1.83 – 1.68 (m, 2H, CCCH$_2$CH_2). ^{13}C NMR (101 MHz, CD_2Cl_2) δ = 171.0, 170.5, 169.9, 169.8 (4 *C*=O), 101.5 (*C*1), 79.9 (*C*≡C), 73.2 (*C*3), 72.3 (*C5*), 71.8 (*C2*), 69.0 (*C4*), 68.9 (O*C*H$_2$), 62.5 (*C6*), 38.5 (*C*≡C), 28.7 (OCH$_2$*C*H$_2$), 21.0, 21.0, 21.0, 21.0 (4 CO*C*H$_3$), 16.5 (CC*C*H$_2$). IR (ATR) ν 2965, 2943, 2882, 1740, 1431, 1370, 1221, 1166, 1056, 1032 (cm^{-1}). [α]$_D^{23}$ 148.4 (c 0.20, MeCN). HRMS (ESI): calcd for $C_{19}H_{25}BrO_{10}Na$ ([M+Na]$^+$) 515.0523; found 515.0530. R$_f$: 0.39 (EtOAc/n-heptane 1:1).

9'-Trimethylsilylnona-4',6',8'-triynyl 2,3,4,6-tetra-*O*-acetyl-β-D-glucopyranoside 6.

1,4-Bis(trimethylsilyl)butadiyne (8.08 g, 41.56 mmol) was dissolved in THF (65 mL), cooled to 0 °C,

and MeLi·LiBr complex (18.5 mL, 2.2 M in Et_2O, 40.70 mmol) was added. The cooling bath was removed and the mixture was stirred for 60 min before it was cooled to 0 °C again. $ZnCl_2$ (60 mL, 0.7 M in THF, 42.0 mmol) was added, the cooling bath was removed, and the resulting mixture was stirred for 60 min in order to form TMS-(C≡C)$_2$-ZnCl **2b**. In a second flask, 5'-bromopent-4'-ynyl 2,3,4,6-tetra-*O*-acetyl-β-D-glucopyranoside **5** (10.00 g, 20.27 mmol) was dissolved in dry toluene (300 mL), and $PdCl_2$(dppf)·DCM (1.66 g, 2.03 mmol) was added. The two solutions were combined at 0 °C, the flask was wrapped with aluminum foil, and the resulting mixture was stirred for 20 h at 0 °C before it was diluted with Et_2O, washed once with saturated NH_4Cl solution, and once with saturated NaCl solution. The organic phase was dried over $MgSO_4$ and concentrated *in vacuo*. Column chromatography (silica gel; EtOAc/*n*-heptane 1:2) yielded **6** (8.37 g, 77%) as a light brown solid. ^{1}H NMR (400 MHz, CD_2Cl_2) δ = 5.19 (dd, *J* = 9.6 Hz, 1H, *H3*), 5.03 (dd, *J* = 9.9 Hz, 1H, *H4*), 4.90 (dd, *J* = 9.7, 8.0 Hz, 1H, *H2*), 4.50 (d, *J* = 8.0 Hz, 1H, *H1*), 4.24 (dd, *J* = 12.3, 4.9 Hz, 1H, *H6*), 4.09 (dd, *J* = 12.3, 2.5 Hz, 1H, *H6*), 3.90 (dt, *J* = 9.9, 5.5 Hz, 1H, OC*H*H), 3.72 (ddd, *J* = 10.0, 4.9, 2.5 Hz, 1H, *H5*), 3.59 (ddd, *J* = 9.9, 7.8, 5.2 Hz, 1H, OCH*H*), 2.38 (t, *J* = 7.0 Hz, 2H, CCCH_2), 2.05 (s, 3H, CH_3), 2.02 (s, 3H, CH_3), 1.99 (s, 3H, CH_3), 1.96 (s, 3H, CH_3), 1.88 – 1.72 (m, 2H, CCCH_2CH_2), 0.19 (s, 9H, Si(CH_3)$_3$). ^{13}C NMR (101 MHz, CD_2Cl_2) δ = 170.9, 170.5, 169.9, 169.8 (4 *C*=O), 101.5 (*C*1), 88.4, 86.5, 80.7 (3 *C*≡C), 73.2 (*C*3), 72.4 (*C5*), 71.7 (*C2*), 69.0 (*C4*), 68.7 (O*C*H$_2$), 66.1, 62.6 (2 *C*≡C), 62.5 (*C6*), 60.4 (*C*≡C), 28.5 (OCH$_2$*C*H$_2$), 21.0, 21.0, 21.0, 21.0 (4 CO*C*H$_3$), 16.4 (CC*C*H$_2$), -0.3 (Si(*C*H$_3$)$_3$). IR (ATR) ν 2960, 2939, 2898, 2206, 2163, 2072, 1745, 1378, 1365, 1240, 1206, 1165, 1068, 1035 (cm^{-1}). UV (MeCN) λ_{max} (log ε) 220 (5.02), 209 (5.06), 200 (4.89) nm. $[a]_D^{23}$ 149.8 (c 0.21, MeCN). HRMS (ESI): calcd for $C_{26}H_{34}O_{10}SiNa$ ($[M+Na]^+$) 557.1813; found 557.1832. R_f: 0.40 (EtOAc/*n*-heptane 1:1).

9'-Bromonona-4',6',8'-triynyl 2,3,4,6-tetra-*O*-acetyl-β-D-glucopyranoside 7.

9'-Trimethylsilylnona-4',6',8'-triynyl 2,3,4,6-tetra-*O*-acetyl-β-D-glucopyranoside **6** (3.50 g, 6.55 mmol) was dissolved in dry MeCN (80 mL) and the flask was wrapped with aluminum foil. *N*-Bromosuccinimide (1.23 g, 6.87 mmol) and AgF (872 mg, 6.87 mmol) were added, and the resulting mixture was stirred for 3 h. The mixture was precipitated into deionized water (1 L), and the precipitate was filtered to yield **7** (2.86 g, 81%) as a yellow solid. ^{1}H NMR (400 MHz, CD_2Cl_2) δ = 5.19 (dd, *J* = 9.6 Hz, 1H, *H3*), 5.03 (dd, *J* = 9.7 Hz, 1H, *H4*), 4.90 (dd, *J* = 9.7, 8.0 Hz, 1H, *H2*), 4.50 (d, *J* = 8.0 Hz, 1H, *H1*), 4.24 (dd, *J* = 12.3, 4.9 Hz, 1H, *H6*), 4.09 (dd, *J* = 12.3, 2.4 Hz, 1H, *H6*), 3.90 (dt, *J* = 9.9, 5.5 Hz, 1H, OC*H*H), 3.72 (ddd, *J* = 10.0, 4.8, 2.5 Hz, 1H, *H5*), 3.59 (ddd, *J* = 9.9, 7.8, 5.2 Hz, 1H, OCH*H*), 2.37 (t, *J* = 7.0 Hz, 2H, CCCH_2), 2.05 (s, 3H, CH_3), 2.02 (s, 3H, CH_3), 1.99 (s, 3H, CH_3), 1.96 (s, 3H, CH_3), 1.89 – 1.71 (m, 2H, CCCH$_2$CH_2). ^{13}C NMR (101 MHz, CD_2Cl_2) δ = 170.9, 170.5, 169.9, 169.8 (4 *C*=O), 101.4 (*C*1), 79.7 (*C*≡C), 73.1 (*C*3), 72.4 (*C5*), 71.7 (*C2*), 68.9 (*C4*), 68.7 (O*C*H$_2$), 66.3, 65.9 (2 *C*≡C), 62.4 (*C6*), 60.6, 60.1, 40.4 (3 *C*≡C), 28.4 (OCH$_2$*C*H$_2$), 21.0, 21.0, 21.0, 21.0 (4 CO*C*H$_3$), 16.3 (CC*C*H$_2$). IR (ATR) ν 2958, 2931,

2878, 2218, 2186, 1738, 1426, 1373, 1232, 1206, 1161, 1092, 1066, 1052, 1035 (cm^{-1}). UV (MeCN) λ_{max} (log ε) 212 (4.97) nm. $[a]_D^{23}$ 127.75 (c 0.24, MeCN). HRMS (ESI): calcd for $C_{23}H_{25}BrO_{10}Na$ ($[M+Na]^+$) 563.0523; found 563.0526. R_f: 0.35 (EtOAc/*n*-heptane 1:1).

5-Tritylphenyl hex-5-ynoate 8. 4-Tritylphenol (20.00 g, 59.5 mmol) was dissolved in a mixture of dry toluene (100 mL) and dry DCM (100 mL). 5-Hexynoic acid (8.08 g, 71.4 mmol), 4-dimethylamino-pyridinium *p*-toluenesulfonate (0.87 g, 2.97 mmol), as well as 1-ethyl-3-(3-dimethylaminopropyl)-carbodiimide hydrochloride (14.86 g, 71.4 mmol) were successively added to this solution. The mixture was stirred at room temperature for 8 h, after which 4-tritylphenol was consumed according to TLC. After dilution with DCM (400 mL) the mixture was washed three times with 1 M HCl, once with saturated $NaHCO_3$ solution, and once with saturated NaCl solution. The organic phase was dried over Na_2SO_4, and concentrated *in vacuo*. Recrystallization (DCM/*n*-heptane 1:2) afforded 4-tritylphenyl hex-5-ynoate **8** (23.63 g, 54.9 mmol, 92%) as a colorless crystalline solid. ^1H NMR (400 MHz, $CDCl_3$): δ = 7.44-7.07 (m, 17H, Ph*H*), 7.06-6.87 (m, 2H, Ph*H*), 2.72 (t, *J* = 7.4 Hz, 2H, C_2H), 2.36 (td, *J* = 7.0, 2.7 Hz, 2H, C_4H), 2.02 (t, *J* = 2.7 Hz, 1H, ≡C*H*), 1.97 (p, *J* = 14.4, 7.4 Hz, 2H, C_3H). ^{13}C NMR (101 MHz, $CDCl_3$): δ = 171.7 (*C*OOPhTr), 148.7, 146.7, 144.4, 132.2, 131.2, 127.7, 126.1, 120.5 (24C, *C*-Ar), 83.2, 69.5 (2 *C*≡C), 64.7 (*C*Tr), 33.1 (*C*2), 23.6 (*C*3), 17.9 (*C*4). Mp: 174–175 °C. IR (ATR) ν 3260, 3082, 3053, 3030, 2975, 1747, 1591, 1491, 1441, 1383, 1339, 1254, 1203, 1168, 1134, 1081, 1035, 1014 (cm^{-1}). HRMS (MALDI): calcd for $C_{31}H_{26}O_2Na$: ($[M+Na]^+$) 453.1826; found: 453.1851. R_f : 0.56, (EtOAc/*n*-heptane 1:1), 0.50 (DCM/*n*-heptane 2:1).

4-Tritylphenyl 6-bromohex-5-ynoate 9. 4-Tritylphenyl hex-5-ynoate **8** (20.0 g, 46.5 mmol) was dissolved in dry DCM (200 mL), and dry MeCN was added (200 mL). The flask was shielded from light with aluminum foil, and *N*-bromosuccinimide (8.69 g, 48.8 mmol) as well as silver nitrate (2.37 g, 13.9 mmol) were successively added. The resulting mixture was stirred at room temperature for 3 h after which it was diluted with DCM, washed four times with 1 M HCl, and once with saturated NaCl solution. The organic phase was dried over Na_2SO_4 and concentrated *in vacuo*. Column chromatography (silica gel; DCM/*n*-heptane 2:1) afforded 4-tritylphenyl 6-bromohex-5-ynoate **9** (22.78 g, 44.7 mmol, 96%) as an off-white solid. ^1H NMR (400 MHz, $CDCl_3$): δ = 7.33-7.16 (m, 17H, Ph*H*), 7.08-6.94 (m, 2H, Ph*H*), 2.71 (t, *J* = 7.4 Hz, 2H, C_2H), 2.39 (t, *J* = 6.9 Hz, 2H, C_4H), 1.97 (p, *J* = 7.1 Hz, 2H, C_3H). ^{13}C NMR (101 MHz, $CDCl_3$): δ = 171.6 (*C*OOPhTr), 148.7, 146.7, 144.5, 132.2, 131.2, 127.7, 126.1, 120.4 (24C, *C*-Ar), 79.1 (≡*C*), 64.7 (*C*Tr), 39.3 (≡*C*), 33.1 (*C*2), 23.5 (*C*3), 19.2 (*C*4). Mp: 124–125 °C. IR (ATR) ν 3085, 3055, 3029, 2940, 2909, 1755, 1594, 1492, 1444, 1409, 1370, 1311, 1265, 1204, 1169, 1083, 1034, 1017 (cm^{-1}). HRMS (MALDI): calcd for $C_{31}H_{26}O_2Na$: ($[M+Na]^+$) 531.0930; found: 531.0923. R_f : 0.64 (DCM/*n*-heptane 2:1).

4-Tritylphenyl 10-(trimethylsilyl)deca-5,7,9-triynoate 10. MeLi · LiBr complex (17.9 mL, 2.2 M in Et_2O, 39.26 mmol) was added to 1,4-bis(trimethylsilyl)butadiyne (7.82 g, 40.24 mmol) in THF (50 mL) at 0 °C, and the resulting mixture was stirred for 30 minutes. Then $ZnCl_2$ (57.5 mL, 0.7 M in THF, 40.24 mmol) was added at 0 °C, and the resulting mixture was again stirred for 30 min in order to form TMS-(C≡C)$_2$-ZnCl **2b**. In another flask, 4-tritylphenyl 6-bromohex-5-ynoate **9** (10.0 g, 19.63 mmol) and $PdCl_2$(dppf) · DCM (1.60 g, 1.96 mmol) were mixed in toluene (300 mL). The two solutions were combined at 0 °C, and the flask was wrapped with aluminum foil. The mixture was stirred for 16 h while warming up to room temperature, before it was diluted with Et_2O, washed three times with saturated NH_4Cl solution and once with saturated NaCl solution. The organic phase was dried over Na_2SO_4 and concentrated *in vacuo*. Column chromatography (silica gel; DCM/*n*-heptane 1:1) yielded **10** (7.7 g, 71%) as a light brown solid. ^{1}H NMR (400 MHz, $CDCl_3$): δ = 7.30-7.16 (m, 17H), 7.01-6.95 (m, 2H), 2.69 (t, J = 7.3 Hz, 2H), 2.47 (t, J = 6.9 Hz, 2H), 1.98 (p, J = 7.0 Hz, 2H), 0.20 (s, 9H). ^{13}C NMR (101 MHz, $CDCl_3$): δ = 171.4, 148.6, 146.7, 144.5, 132.3, 131.2, 127.7, 126.1, 120.4, 88.3, 86.1, 79.3, 66.7, 64.7, 62.3, 60.6, 33.0, 23.2, 19.0, –0.3. Mp: 119–121°C. IR (ATR) ν 3086, 3056, 3030, 2956, 2922, 2851, 2210, 2168, 2079, 1758, 1595, 1492, 1444, 1409, 1369, 1335, 1308, 1251, 1205, 1170, 1130, 1018 (cm^{-1}). UV (cyclohexane) λ_{max} (log ε) 221 (5.02), 212 (5.02) nm. HRMS (MALDI): calcd for $C_{38}H_{34}O_2SiNa$: 573.2219 ($[M+Na]^+$); found: 573.2201. R_f: 0.33 (DCM/*n*-heptane 1:1).

4-Tritylphenyl 10-bromodeca-5,7,9-triynoate 11. 4-Tritylphenyl 10-(trimethylsilyl)-deca-5,7,9-triynoate **10** (0.493 g, 0.908 mmol) was dissolved in DCM (12 mL) and acetonitrile (10 mL) was added. The flask was wrapped in aluminum foil, and *N*-bromosuccinimide (0.172 g, 0.953 mmol) as well as silver fluoride (0.122 g, 0.953 mmol) were added at room temperature. After 6 h, the reaction mixture was transferred into a separatory funnel and diluted with DCM (30 mL). The organic phase was washed six times with 1 M HCl (100 mL) and once with saturated NaCl solution. The organic phase was dried over Na_2SO_4, and concentrated to approximately 10 mL while thoroughly shielding it from light. For synthetic purposes, toluene was added, and the mixture was concentrated *in vacuo*. For analytic purposes, $CDCl_3$ (10 mL) was added, and the mixture was concentrated *in vacuo*. ^{1}H NMR (400 MHz, $CDCl_3$): δ = 7.30-7.20 (m, 17H), 7.02-6.98 (m, 2H), 2.71 (t, J = 7.3 Hz, 2H), 2.48 (t, J = 6.9 Hz, 2H), 2.00 (tt, J = 7.1 Hz, 2H). ^{13}C NMR (101 MHz, $CDCl_3$): δ = 171.2, 148.5, 146.6, 144.5, 132.2, 130.1, 127.6, 126.1, 120.4, 78.3, 66.4, 66.1, 64.7, 60.8, 59.6, 53.6, 39.9, 33.0, 23.1, 18.8. HRMS (MALDI): calcd for $C_{35}H_{25}BrNaO_2$: 581.0927 ($[M+Na]^+$); found: 581.0914.

7’-Triisopropylsilylhepta-4’,6’-diynyl 2,3,4,6-tetra-*O*-acetyl-β-D-glucopyranoside TAG2. Ethynyl-triisopropylsilane (0.82 mL, 3.65 mmol) was dissolved in THF (10 mL). The mixture wascooled to 0 °C, and *n*-butyl lithium (1.42 mL, 2.5 M in n-hexane, 3.55 mmol) was added. The cooling bath was removed, and the mixture was stirred for 30 min before it was cooled to 0 °C again. $ZnCl_2$

(5.08 mL, 0.7 M in THF, 3.55 mmol) was added, the cooling bath was removed, and the resulting mixture was stirred for 30 min in order to form TIPS-C≡C-ZnCl **1**. In a second flask, 5'-bromopent-4'-ynyl 2,3,4,6-tetra-*O*-acetyl-β-D-glucopyranoside **5** (1.00 g, 2.03 mmol) was dissolved in dry toluene (30 mL), and $PdCl_2$(dppf) · DCM (66 mg, 0.102 mmol) was added. The two solutions were combined at 0 °C, the flask was wrapped with aluminum foil, and the resulting mixture was stirred over night at 0 °C. The following day, [1,1'-bis(di*tert*.-butyl phosphino)ferrocene]palladiumdichloride (10 mg, 0.015 mmol) was added. The mixture was stirred for 3 h at 0 °C, before more [1,1'-bis(di*tert*.-butyl phosphino)ferrocene] palladiumdichloride (11 mg, 0.017 mmol) was added, and the resulting mixture was stirred for 4 d at room temperature. Then, the mixture was diluted with Et_2O, washed once with saturated NH_4Cl solution, and once with saturated NaCl solution. The organic phase was dried over $MgSO_4$ and concentrated *in vacuo*. Column chromatography (silica gel, EtOAc/n-heptane 1:2) yielded **TAG2** (971 mg, 87%) as a yellow oil, which solidified after two weeks. ^{1}H NMR (400 MHz, CD_2Cl_2) δ = 5.19 (dd, *J* = 9.6 Hz, 1H, *H3*), 5.03 (dd, *J* = 10.0 Hz, 1H, *H4*), 4.91 (dd, *J* = 9.7, 8.0 Hz, 1H, *H2*), 4.52 (d, *J* = 8.0 Hz, 1H, *H1*), 4.25 (dd, *J* = 12.3, 4.9 Hz, 1H, *H6*), 4.09 (dd, *J* = 12.3, 2.5 Hz, 1H, *H6*), 3.90 (dt, *J* = 9.9, 5.6 Hz, 1H, OC*H*H), 3.72 (ddd, *J* = 10.0, 4.9, 2.5 Hz, 1H, *H5*), 3.62 (ddd, *J* = 9.9, 7.8, 5.3 Hz, 1H, OCH*H*), 2.36 (t, *J* = 7.0 Hz, 2H, CCCH_2), 2.05 (s, 3H, CH_3), 2.03 (s, 3H, CH_3), 1.99 (s, 3H, CH_3), 1.97 (s, 3H, CH_3), 1.86 – 1.72 (m, 2H, CCCH_2CH_2), 1.07 (s, 21H, Si(C*H*(CH_3)$_2$)$_3$). ^{13}C NMR (101 MHz, CD_2Cl_2) δ = 171.0, 170.5, 169.9, 169.8 (4 *C*=O), 101.5 (*C*1), 90.5, 80.9, 78.4 (3 *C*≡C), 73.2 (*C*3), 72.4 (*C5*), 71.8 (*C2*), 69.0 (*C4*), 68.9 (O*C*H_2), 66.5 (*C*≡C), 62.5 (*C6*), 28.7 (OCH_2*C*H_2), 21.1, 21.0, 21.0, 21.0 (4 CO*C*H_3), 18.9 (Si(CH(*C*H_3)$_2$)$_3$), 16.2 (CC*C*H_2), 11.8 (Si(*C*H(CH_3)$_2$)$_3$). IR (ATR) ν 2945, 2864, 2225, 2104, 1741, 1462, 1428, 1365, 1255, 1225, 1168, 1130, 1090, 1060, 1036 (cm^{-1}). UV (MeCN, *c* = 4.21 μmol/L) λ_{max} (log ε) 196 (4.65) nm. $[a]_D^{23}$ 123.00 (c 0.25, MeCN). HRMS (ESI): calcd. for $C_{30}H_{50}NO_{10}Si$ ($[M+NH_4]^+$) 612.3198; found 612.3197. R_f: 0.45 (EtOAc/*n*-heptane 1:1).

9'-Triisopropylsilylnona-4',6',8'-triynyl 2,3,4,6-tetra-*O*-acetyl-β-D-glucopyranoside TAG3. 1-Triisopropylsilyl-4-trimethysilylbutadiyne (1.02 g, 3.65 mmol) was dissolved in THF (10 mL), cooled to 0 °C, and MeLi·LiBr complex (1.61 mL, 2.2 M in Et_2O, 3.55 mmol) was added. The cooling bath was removed and the resulting mixture was stirred for 30 min before it was cooled to 0 °C again. $ZnCl_2$ (5.08 mL, 0.7 M in THF, 3.55 mmol) was added, the cooling bath was removed, and the resulting mixture was stirred for 30 min in order to form TIPS-(C≡C)$_2$-ZnCl **2a**. In a second flask, 5'-bromopent-4'-ynyl 2,3,4,6-tetra-*O*-acetyl-β-D-glucopyranoside **5** (1.00 g, 2.03 mmol) was dissolved in dry toluene (30 mL), and $PdCl_2$(dppf)·DCM (83 mg, 0.102 mmol) was added. The two solutions were combined at 0 °C, the flask was wrapped with aluminum foil, the resulting mixture was stirred for 6 h at 0 °C and allowed to warm to room temperature over night. Then, the mixture was diluted with Et_2O, washed once with saturated NH_4Cl solution, and once with saturated NaCl solution. The organic phase

was dried over $MgSO_4$ and concentrated *in vacuo*. Column chromatography (silica gel; EtOAc/*n*-heptane 1:2) yielded **TAG3** (974 mg, 78%) as a light brown solid. ^{1}H NMR (400 MHz, CD_2Cl_2) δ = 5.19 (dd, *J* = 9.6 Hz, 1H, *H3*), 5.03 (dd, *J* = 10.0 Hz, 1H, *H4*), 4.91 (dd, *J* = 9.7, 8.0 Hz, 1H, *H2*), 4.50 (d, *J* = 8.0 Hz, 1H, *H1*), 4.24 (dd, *J* = 12.3, 4.9 Hz, 1H, *H6*), 4.10 (dd, *J* = 12.3, 2.5 Hz, 1H, *H6*), 3.90 (dt, *J* = 9.9, 5.5 Hz, 1H, OC*H*H), 3.72 (ddd, *J* = 10.0, 4.9, 2.5 Hz, 1H, *H5*), 3.60 (ddd, *J* = 9.9, 7.8, 5.2 Hz, 1H, OCH*H*), 2.38 (t, *J* = 6.9 Hz, 2H, $CCCH_2$), 2.05 (s, 3H, CH_3), 2.02 (s, 3H, CH_3), 1.99 (s, 3H, CH_3), 1.97 (s, 3H, CH_3), 1.88 – 1.71 (m, 2H, $CCCH_2CH_2$), 1.11 – 1.02 (m, 21H, $Si(CH(CH_3)_2)_3$)). ^{13}C NMR (101 MHz, CD_2Cl_2) δ = 171.0, 170.5, 169.9, 169.8 (4 *C*=O), 101.5 (*C*1), 90.2, 84.1, 80.3 (3 *C*≡C), 73.2 (*C*3), 72.4 (*C5*), 71.8 (*C2*), 69.0 (*C4*), 68.7 (*C*≡C), 66.1 (OCH_2), 62.5 (*C6*), 61.6, 60.8 (2 *C*≡C), 28.5 (OCH_2CH_2), 21.0, 21.0, 21.0, 21.0 (4 $COCH_3$), 18.8 ($Si(CH(CH_3)_2)_3$), 16.4 ($CCCH_2$), 11.8 ($Si(CH(CH_3)_2)_3$). IR (ATR) ν 2948, 2867, 2210, 2163, 2074, 1748, 1460, 1432, 1366, 1239, 1206, 1165, 1100, 1067, 1032 (cm^{-1}). UV (MeCN, *c* = 3.65 µmol/L) λ_{max} (log ε) 222 (4.96), 213 (4.95) nm. $[a]_D^{23}$ 139.34 (c 0.23, MeCN). HRMS (ESI): calcd. for $C_{32}H_{50}NO_{10}Si$ ($[M+NH_4]^+$) 636.3198; found 636.3214. R_f: 0.45 (EtOAc/*n*-heptane 1:1).

11'-Triisopropylsilylundeca-4',6',8',10'-tetraynyl 2,3,4,6-tetra-*O*-acetyl-β-D-glucopyranoside TAG4. 1-Triisopropylsilyl-6-trimethysilylhexatriyne (1.15 g, 3.8 mmol) was dissolved in THF (10 mL), cooled to 0 °C, and MeLi·LiBr complex (1.61 mL, 2.2 M in Et_2O, 3.55 mmol) was added. The cooling bath was removed and the resulting mixture was stirred for 30 min before it was cooled to 0 °C again. $ZnCl_2$ (5.08 mL, 0.7 M in THF, 3.55 mmol) was added, the cooling bath was removed, and the resulting mixture was stirred for 30 min in order to form TIPS-$(C{\equiv}C)_3$-ZnCl **3**. In a second flask, 5'-bromopent-4'-ynyl 2,3,4,6-tetra-*O*-acetyl-β-D-glucopyranoside **5** (1.00 g, 2.03 mmol) was dissolved in dry toluene (30 mL), and $PdCl_2(dppf)$·DCM (83 mg, 0.102 mmol) was added. The two solutions were combined at 0 °C, the flask was wrapped with aluminum foil, the resulting mixture was stirred for 6 h at 0 °C and allowed to warm to room temperature over night. After 4 d at room temperature, the mixture was diluted with Et_2O, washed once with saturated NH_4Cl solution, and once with saturated NaCl solution. The organic phase was dried over $MgSO_4$ and concentrated in vacuo. Column chromatography (silica gel; EtOAc/*n*-heptane 1:2) yielded **TAG4** (666 mg, 51%) as a brown foam. ^{1}H NMR (400 MHz, CD_2Cl_2) δ = 5.19 (dd, *J* = 9.6 Hz, 1H, *H3*), 5.03 (dd, *J* = 10.0 Hz, 1H, *H4*), 4.91 (dd, *J* = 9.7, 8.0 Hz, 1H, *H2*), 4.51 (d, *J* = 8.0 Hz, 1H, *H1*), 4.24 (dd, *J* = 12.3, 4.9 Hz, 1H, *H6*), 4.10 (dd, *J* = 12.3, 2.5 Hz, 1H, *H6*), 3.90 (dt, *J* = 9.9, 5.5 Hz, 1H, OC*H*H), 3.72 (ddd, *J* = 10.0, 4.9, 2.5 Hz, 1H, *H5*), 3.60 (ddd, *J* = 9.9, 7.8, 5.1 Hz, 1H, OCH*H*), 2.40 (t, *J* = 6.9 Hz, 2H, $CCCH_2$), 2.05 (s, 3H, CH_3), 2.02 (s, 3H, CH_3), 2.00 (s, 3H, CH_3), 1.97 (s, 3H, CH_3), 1.89 – 1.73 (m, 2H, $CCCH_2CH_2$), 1.17 – 1.00 (m, 21H, $Si(CH(CH_3)_2)_3$)). ^{13}C NMR (101 MHz, CD_2Cl_2) δ = 171.0, 170.5, 169.9, 169.8 (4 *C*=O), 101.5 (*C*1), 90.0, 85.9, 81.4 (3 *C*≡C), 73.2 (*C*3), 72.4 (*C5*), 71.8 (*C2*), 69.0 (*C4*), 68.7 (*C*≡C), 66.2 (OCH_2), 62.8 (*C6*), 62.5, 61.7, 61.7, 60.7 (4 *C*≡C), 28.4 (OCH_2CH_2), 21.0, 21.0, 21.0, 21.0 (4 $COCH_3$), 18.8 ($Si(CH(CH_3)_2)_3$), 16.5 ($CCCH_2$), 11.8 ($Si(CH(CH_3)_2)_3$). IR

(ATR) ν 2944, 2866, 2211, 2139, 2059, 1748, 1461, 1428, 1365, 1214, 1169, 1032 (cm^{-1}). UV (MeCN, c = 3.04 µmol/L) λ_{max} (log ε) 251 (5.18), 240 (5.06), 226 (4.82) nm. $[a]_D^{23}$ 159.94 (c 0.20, MeCN). HRMS (ESI): calcd for $C_{34}H_{50}NO_{10}Si$ ($[M+NH_4]^+$) 660.3198; found 660.3204. R_f: 0.45 (EtOAc/*n*-heptane 1:1).

13′-Triisopropylsilylundeca-4′,6′,8′,10′,12′-pentaynyl 2,3,4,6-tetra-*O*-acetyl-β-D-gluco-pyranoside TAG5. 1-Triisopropylsilyl-4-trimethysilylbutadiyne (0.99 g, 3.55 mmol) was dissolved in dry THF (8 mL), cooled to 0 °C, and MeLi·LiBr complex (1.60 mL, 2.2 M in Et_2O, 3.52 mmol) was added. The cooling bath was removed and the resulting mixture was stirred for 30 min before it was cooled to 0 °C again. $ZnCl_2$ (5.18 mL, 0.7 M in THF, 3.63 mmol) was added, the cooling bath was removed, and the resulting mixture was stirred for 30 min in order to form TIPS-(C≡C)$_2$-ZnCl **2a**. In a second flask, 9’-bromonona-4’,6’,8’-triynyl 2,3,4,6-tetra-*O*-acetyl-β-D-glucopyranoside **7** (1.02 g, 1.88 mmol) was dissolved in dry toluene (30 mL), and $PdCl_2$(dppf)·DCM (75 mg, 0.09 mmol) was added. The two solutions were combined at 0 °C, the flask was wrapped with aluminum foil, and the resulting mixture was stirred for 6 h at 0 °C and allowed to warm to room temperature over night. After 30 h at room temperature, the mixture was diluted with Et_2O, washed once with saturated NH_4Cl solution, and once with saturated NaCl solution. The combined aqueous phases were extracted once with Et_2O, and the combined organic phases were dried over $MgSO_4$ and concentrated *in vacuo*. Column chromatography (silica gel; EtOAc/*n*-heptane 1:2) yielded **TAG5** (0.52 g, 42%) as a light brown syrup. ^{1}H NMR (400 MHz, CD_2Cl_2) δ = 5.19 (dd, J = 9.6 Hz, 1H, *H3*), 5.03 (dd, J = 9.9 Hz, 1H, *H4*), 4.91 (dd, J = 9.7, 8.0 Hz, 1H, *H2*), 4.51 (d, J = 8.0 Hz, 1H, *H1*), 4.24 (dd, J = 12.3, 4.9 Hz, 1H, *H6*), 4.10 (dd, J = 12.3, 2.4 Hz, 1H, *H6*), 3.90 (dt, J = 10.0, 5.5 Hz, 1H, OC*H*H), 3.72 (ddd, J = 10.0, 4.9, 2.5 Hz, 1H, *H5*), 3.60 (ddd, J = 9.9, 7.8, 5.1 Hz, 1H, OCH*H*), 2.42 (t, J = 7.0 Hz, 2H, CCCH_2), 2.05 (s, 3H, CH_3), 2.02 (s, 3H, CH_3), 2.00 (s, 3H, CH_3), 1.97 (s, 3H, CH_3), 1.87–1.76 (m, 2H, CCCH$_2$CH_2), 1.13–1.04 (m, 21H, Si(C*H*(CH_3)$_2$)$_3$)). ^{13}C NMR (101 MHz, CD_2Cl_2) δ = 170.9, 170.5, 169.9, 169.8 (4 *C*=O), 101.5 (*C*1), 89.8, 87.1, 81.2 (3 *C*≡C), 73.2 (*C*3), 72.4 (*C5*), 71.8 (*C2*), 69.0 (*C4*), 68.6 (*C*≡C), 66.1 (O*C*H$_2$), 63.1, 62.9, 62.5 (3 *C*≡C), 62.4 (*C6*), 61.8, 61.6, 60.7 (3 *C*≡C), 28.4 (OCH$_2$*C*H$_2$), 21.1, 21.0, 21,0, 21.0 (4 CO*C*H$_3$), 18.8 (Si(CH(*C*H$_3$)$_2$)$_3$), 16.5 (CC*C*H$_2$), 11.8 (Si(*C*H(CH$_3$)$_2$)$_3$). IR (ATR) ν 2943, 2866, 2212, 2102, 2047, 1748, 1460, 1429, 1366, 1215, 1170, 1035 (cm^{-1}). UV (MeCN, c = 3.19 µmol/L) λ_{max} (log ε) 275 (5.28), 260 (5.17), 247 (4.87) nm. $[a]_D^{23}$ 149.03 (c 0.21, MeCN). HRMS (ESI): calcd for $C_{36}H_{50}NO_{10}Si$ ([M+NH4]$^+$) 684.3198; found 684.3207. R_f: 0.45 (EtOAc/*n*-heptane 1:1).

15′-Triisopropylsilylundeca-4′,6′,8′,10′,12′,14′-hexaynyl 2,3,4,6-tetra-*O*-acetyl-β-D-gluco–pyranoside TAG6.

Preparation on a 0.5 g Scale. 1-Triisopropylsilyl-6-trimethysilylhexatriyne (577 mg, 1.91 mmol) was dissolved in dry THF (4 mL), cooled to 0 °C, and MeLi·LiBr complex (0.84 mL, 2.2 M in Et_2O, 1.85 mmol)

was added. The cooling bath was removed and the resulting mixture was stirred for 30 min before it was cooled to 0 °C again. $ZnCl_2$ (2.70 mL, 0.7 M in THF, 1.89 mmol) was added, the cooling bath was removed, and the mixture was stirred for 30 min in order to form TIPS-$(C{\equiv}C)_3$-ZnCl **3**. In a second flask, 9'-bromonona-4',6',8'-triynyl 2,3,4,6-tetra-*O*-acetyl-β-D-glucopyranoside **7** (0.50 g, 0.92 mmol) was dissolved in dry toluene (15 mL), and $PdCl_2$(dppf)·DCM (77 mg, 0.09 mmol) was added. The two solutions were combined at 0 °C, the flask was wrapped with aluminum foil, and the resulting mixture was stirred for 6 h at 0 °C and allowed to warm to room temperature over night. $PdCl_2$(dppf)·DCM (38 mg, 0.05 mmol) was added. After 72 h at room temperature, the mixture was diluted with Et_2O, washed once with saturated NH_4Cl solution, and once with saturated NaCl solution. The combined aqueous phases were once extracted with Et_2O, and the combined organic phases were dried over $MgSO_4$ and concentrated *in vacuo*. Column chromatography (silica gel; EtOAc/*n*-heptane 1:2) yielded **TAG6** (0.35 g, 55%) as a brown syrup.

Preparation on a 5 g Scale. 1-Triisopropylsilyl-6-trimethysilylhexatriyne (6.36 g, 21.01 mmol) was dissolved in dry THF (45 mL), cooled to 0 °C, and MeLi·LiBr complex (9.3 mL, 2.2 M in Et_2O, 20.41 mmol) was added. The cooling bath was removed and the mixture was stirred for 30 min before it was cooled again to 0 °C. $ZnCl_2$ (30.02 mL, 0.7 M in THF, 21.01 mmol) was added, the cooling bath was removed, and the mixture was stirred for 30 min in order to form TIPS-$(C{\equiv}C)_3$-ZnCl **3**. In a second flask, 9'-bromonona-4',6',8'-triynyl 2,3,4,6-tetra-*O*-acetyl-β-D-glucopyranoside **7** (6.50 g, 0.92 mmol) was dissolved in dry toluene (15 mL), and $PdCl_2$(dppf)·DCM (196 mg, 0.24 mmol) was added. The two solutions were combined at 0 °C, the flask was wrapped with aluminum foil, and the resulting mixture was stirred for 6 h at 0 °C and allowed to warm to room temperature over night. The mixture was cooled to 0 °C several times over 5 days and $PdCl_2$(dppf)·DCM (791 mg, 0.97 mmol) was added in portions of 200-300 mg. Then, the mixture was diluted with Et_2O, washed once with saturated NH_4Cl solution, and once with saturated NaCl solution. The combined aqueous phases were once extracted with Et_2O, and the combined organic phases were dried over $MgSO_4$ and concentrated *in vacuo*. Column chromatography (silica gel; EtOAc/*n*-heptane 1:2) yielded **TAG6** (3.56 g, 43%) as a brown resin. ^1H NMR (400 MHz, CD_2Cl_2) δ = 5.19 (dd, *J* = 9.6 Hz, 1H, *H3*), 5.02 (dd, *J* = 9.7 Hz, 1H, *H4*), 4.90 (dd, *J* = 9.6, 8.1 Hz, 1H, *H2*), 4.51 (d, *J* = 8.0 Hz, 1H, *H1*), 4.24 (dd, *J* = 12.3, 4.8 Hz, 1H, *H6*), 4.09 (dd, *J* = 12.3, 2.3 Hz, 1H, *H6*), 3.96-3.82 (m, 1H, OC*H*H), 3.72 (ddd, *J* = 9.9, 4.6, 2.3 Hz, 1H, *H5*), 3.60 (ddd, *J* = 9.7, 7.7, 5.2 Hz, 1H, OCH*H*), 2.42 (t, *J* = 6.9 Hz, 2H, $CCCH_2$), 2.04 (s, 3H, CH_3), 2.01 (s, 3H, CH_3), 1.99 (s, 3H, CH_3), 1.96 (s, 3H, CH_3), 1.89–1.73 (m, 2H, $CCCH_2CH_2$), 1.17–1.00 (m, 21H, $Si(CH(CH_3)_2)_3$)). ^{13}C NMR (101 MHz, $CDCl_3$) *δ* = 170.9, 170.5, 169.9, 169.8 (4 *C*=O), 101.4 (*C*1), 89.7, 87.8, 82.1 (3 *C*≡C), 73.2 (*C*3), 72.4 (*C5*), 71.7 (*C2*), 68.9 (*C4*), 68.6 (O*C*H_2), 66.2, 63.5, 63.3, 62.9, 62.8, 62.5 (6 *C*≡C), 62.4 (*C6*), 61.7, 61.5, 60.6 (3 *C*≡C), 28.4 (OCH_2CH_2), 21.0, 21.0, 21.0, 20.9 (4 $COCH_3$), 18.8 ($Si(CH(CH_3)_2)_3$), 16.6 ($CCCH_2$), 11.8 ($Si(CH(CH_3)_2)_3$). IR (ATR) *ν* 2943, 2866, 2208, 2165, 2069, 2037, 1749, 1461, 1427, 1366, 1214, 1169,

1035 (cm^{-1}). UV (MeCN, c = 3.03 µmol/L) λ_{max} (log ε) 296 (5.46), 280 (5.32), 266 (4.98), 252 (4.64) nm. $[a]_D^{23}$ 150.92 (c 0.21, MeCN). HRMS (ESI): calcd for $C_{38}H_{50}NO_{10}Si$ ($[M+NH_4]^+$) 708.3198; found 708.3212. R_f: 0.45 (EtOAc/*n*-heptane 1:1).

4-Tritylphenyl 8-(triisopropylsilyl)octa-5,7-diynoate TPC2. Butyl lithium (2.35 mL, 2.5 M in *n*-hexane, 5.88 mmol) was added to ethynyltriisopropylsilane (1.09 g, 5.99 mmol) in THF (5 mL) at 0 °C, and the resulting mixture was stirred for 10 min. Then $ZnCl_2$ (8.56 mL, 0.7 M in THF, 5.99 mmol) was added at 0 °C, and the resulting mixture was again stirred for 30 min in order to form TIPS-C≡C-ZnCl **1**. In another flask, 4-tritylphenyl 6-bromohex-5-ynoate **9** (1.0 g, 1.96 mmol) and $PdCl_2$(dppf) · DCM (0.16 g, 0.196 mmol) were dissolved in toluene (25 mL). The two solutions were combined at 0 °C and the mixture was stirred for 17 h while warming up to room temperature. After removal of the solvent *in vacuo*, column chromatography (silica gel; DCM/*n*-heptane 1:2) yielded **TPC2** (812 mg, 1.33 mmol, 68%) as a light brown solid. ^1H NMR (400 MHz, CD_2Cl_2): δ = 7.31-7.18 (m, 17H, Ph*H*), 7.04-6.97 (m, 2H, Ph*H*), 2.69 (t, J = 7.3 Hz, 2H, CH_2), 2.46 (t, J = 6.9 Hz, 2H, CH_2), 1.96 (p, J = 7.1 Hz, 2H, CH_2), 1.09 (s, 21H, Si(C*H*(CH_3)$_2$)$_3$). ^{13}C NMR (101 MHz, CD_2Cl_2): δ = 171.9 (*C*OOPhTr), 149.2, 147.3, 145.0, 132.5, 131.5, 128.2, 126.5, 121.1 (24C, *C*-Ar), 90.4, 81.2, 78.1, 67.0 (4C, ≡*C*), 65.2 (*C*Tr), 33.6, 23.8, 19.2 (3C, *C*H_2), 18.9 (Si(CH(*C*H_3)$_2$)$_3$), 11.8 (Si(*C*H(CH_3)$_2$)$_3$). Mp: 84–85°C. IR (ATR) ν 3086, 3057, 3030, 2942, 2891, 2864, 2224, 2104, 1758, 1595, 1492, 1461, 1445, 1366, 1310, 1265, 1205, 1170, 1129, 1073, 1035, 1017 (cm^{-1}). UV (cyclohexane, c = 6.55 µmol/L) λ_{max} (log ε) 203 (4.98) nm. HRMS (MALDI): calcd for $C_{42}H_{46}O_2SiNa$: ($[M+Na]^+$) 633.3157; found: 633.3189. R_f : 0.42 (DCM/*n*-heptane 1:2).

4-Tritylphenyl 10-(triisopropylsilyl)deca-5,7,9-triynoate TPC3. 1-Triisopropylsilylbutadiyne (6.70 g, 32.5 mmol) was dissolved in THF (50 mL), cooled to –78 °C, and butyl lithium (12.6 mL, 2.5 M in *n*-hexane, 31.5 mmol) was added. The cooling bath was removed and the resulting mixture was stirred for 1 h before it was cooled to 0 °C again. $ZnCl_2$ (45.0 mL, 0.7 M in THF, 31.5 mmol) was added, and the resulting mixture was stirred for 30 min at 0°C and then 1 h at room temperature in order to form TIPS-(C≡C)$_2$-ZnCl **2a**. In a second flask, 4-tritylphenyl 6-bromohex-5-ynoate **9** (9.15 g, 18.0 mmol) was dissolved in dry toluene (250 mL), and $PdCl_2$(dppf)·DCM (1.50 g, 1.843 mmol) was added. The two solutions were combined at 0 °C, the flask was wrapped with aluminum foil, and the resulting mixture was stirred for 6 h at 0 °C and allowed to warm to room temperature over night. Then, the mixture was diluted with Et_2O, washed once with saturated NH_4Cl solution, and once with saturated NaCl solution. The organic phase was dried over $MgSO_4$ and concentrated *in vacuo*. Column chromatography (silica gel; DCM/*n*-heptane 1:2) yielded **TPC3** (5.80 g, 51%) as a light brown solid. ^1H NMR (400 MHz, $CDCl_3$): δ = 7.27-7.22 (m, 17H, Ph*H*), 7.01-6.98 (m, 2H, Ph*H*), 2.71 (t, J = 7.3Hz, 2H, CH_2), 2.49 (t, J = 6.8Hz, 2H, CH_2), 1.99 (p, J = 7.1Hz, 2H, CH_2), 1.10 (s, 21H, Si(C*H*(CH_3)$_2$)$_3$). ^{13}C NMR (101 MHz, CD_2Cl_2): δ = 171.3 (*C*OOPhTr), 148.6, 146.7, 144.5, 132.2, 131.2, 127.7, 126.1, 120.4 (24C, *C*-Ar), 90.0, 83.7, 78.9, 66.8 (4C,

≡C), 64.7 (CTr), 61.3, 61.1 (2C, ≡C), 33.0, 23.3, 18.9 (3C, CH$_2$), 18.6 (Si(CH(CH$_3$)$_2$)$_3$), 11.4 (Si(CH(CH$_3$)$_2$)$_3$). IR (ATR) ν 3086, 3057, 3031, 2943, 2891, 2865, 2210, 2164, 2077, 1759, 1595, 1492, 1461, 1445, 1367, 1335, 1308, 1281, 1206, 1170, 1129, 1072, 1035, 1018 (cm^{-1}). Mp: 99–101°C. UV (cyclohexane, c = 6.30 µmol/L) λ_{max} (log ε) 222 (4.97), 212 (5.04) nm. HRMS (MALDI): calcd for $C_{31}H_{26}O_2Na$: 531.0930 ([M+Na]$^+$); found: 531.0923. R$_f$: 0.42 (DCM/n-heptane 1:2).

4-Tritylphenyl 12-(triisopropylsilyl)dodeca-5,7,9,11-tetraynoate TPC4. MeLi · LiBr complex (1.78 mL, 2.2 M in Et$_2$O, 3.93 mmol) was added to 1-triisopropylsilyl-6-trimethylsilylhexatriyne (1.22 g, 4.02 mmol) in THF (10 mL) at 0 °C, and the resulting mixture was stirred for 30 min. Then ZnCl$_2$ (5.75 mL, 0.7 M in THF, 4.02 mmol) was added at 0 °C, and the resulting mixture was again stirred for 30 min in order to form TIPS-(C≡C)$_3$-ZnCl **3**. In another flask, 4-tritylphenyl 6-bromohex-5-ynoate **11** (1.0 g, 1.96 mmol) and PdCl$_2$(dppf) · DCM (0.16 g, 0.196 mmol) were mixed in toluene (40 mL). The two solutions were combined at 0 °C, and the flask was wrapped with aluminum foil. The mixture was stirred for 2 d while warming up to room temperature before the solvent was removed *in vacuo*. Column chromatography (silica gel; DCM/n-heptane 1:2) yielded **TPC4** (0.84 g, 1.27 mmol, 65%) as a light brown solid. ^{1}H NMR (400 MHz, CDCl$_3$): δ = 7.30-7.16 (m, 17H, PhH), 7.03-6.94 (m, 2H, PhH), 2.70 (t, J = 7.3 Hz, 2H, CH_2), 2.50 (t, J = 6.9 Hz, 2H, CH_2), 1.99 (p, J = 7.1 Hz, 2H, CH_2), 1.12-1.07 (m, 21H, Si(CH(CH_3)$_2$)$_3$). ^{13}C NMR (101 MHz, CDCl$_3$): δ = 171.3 (COOPhTr), 148.6, 146.7, 144.5, 132.3, 131.2, 127.7, 126.2, 120.5, 120.4 (24C, C-Ar), 89.8, 85.2, 79.4, 66.9 (4C, ≡C), 64.7 (CTr), 62.2, 61.6, 61.0 (4C, ≡C), 33.0, 23.2, 19.0 (3C, CH$_2$), 18.7 (Si(CH(CH$_3$)$_2$)$_3$), 11.4 (Si(CH(CH$_3$)$_2$)$_3$). IR (ATR) ν 3086, 3057, 3030, 2943, 2891, 2865, 2211, 2138, 2058, 1757, 1595, 1492, 1461, 1444, 1366, 1309, 1205, 1170, 1130, 1034, 1017 (cm^{-1}). UV (cyclohexane, c = 6.07 µmol/L) λ_{max} (log ε) 252 (4.86), 240 (4.77) nm. HRMS (APPI): calcd for $C_{46}H_{46}O_2Si$: 658.3267 ([M]$^+$); found: 658.3267. R$_f$: 0.43 (DCM/n-heptane 1:1).

4-Tritylphenyl 14-(triisopropylsilyl)tetradeca-5,7,9,11,13-pentaynoate TPC5.

(i) 4-Tritylphenyl 10-(trimethylsilyl)deca-5,7,9-triynoate **10** (0.30 g, 0.55 mmol) was dissolved in dry MeCN (8 mL) and dry DCM (10 mL). The flask was shielded from light with aluminum foil, and N-bromosuccinimide (102 mg, 0.57 mmol) as well as AgF (73 mg, 0.57 mmol) were added. The resulting mixture was stirred for 4 h after which it was diluted with DCM, washed five times with 1 M HCl, and once with saturated NaCl solution. The organic phase was dried over Na$_2$SO$_4$, and concentrated *in vacuo* to approximately 10 mL while thoroughly shielding it from light. Toluene was added (10 mL), the mixture was concentrated *in vacuo*, and this solution containing the bromotriacetylene **11** was used without further purification in the next step.

(ii) MeLi · LiBr complex (0.74 mL, 2.2 M in Et$_2$O, 1.64 mmol) was added to 1-triisopropylsilyl-4-trimethylsilylbutadiyne (463 mg, 1.66 mmol) in THF (4 mL) at 0 °C, and the

resulting mixture was stirred for 40 min. Then $ZnCl_2$ (2.37 mL, 0.7 M in THF, 1.66 mmol) was added at 0 °C, and the resulting mixture was stirred for additional 3 h in order to form TIPS-(C≡C)$_2$-ZnCl **2a**. In a second flask, $PdCl_2$(dppf) · DCM (44.5 mg, 0.36 mmol) was added to toluene (70 mL) at 0 °C. The toluene solution containing the 4-tritylphenyl 10-bromodeca-5,7,9-triynoate **11** (10 mL, 0.55 mmol) and the zinc acetylide solution were simultaneously added at this temperature, and the flask was shielded from light with aluminum foil. The mixture was stirred for 2 d while slowly warming to room temperature before the solvent was removed *in vacuo*. Column chromatography (silica gel; DCM/*n*-heptane 1:2) furnished the 4-tritylphenyl 14-(triisopropylsilyl)tetradeca-5,7,9,11,13-pentaynoate **TPC5** (218 mg, 0.32 mmol, 59%) as a brown solid. ^1H NMR (400 MHz, $CDCl_3$): δ = 7.32-7.22 (m, 17H, Ph*H*), 7.05-7.01 (m, 2H, Ph*H*), 2.73 (t, *J* = 7.2 Hz, 2H, CH_2), 2.53 (t, *J* = 6.8 Hz, 2H, CH_2), 2.03 (p, *J* = 7.1 Hz, 2H, CH_2), 1.15-1.08 (m, 21H, Si(C*H*(CH_3)$_2$)$_3$). ^{13}C NMR (101 MHz, $CDCl_3$): δ = 171.2 (*C*OOPhTr), 148.6, 146.7, 144.5, 132.2, 131.2, 127.7, 126.1, 120.4 (24C, *C*-Ar), 89.7, 86.3, 80.0, 66.8 (4C, ≡*C*), 64.7 (*C*Tr), 62.7, 62.5, 62.2, 61.8, 61.6, 61.1 (8C, ≡*C*), 33.0, 23.1, 19.0 (3C, *C*H_2), 18.6 (Si(CH(*C*H_3)$_2$)$_3$), 11.4 (Si(*C*H(CH_3)$_2$)$_3$). IR (ATR) ν 3086, 3057, 3030, 2943, 2890, 2865, 2211, 2173, 2102, 2047, 1756, 1595, 1492, 1461, 1444, 1367, 1311, 1264, 1205, 1170, 1130, 1071, 1017 (cm^{-1}). UV (cyclohexane, c = 5.86 µmol/L) λ_{max} (log ε) 277 (5.29), 262 (5.16), 249 (4.84) nm. HRMS (APPI): calcd for $C_{48}H_{46}O_2Si$: 682.3267 ($[M]^+$); found: 682.3264. R_f : 0.54 (DCM/n-heptane 1:1).

4-Tritylphenyl 16-(triisopropylsilyl)hexadeca-5,7,9,11,13,15-hexaynoate TPC6.

(i) 4-Tritylphenyl 10-(trimethylsilyl)deca-5,7,9-triynoate **10** (2.00 g, 3.63 mmol) was dissolved in dry MeCN (18 mL) and dry DCM (18 mL). The flask was shielded from light with aluminum foil, and *N*-bromosuccinimide (679 mg, 3.81 mmol) as well as AgF (484 mg, 3.81 mmol) were added. The resulting mixture was stirred for 28 h after which it was diluted with DCM, washed four times with 1 M HCl, and once with saturated NaCl solution. The organic phase was dried over Na_2SO_4, and concentrated *in vacuo* to approximately 10 mL while thoroughly shielding it from light. Toluene was added (15 mL), the mixture was concentrated *in vacuo*, and this solution containing the bromotriacetylene **11** was used without further purification in the next step.

(ii) MeLi · LiBr complex (3.30 mL, 2.2 M in Et_2O, 7.26 mmol) was added to 1-triisopropylsilyl-6-trimethylsilylhexatriyne (2.25 g, 7.44 mmol) in THF (15 mL) at 0 °C, and the resulting mixture was stirred for 40 min. Then $ZnCl_2$ (10.63 mL, 0.7 M in THF, 7.44 mmol) was added at 0 °C, and the resulting mixture was stirred for additional 45 min in order to form TIPS-(C≡C)$_3$-ZnCl **3**. In a second flask, $PdCl_2$(dppf) · DCM (296 mg, 0.36 mmol) was added to toluene (150 mL) at 0 °C. The toluene solution containing the 4-tritylphenyl 10-bromodeca-5,7,9-triynoate **11** (10 mL, 3.63 mmol) and the zinc acetylide solution were simultaneously added at this temperature, and the flask was shielded from light with aluminum foil. After 24 h, the mixture was diluted with Et_2O, washed two times with

saturated NH_4Cl solution, and once with saturated NaCl solution. The organic phase was dried over Na_2SO_4, and concentrated *in vacuo*. Column chromatography (silica gel; DCM/*n*-heptane 1:2) yielded 4-tritylphenyl 16-(triisopropylsilyl)hexadeca-5,7,9,11,13,15-hexaynoate **TPC6** (1.13 g, 1.60 mmol, 44%) as a brown solid. ^{1}H NMR (400 MHz, CD_2Cl_2): δ = 7.29-7.18 (m, 17H, Ph*H*), 7.00-6.96 (m, 2H, Ph*H*), 2.67 (t, *J* = 7.3 Hz, 2H, CH_2), 2.51 (t, *J* = 6.9 Hz, 2H, CH_2), 1.97 (p, *J* = 7.1 Hz, 2H, CH_2), 1.11-1.07 (m, 21H, $Si(CH(CH_3)_2)_3$). ^{13}C NMR (101 MHz, CD_2Cl_2): δ = 171.6 (*C*OOPhTr), 149.2, 147.3, 147.3, 145.1, 132.5, 131.5, 128.2, 126.5, 121.0 (24C, *C*-Ar), 89.7, 87.9, 81.6, 66.6 (4C, ≡*C*), 65.2 (*C*Tr), 63.4, 63.2, 62.9, 62.7, 62.6, 61.8, 61.4, 60.8 (8C, ≡*C*), 33.4, 23.5, 19.5 (3C, CH_2), 18.8 ($Si(CH(CH_3)_2)_3$), 11.8 ($Si(CH(CH_3)_2)_3$). IR (ATR) ν 3086, 3057, 3030, 2943, 2891, 2865, 2209, 2166, 2069, 2038, 1759, 1595, 1493, 1461, 1445, 1367, 1325, 1206, 1170, 1131, 1073, 1018 (cm^{-1}). UV (cyclohexane, *c* = 5.66 µmol/L) λ_{max} (log ε) 299 (5.47), 282 (5.33), 267 (4.98) 254 (4.58) nm. HRMS (ESI): calcd for $C_{50}H_{50}NO_2Si$: 724.3605 ($[M+NH_4]^+$); found: 724.3616. R_f : 0.45 (DCM/n-heptane 1:1).

15'-Triisopropylsilylpentadeca-4',6',8',10',12',14'-hexynyl β-D-glucopyranoside GLU6-TIPS. In a flask shielded from light with aluminum foil, 15'-triisopropylsilylpentadeca-4',6',8',10',12',14'-hexynyl 2,3,4,6-tetra-*O*-acetyl-β-D-glucopyranoside **TAG6** (10.5 mg, 0.015 mmol) was dissolved in 1,4-dioxane (2 mL). MeOH (0.4 mL). NaOMe (1 mg, 0.017 mmol) was added, and the resulting mixture was stirred for 2 h. Then, Amberlite IR-120 (H^+) was added until the solution was neutralized, and the resulting mixture was stirred for 30 min. The solution was filtered from the Amberlite and transferred into a brown glass vial using a syringe with a fine needle. The crude compound was purified by column chromatography (silica gel; EtOAc/MeOH 4:1). Removal of the solvent by freeze-drying yielded **GLU6-TIPS** as a yellow oil that was immediately redissolved in dioxane/MeOH and stored in the refrigerator. ^{1}H NMR (400 MHz, DMSO) δ = 5.01 (d, *J* = 4.8 Hz, 1H, O*H*), 4.93 (d, *J* = 4.8 Hz, 1H, O*H*), 4.89 (d, *J* = 5.0 Hz, 1H, O*H*), 4.47 (dd, *J* = 5.9 Hz, 1H, O*H*), 4.09 (d, *J* = 7.8 Hz, 1H, *H1*), 3.78 (dt, *J* = 6.0 Hz, 10.2 Hz, 1H, OC*H*H), 3.65 (ddd, *J* = 1.9 Hz, 5.6 Hz, 11.6 Hz, 1H, *H6*), 3.54–3.37 (m, 2H, OCH*H* and *H6*), 3.15–2.98 (m, 3H, *H3*, *H4*, *H5*), 2.92 (ddd, *J* = 4.8 Hz, 7.9 Hz, 8.9 Hz, 1H, *H2*), 2.59 (t, *J* = 7.1 Hz, 2H, CCCH_2), 1.83–1.71 (m, 2H, CCCH_2CH_2), 1.09–1.01 (m, 21H, $Si(CH(CH_3)_2)_3$). ^{13}C NMR (101 MHz, DMSO) δ = 102.8, 88.6, 87.9, 84.7, 76.8, 76.6, 73.4, 70.0, 66.9, 64.5, 63.7, 63.0, 62.3, 62.1, 61.7, 61.0, 60.9, 60.7, 59.3, 27.6, 18.3, 15.6, 10.6. IR (ATR) ν 3337, 2944, 2925, 2889, 2866, 2244, 2209, 2165, 2088, 2068, 2039, 1717, 1648, 1597, 1460, 1366, 1250, 1207, 1159, 1072, 1032, 1017 (cm^{-1}). HRMS (ESI): calcd for $C_{30}H_{38}NaO_6Si$ ($[M+Na]^+$) 545.2330; found 545.2333. R_f: 0.09 (EtOAc).

Methyl 16-(triisopropylsilyl)hexadeca-5,7,9,11,13,15-hexaynoate MEC6-TIPS. In a flask shielded from light with aluminum foil 4-tritylphenyl-16-(triisopropylsilyl)hexadeca-5,7,9,11,13,15-hexaynoate **TPC6** (240 mg, 0.340 mmol) was dissolved in DCM (5 mL) and MeOH (1 mL). NaOMe (45 mg, 0.833 mmol) was added, and the resulting mixture was stirred for 3 h. NaOMe (20 mg) was

added, and the mixture was again stirred for 2 h. Then, Amberlite IR-120 (H^+) was added until the solution was neutralized, and the resulting mixture was stirred for 1 h. The Amberlite was filtered off, and the solution was transferred into a brown glass vial using a syringe with a fine needle. The crude compound was purified by column chromatography (silica gel; DCM). Removal of the solvent by freeze-drying yielded **MEC6-TIPS** as a yellow oil that was immediately redissolved in dioxane/MeOH and stored in the refrigerator. ^{1}H NMR (400 MHz, $CDCl_3$) δ = 3.68 (s, 3H, C*H₃*), 2.43 (dt, *J* = 7.0 Hz, 4.8 Hz, 4H, 2 CH_2), 1.88 (p, *J* = 7.1 Hz, 2H, CH_2), 1.09–1.01 (m, 21H, Si(C*H*(CH_3)$_2$)$_3$). ^{13}C NMR (101 MHz, $CDCl_3$) δ = 173.5 (*C*OOCH$_3$), 89.9, 87.3, 81.0, 69.9, 63.3, 63.2, 62.9, 62.9, 62.7, 62.1, 61.8, 61.2 (12C, ≡*C*), 52.2 (COO*C*H$_3$), 33.0, 23.5, 19.5 (3C, *C*H$_2$), 19.0 (Si(CH(*C*H$_3$)$_2$)$_3$), 11.7 (Si(*C*H(CH$_3$)$_2$)$_3$). IR (ATR) ν 2944, 2894, 2866, 2209, 2173, 2081, 2038, 1739, 1460, 1435, 1420, 1364, 1317, 1256, 1206, 1153, 1071, 1017, 1000 (cm^{-1}). R_f: 0.83 (DCM).

Octadeca-4,6,8,10,12,14-hexayne-1,18-diyl bis(2,3,4,6-tetra-*O*-acetyl-β-D-glucopyranoside) TAG6TAG. In a flask shielded from light with aluminum foil, 9'-trimethylsilylnona-4',6',8'-triynyl 2,3,4,6-tetra-*O*-acetyl-β-D-glucopyranoside **6** (1.00 g, 1.88 mmol) was dissolved in THF (20 mL) and water (10 mL). CsF (370 mg, 2.44 mmol) was added, and the resulting mixture was stirred for 2 h. The mixture was diluted with water (50 mL), and then extracted with ethyl acetate (three times 100 mL). The combined organic phases were dried over Na_2SO_4, concentrated *in vacuo* to a volume of 10 mL, and filtered through a plug of silica using DCM as the eluent. The combined fractions were concentrated *in vacuo* to a volume of 50 mL, $Cu(OAc)_2 \cdot H_2O$ (1.88 g, 9.40 mmol) and 2,6-dimethylpyridine (6.6 mL, 56.40 mmol) were added, and the resulting mixture was stirred for 24 h. To this mixture 1 M HCl (100 mL) was added and the aqueous layer was extracted with DCM. The combined organic phases were washed with saturated NaCl solution, dried over Na_2SO_4, and concentrated *in vacuo*. Column chromatography (silica gel; DCM → DCM/acetone 10:1) yielded **TAG6TAG** (630 mg, 73%) as a light brown solid. ^{1}H NMR (400 MHz, $CDCl_3$): δ = 5.20 (dd, *J* = 9.5 Hz, 1H, *H3*), 5.07 (dd, *J* = 9.7 Hz, 1H, *H4*), 4.97 (dd, *J* = 9.6, 8.1 Hz, 1H, *H2*), 4.49 (d, *J* = 8.0 Hz, 1H, *H1*), 4.26 (dd, *J* = 12.3, 4.7 Hz, 1H, *H6*), 4.13 (dd, *J* = 12.3, 2.2 Hz, 1H, *H6*), 3.93 (dt, *J* = 10.2, 5.3 Hz, 1H, OC*H*H), 3.75-3.64 (m, 1H, *H5*), 3.65-3.50 (m, 1H, OCH*H*), 2.41 (t, *J* = 6.7 Hz, 2H, CCCH_2), 2.09 (s, 3H, CH_3), 2.05 (s, 3H, CH_3), 2.02 (s, 3H, CH_3), 2.00 (s, 3H, CH_3), 1.95 – 1.70 (m, 2H, CCCH$_2$CH_2). ^{13}C NMR (101 MHz, $CDCl_3$): δ = 170.8, 170.4, 169.6, 169.5 (4 *C*=O), 101.1 (*C*1), 80.7(*C*≡C), 72.9 (*C*3), 72.0 (*C5*), 71.4 (*C2*), 68.5 (*C4*), 68.1 (O*C*H$_2$), 66.2 (*C*≡C), 62.6, 62.5 (2 *C*≡C), 62.0 (*C6*), 61.8, 60.7 (2 *C*≡C), 27.9 (OCH$_2$*C*H$_2$), 20.9, 20.8, 20.8, 20.7 (4 CO*C*H$_3$), 16.1 (CC*C*H$_2$). HRMS (APPI): calcd for $C_{46}H_{50}O_{20}$: 922.2895 ([M]$^+$); found: 922.2892. R_f: 0.76 (DCM/Acetone 10:1).

Octadeca-4,6,8,10,12,14-hexayne-1,18-diyl bis(β-D-glucopyranoside) GLU6GLU. Octadeca-4',6',8',10',12',14'-hexayne-1',18'-diyl bis(2,3,4,6-tetra-*O*-acetyl-β-D-glucopyranoside)

TAG6TAG (100 mg, 0.108 mmol) was dissolved in 1,4-dioxane (20 mL), and MeOH (4 mL). NaOMe (15 mg, 0.245 mmol) was added, and the resulting mixture was stirred for 3 h. Then, Amberlite IR-120 (H^+) was added until the solution was neutralized, and the resulting mixture was stirred for 30 min. The solution was filtered from the Amberlite and transferred into a brown glass vial using a syringe with a fine needle. The crude compound was purified by column chromatography (silica gel; EtOAc/MeOH 3:1). Removal of the solvent by freeze-drying yielded **GLU6GLU** as a yellow solid that was immediately redissolved in dioxane/MeOH and stored in the refrigerator. ^1H NMR (400 MHz, MeOD) δ = 4.25 (d, J = 7.4 Hz, 1H), 4.00-3.91 (m, 1H), 3.85 (d, J = 11.8 Hz, 1H), 3.71–3.58 (m, 2H), 3.40-3.24 (m, 3H), 3.16 (t, J = 8.2 Hz, 1H), 2.55 (t, J = 6.5 Hz, 2H), 1.92– 1.81 (m, 2H). ^{13}C NMR (101 MHz, MeOD) δ = 104.4, 83.4, 78.0, 77.9, 75.1, 71.6, 69.0, 65.8, 63.7, 62.9, 62.7, 61.7, 60.3, 29.3, 16.8. R_f: 0.26 (EtOAc/MeOH 3:1).

(*T,T*)-[*3*]{[Octadeca-4,6,8,10,12,14-hexayne-1,18-diyl bis(β-D-glucopyranoside)]-*rotaxa*-[*2*] [α-cyclodextrin]} GLU6GLU · 2 CD. In a flask shielded from light with aluminum foil, 9’-trimethylsilylnona-4’,6’,8’-triynyl 2,3,4,6-tetra-*O*-acetyl-β-D-glucopyranoside **6** (70 mg, 0.131 mmol) was dissolved in diethyl ether (1 mL) and MeOH (0.2 mL). NaOMe (2.2 mg, 0.04 mmol) was added, and the resulting mixture was stirred for 2 h. Then, Amberlite IR-120 (H^+) was added until the solution was neutralized, and the resulting mixture was stirred for 1 h. The Amberlite was filtered off, and the solution was transferred into a brown glass vial using a syringe with a fine needle. MeOH (0.3 mL) was added to rinse the flask. An aqueous solution of α-cyclodextrin (0.1 mol/L, 6.9 mL) was added to this solution, and the resulting mixture was heated to 45 °C for 3 h under a slight argon stream in order to remove the diethyl ether. The mixture was then cooled to room temperature, $CuBr_2$ (79 mg, 0.354 mmol) and TMEDA (41 mg, 0.354 mmol) were added, and the reaction mixture was stirred for 2 d. A precipitate formed that was filtered off and purified by column chromatography on a Sephadex G25 column (water) and subsequently by reverse-phase HPLC. [3]Rotaxane **GLU6GLU · 2 CD** was obtained as a slightly yellow solid (68 mg, 21%). ^1H NMR (400 MHz, D_2O) δ = 5.11 (d, J = 3.3 Hz, 12H, H CD), 4.50 (d, J = 7.9 Hz, 2H), 4.11-3.64 (m, 72H CD + 8H), 3.59-3.46 (m, 4H), 3.45-3.37 (m, 2H), 3.31 (dd, J = 9.3 Hz, 8.0 Hz, 2H), 2.76-2.68 (m, 4H), 2.00 (p, J = 6.3 Hz, 4H). ^{13}C NMR (101 MHz, D_2O) δ = 102.3 (CD), 101.7, 84.3, 80.5 (CD), 75.8, 75.6, 73.8 (CD), 73.2, 72.0(CD), 71.7 (CD), 69.6, 68.3, 64.2, 63.3, 61.1, 60.7, 59.6 (CD), 58.0, 27.9, 15.7. MS (MALDI): calcd for $C_{102}H_{154}NaO_{72}$ ($[M+Na]^+$) 2553.8; found 2553.2.

3. NMR Spectra

^{1}H NMR spectrum (CD_2Cl_2, 400 MHz) of **4**

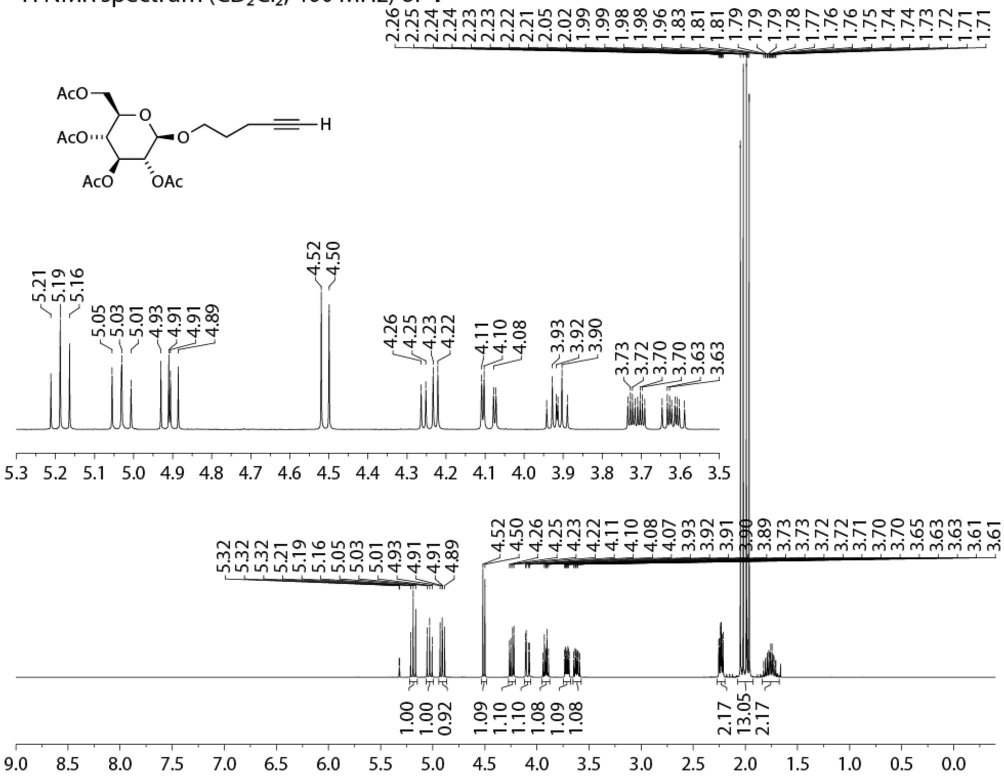

^{13}C NMR spectrum (CD_2Cl_2, 101 MHz) of **4**

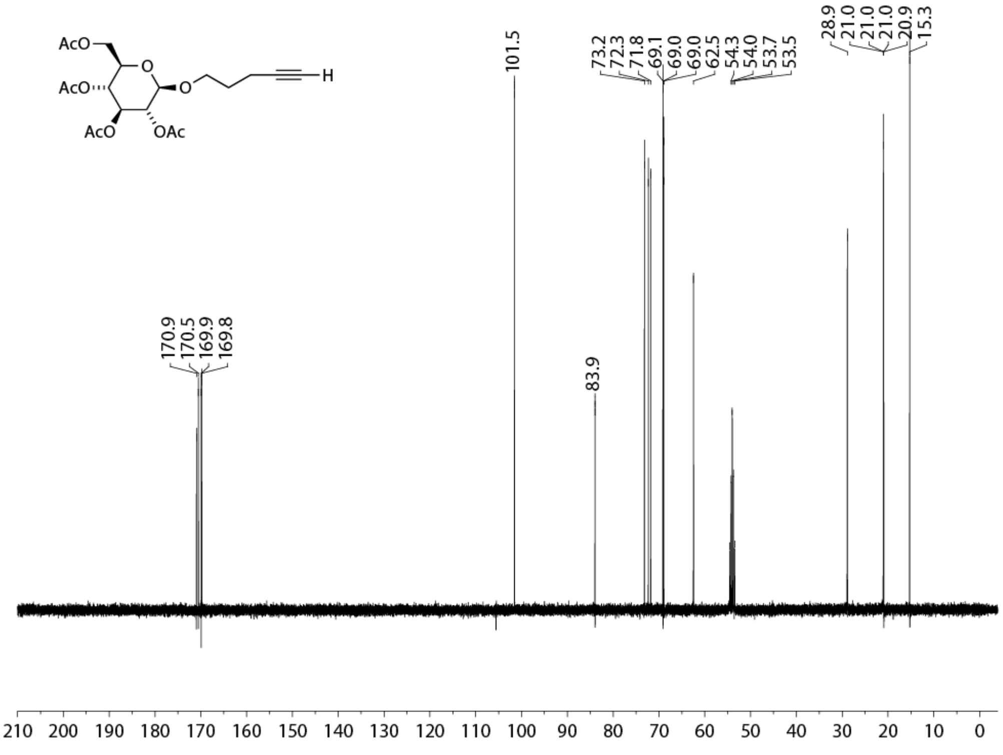

^{1}H NMR spectrum (CD_2Cl_2, 400 MHz) of **5**

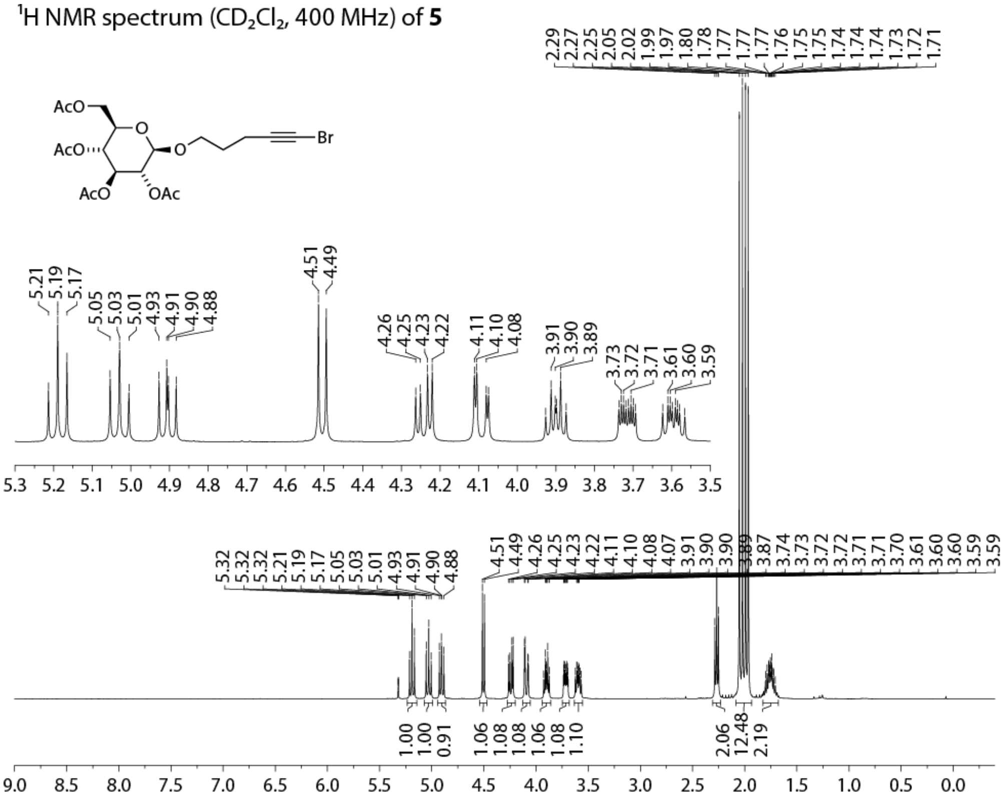

^{13}C NMR spectrum (CD_2Cl_2, 101 MHz) of **5**

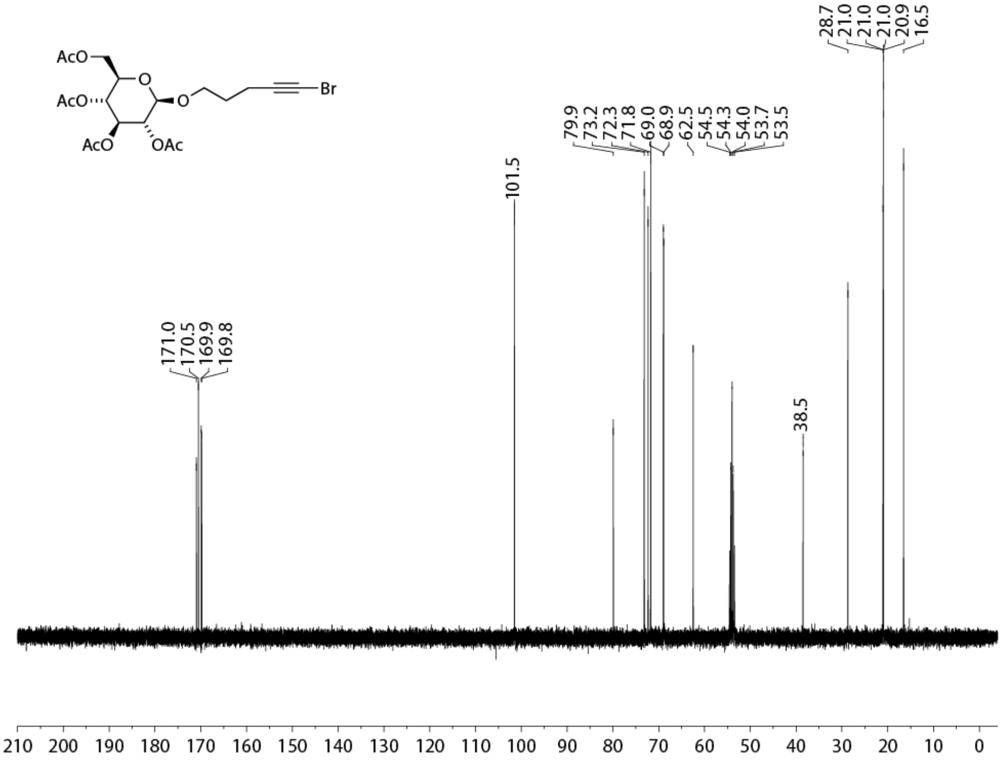

^{1}H NMR spectrum (CD_2Cl_2, 400 MHz) of **6**

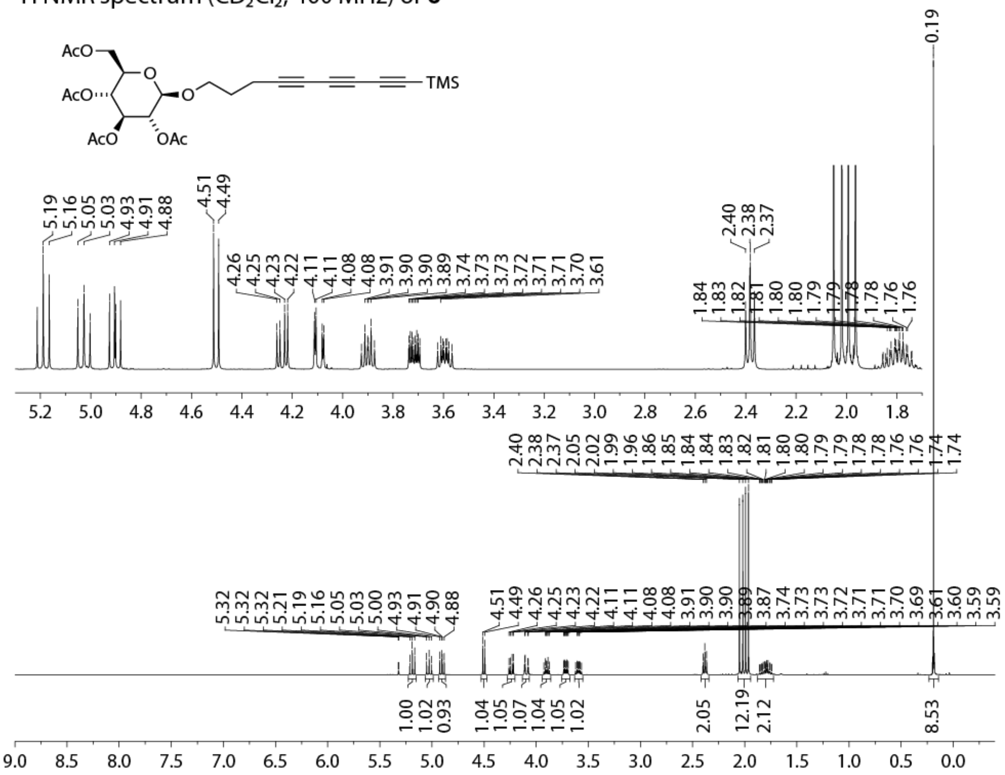

^{13}C NMR spectrum (CD_2Cl_2, 101 MHz) of **6**

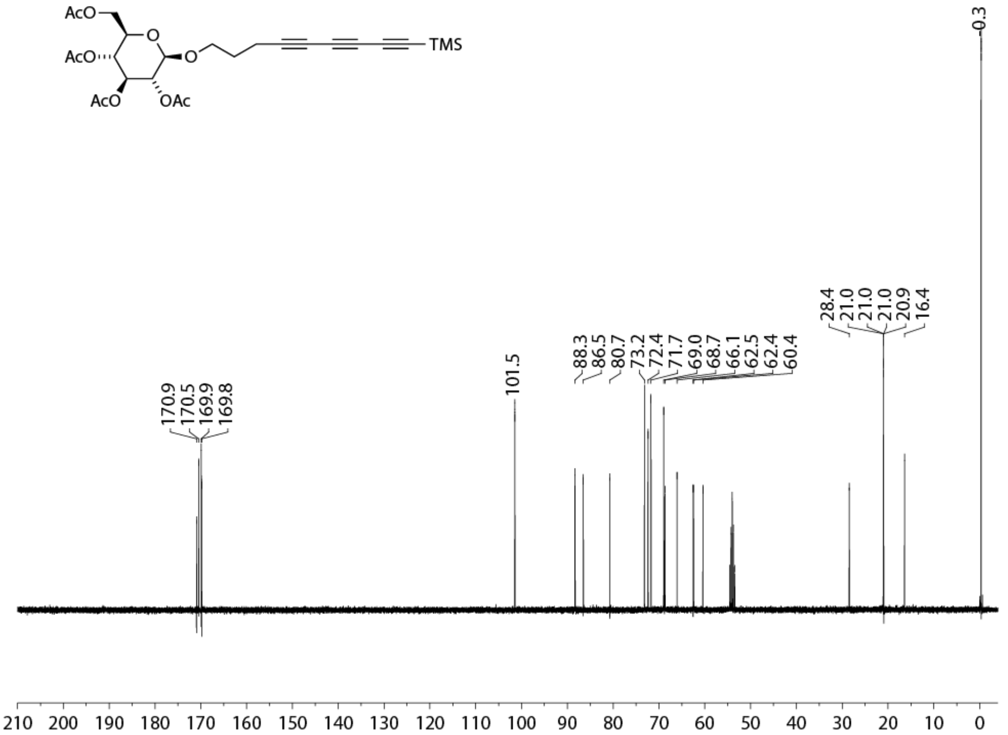

^{1}H NMR spectrum (CD_2Cl_2, 400 MHz) of **7**

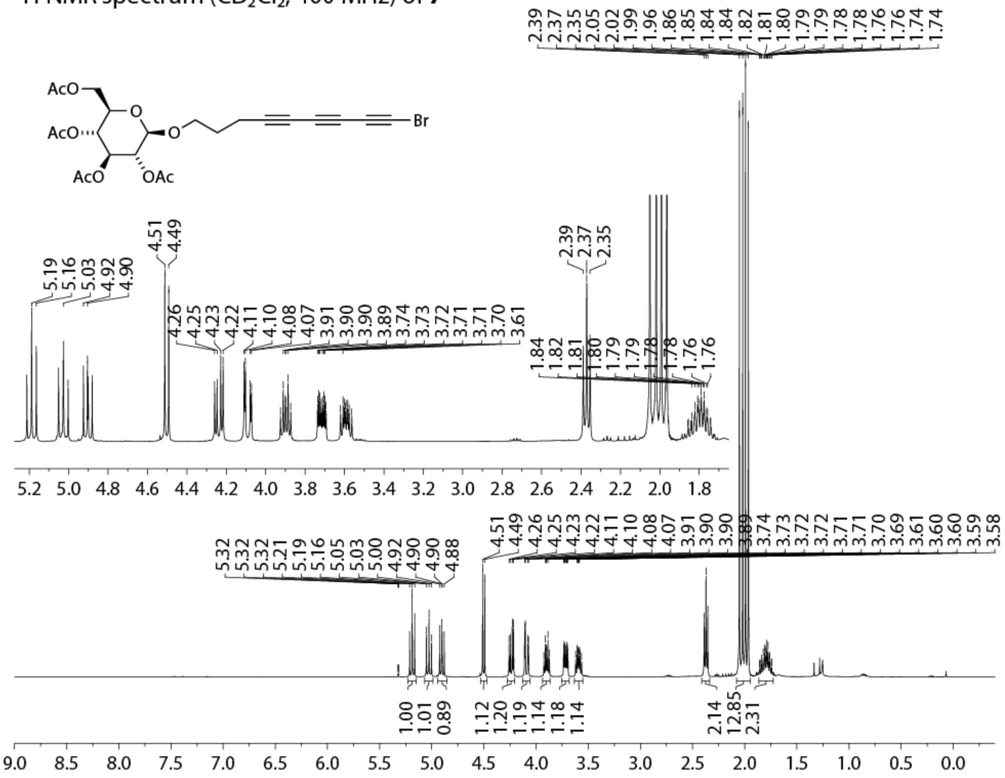

^{13}C NMR spectrum (CD_2Cl_2, 101 MHz) of **7**

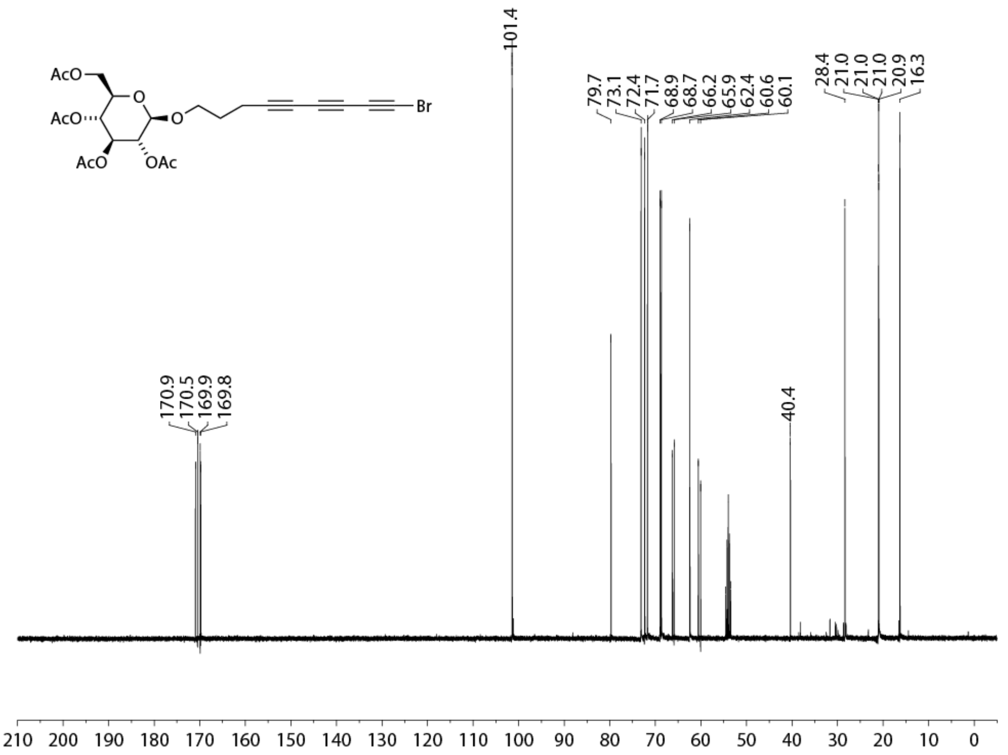

^{1}H NMR spectrum ($CDCl_3$, 400 MHz) of **8**

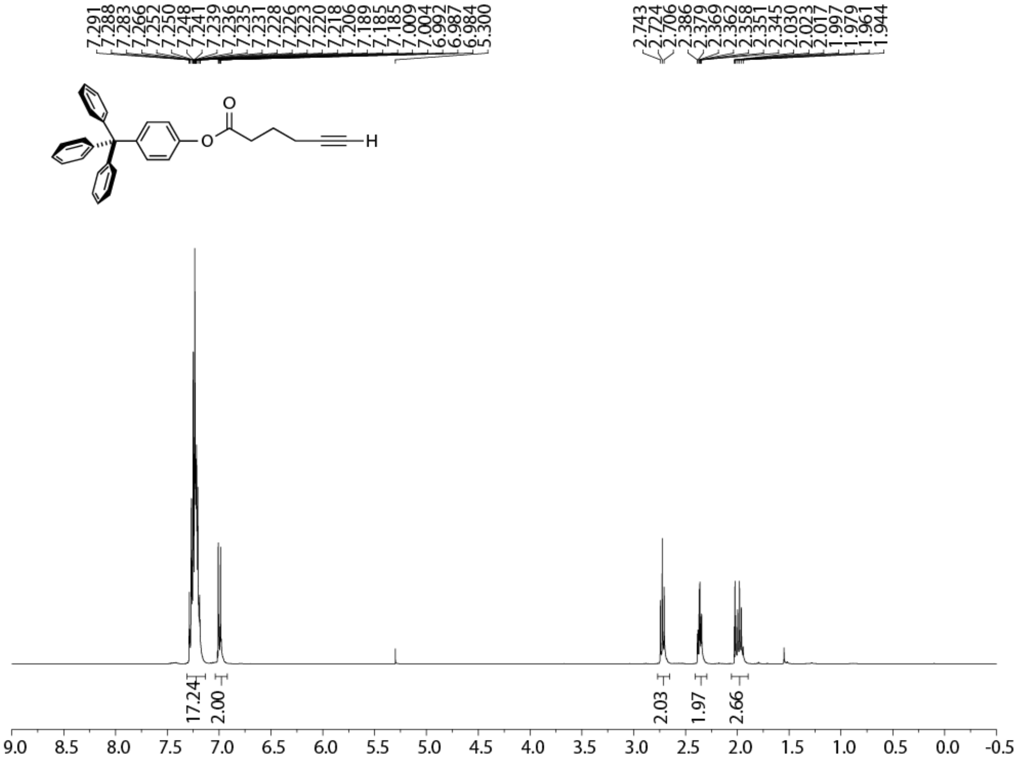

^{13}C NMR spectrum ($CDCl_3$, 101 MHz) of **8**

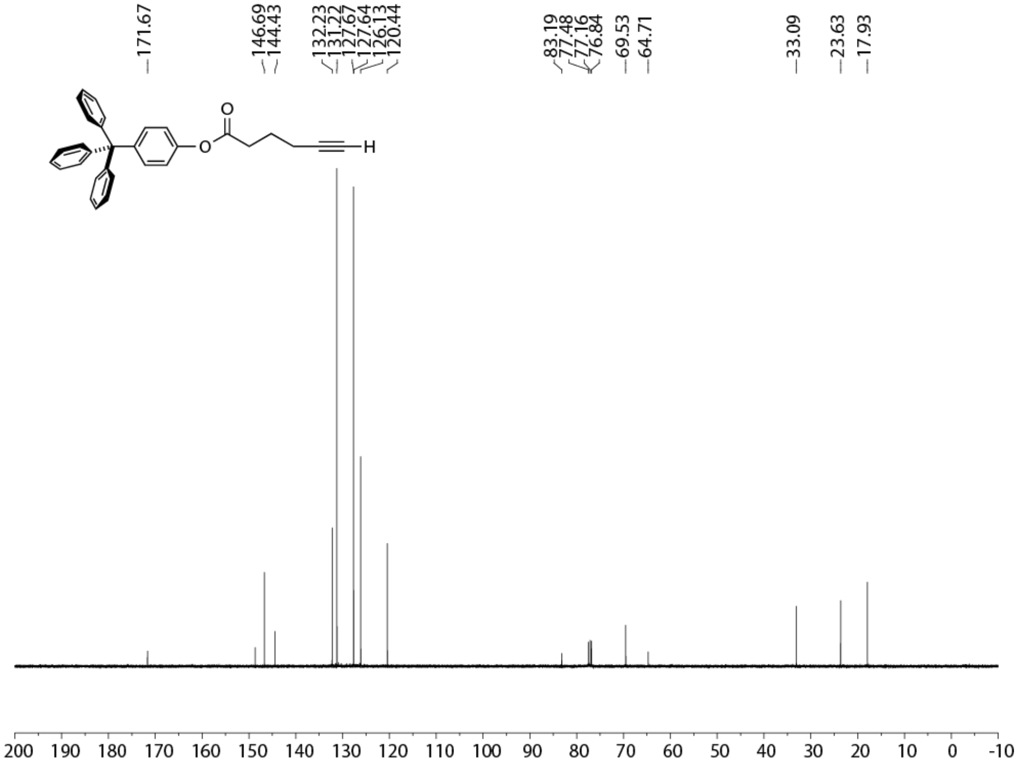

^{1}H NMR spectrum ($CDCl_3$, 400 MHz) of **9**

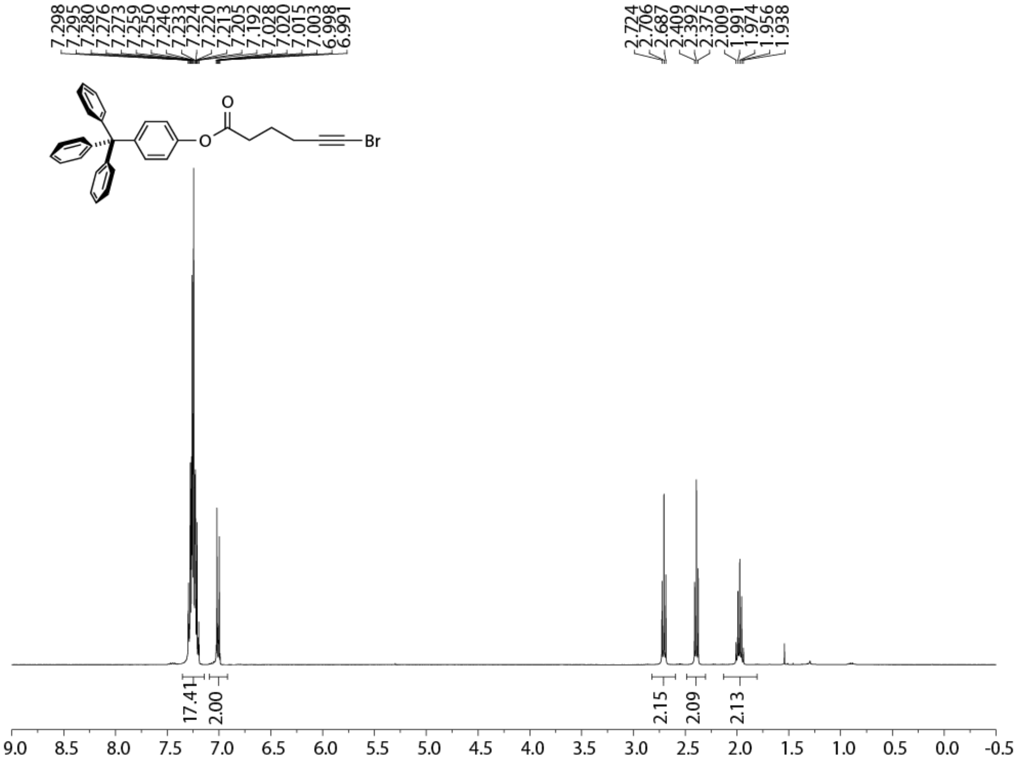

^{13}C NMR spectrum ($CDCl_3$, 101 MHz) of **9**

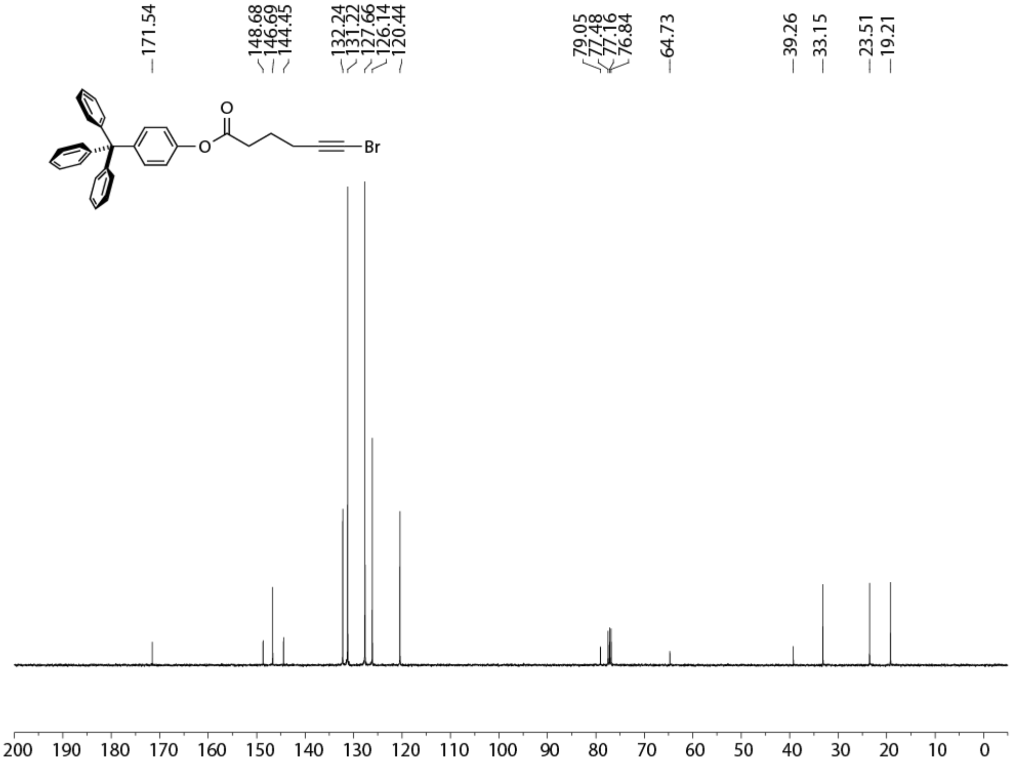

^{1}H NMR spectrum ($CDCl_3$, 400 MHz) of **10**

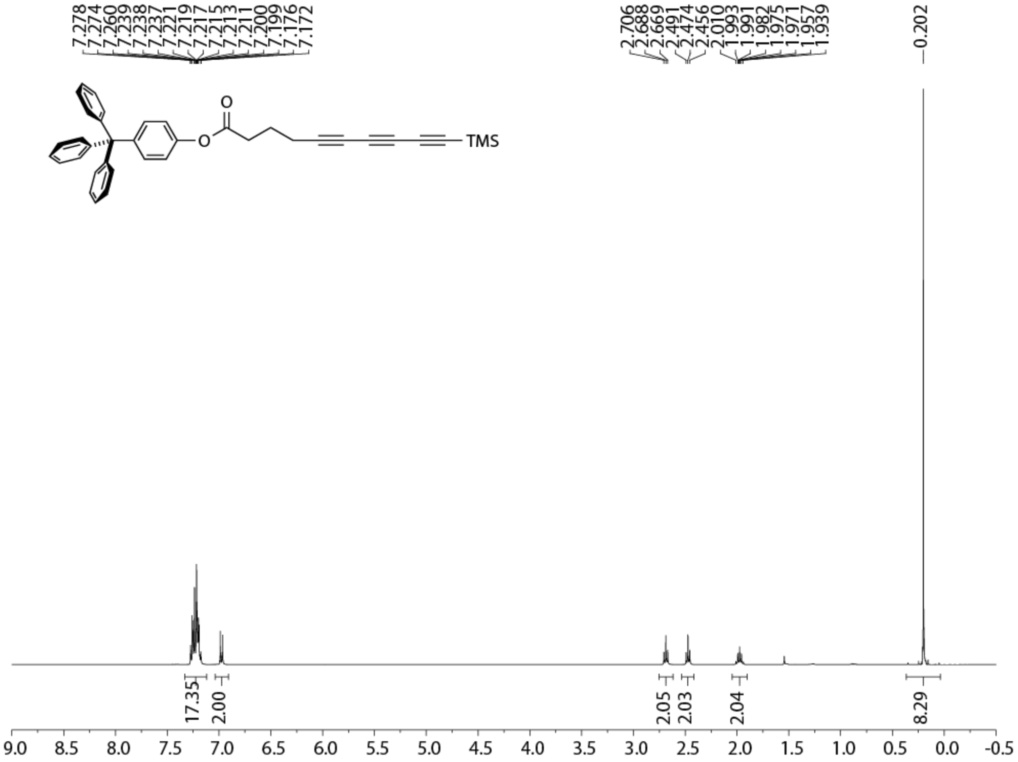

^{13}C NMR spectrum ($CDCl_3$, 101 MHz) of **10**

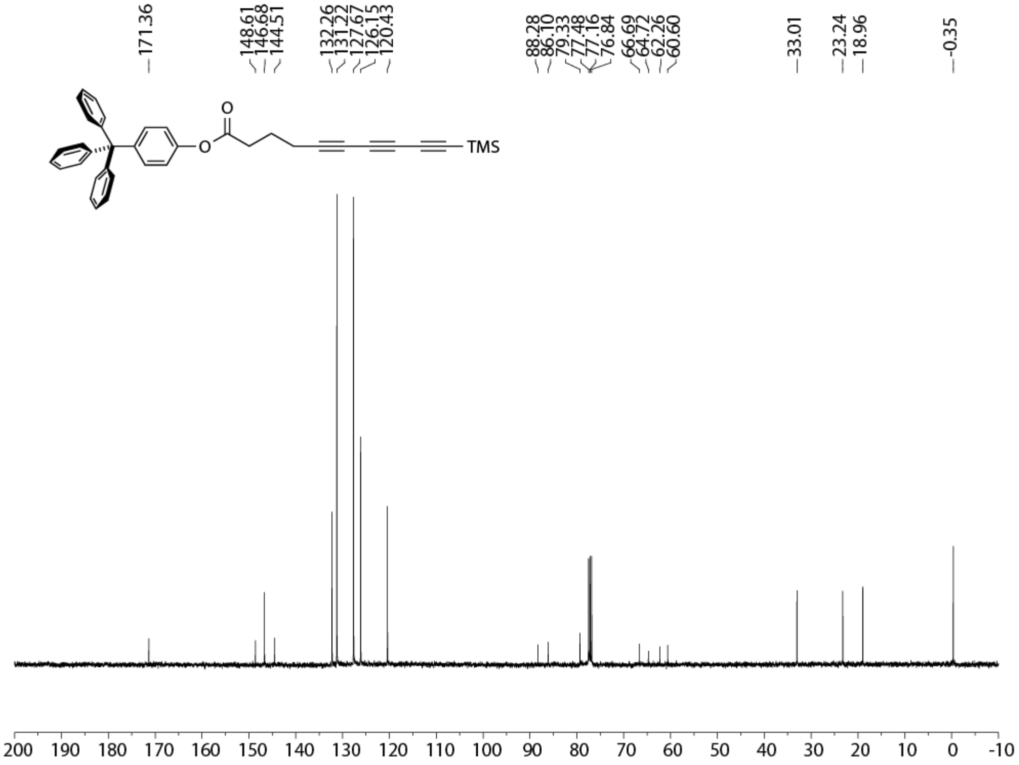

^{1}H NMR spectrum ($CDCl_3$, 400 MHz) of **11**

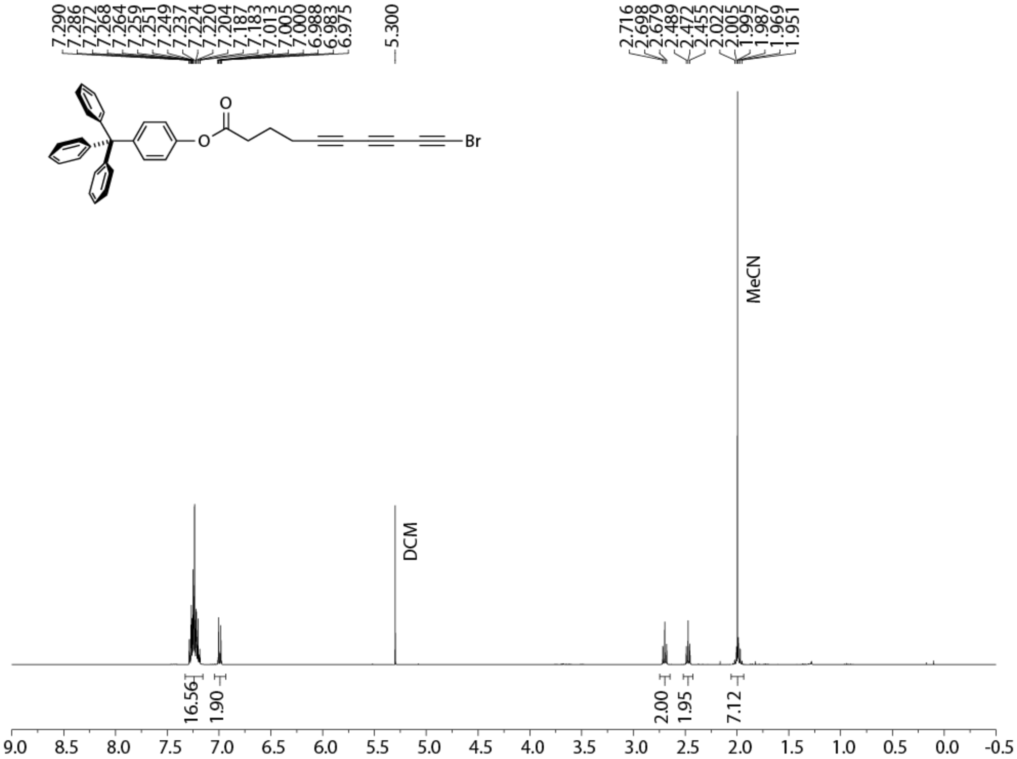

^{13}C NMR spectrum ($CDCl_3$, 101 MHz) of **11**

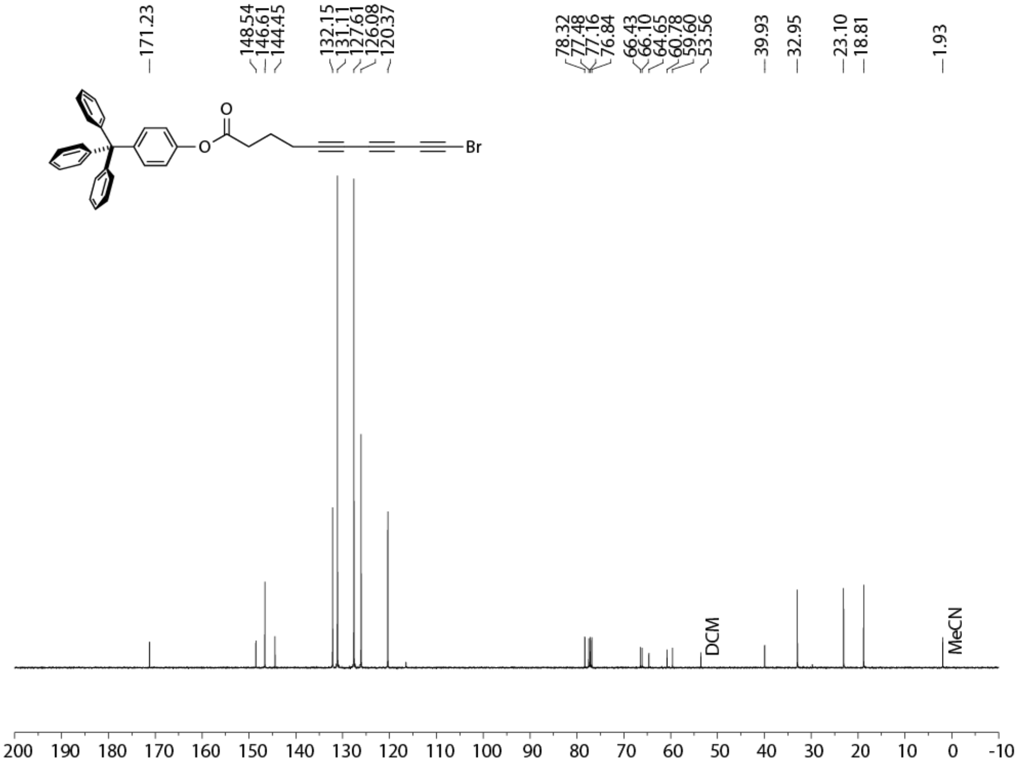

^{1}H NMR spectrum (CD_2Cl_2, 400 MHz) of **TAG2**

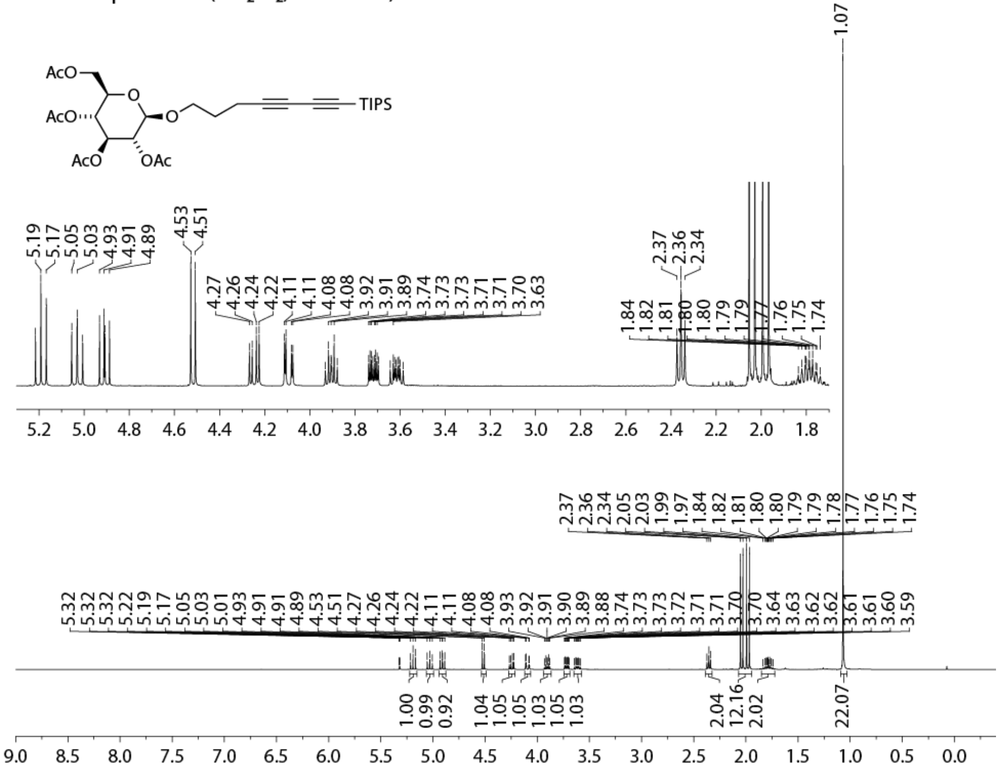

^{13}C NMR spectrum (CD_2Cl_2, 101 MHz) of **TAG2**

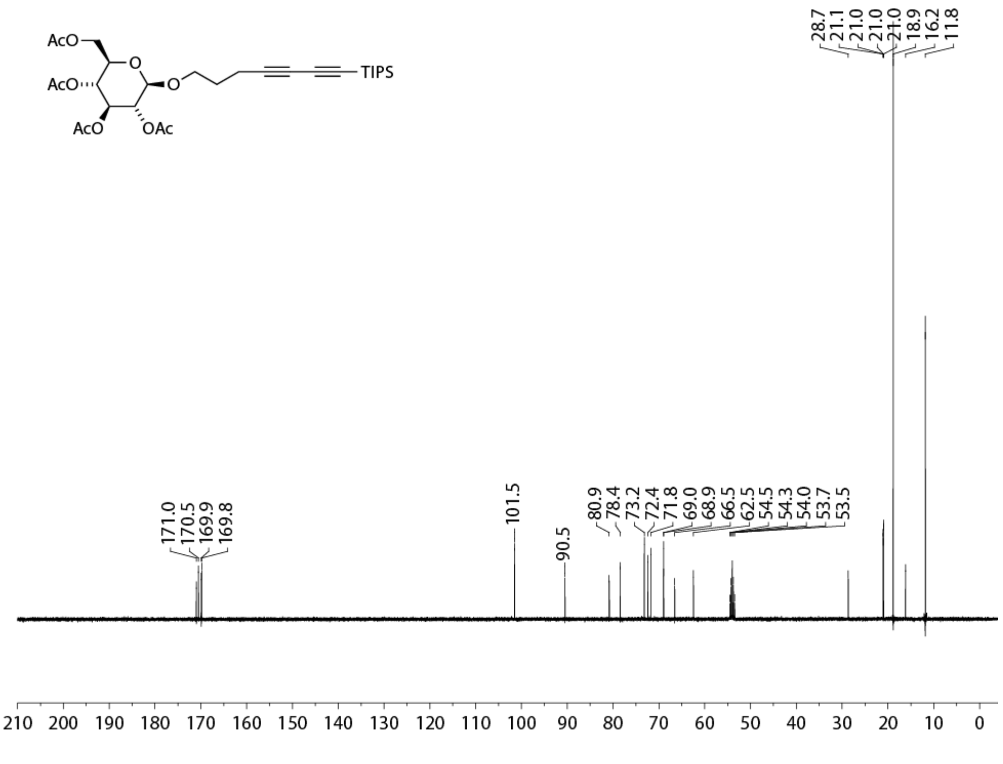

^{1}H NMR spectrum (CD_2Cl_2, 400 MHz) of **TAG3**

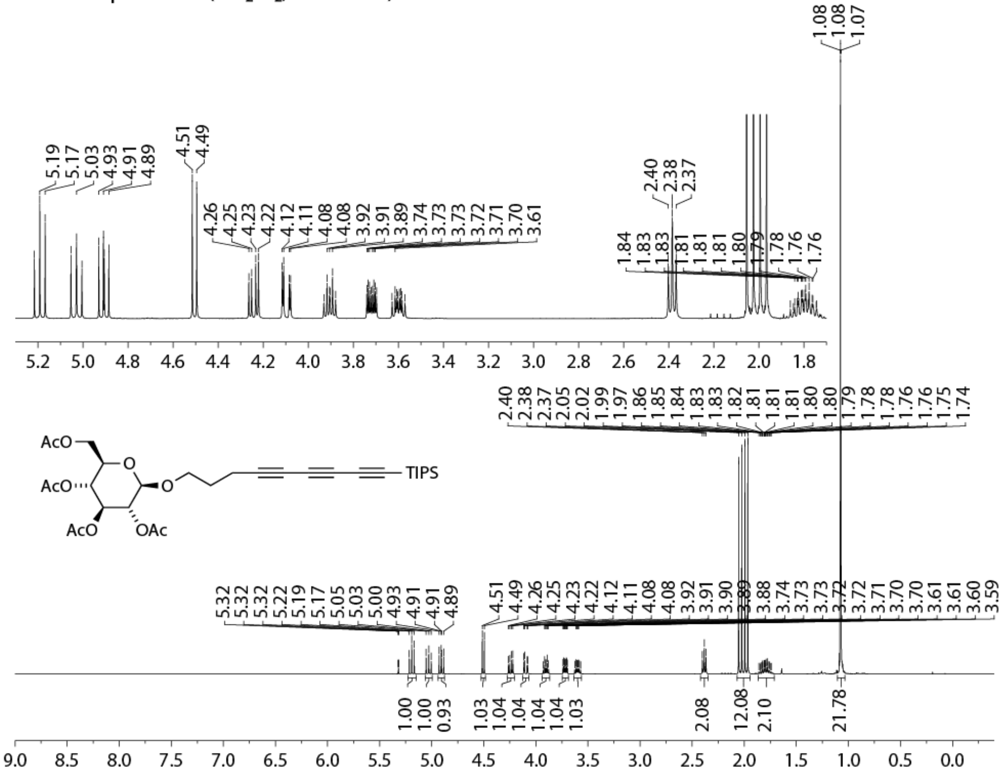

^{13}C NMR spectrum (CD_2Cl_2, 101 MHz) of **TAG3**

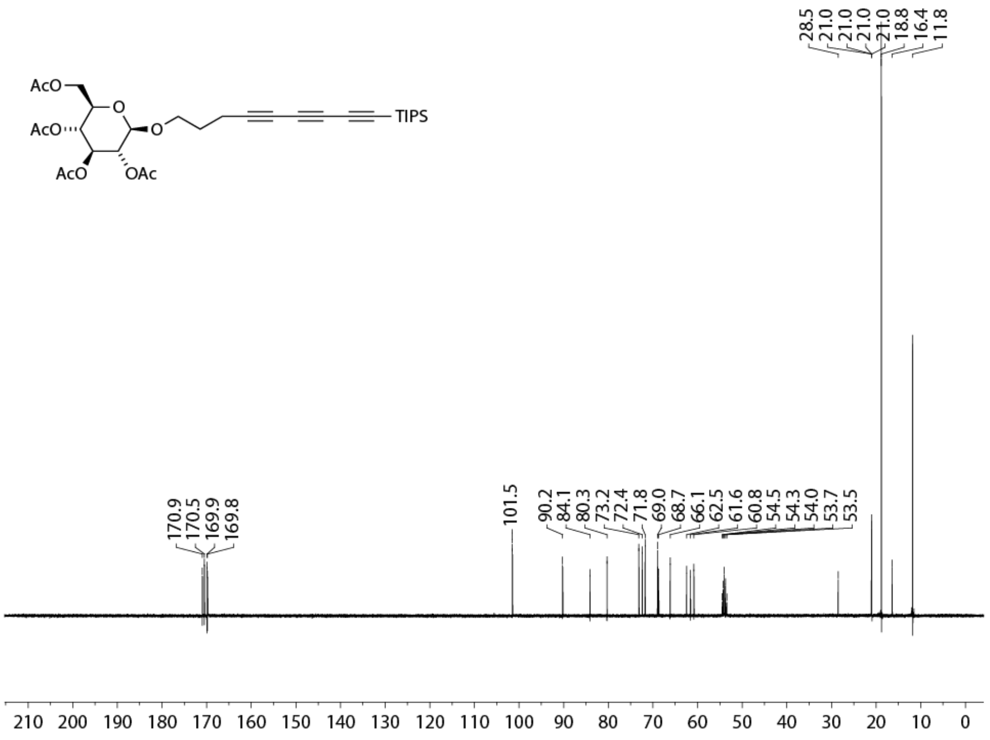

^{1}H NMR spectrum (CD_2Cl_2, 400 MHz) of **TAG4**

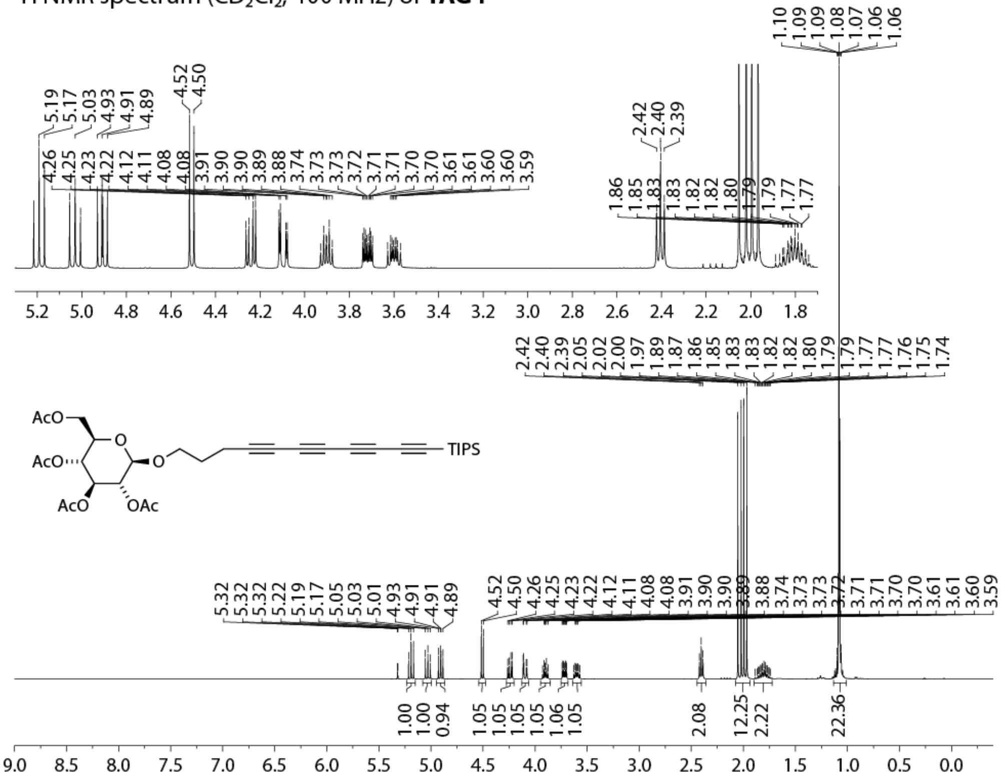

^{13}C NMR spectrum (CD_2Cl_2, 101 MHz) of **TAG4**

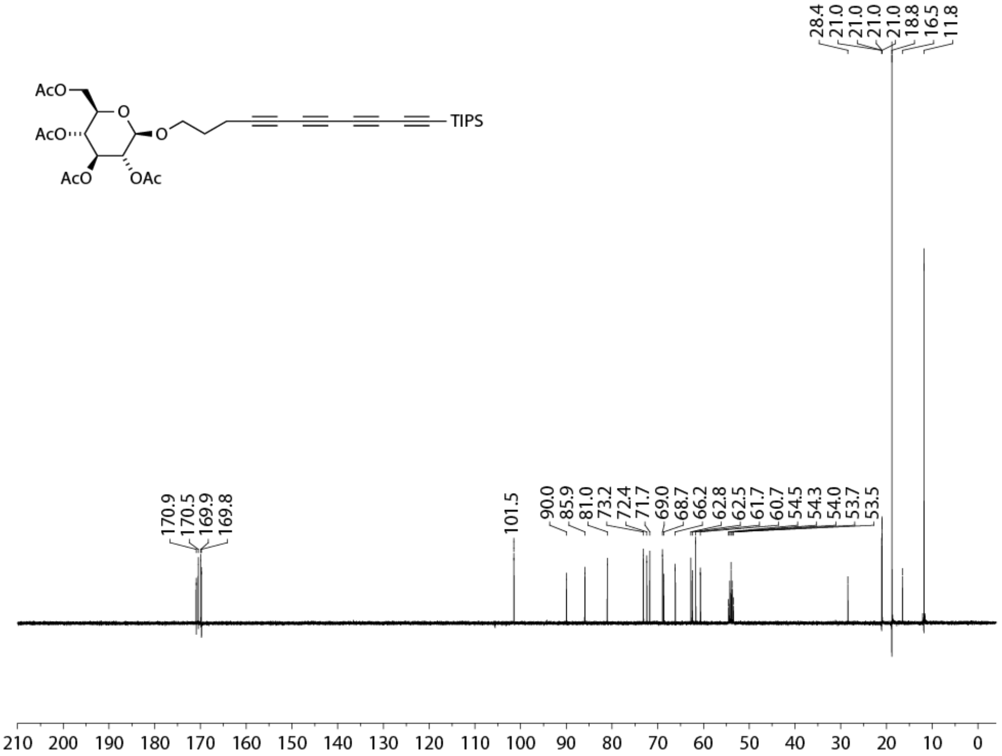

^{1}H NMR spectrum (CD_2Cl_2, 400 MHz) of **TAG5**

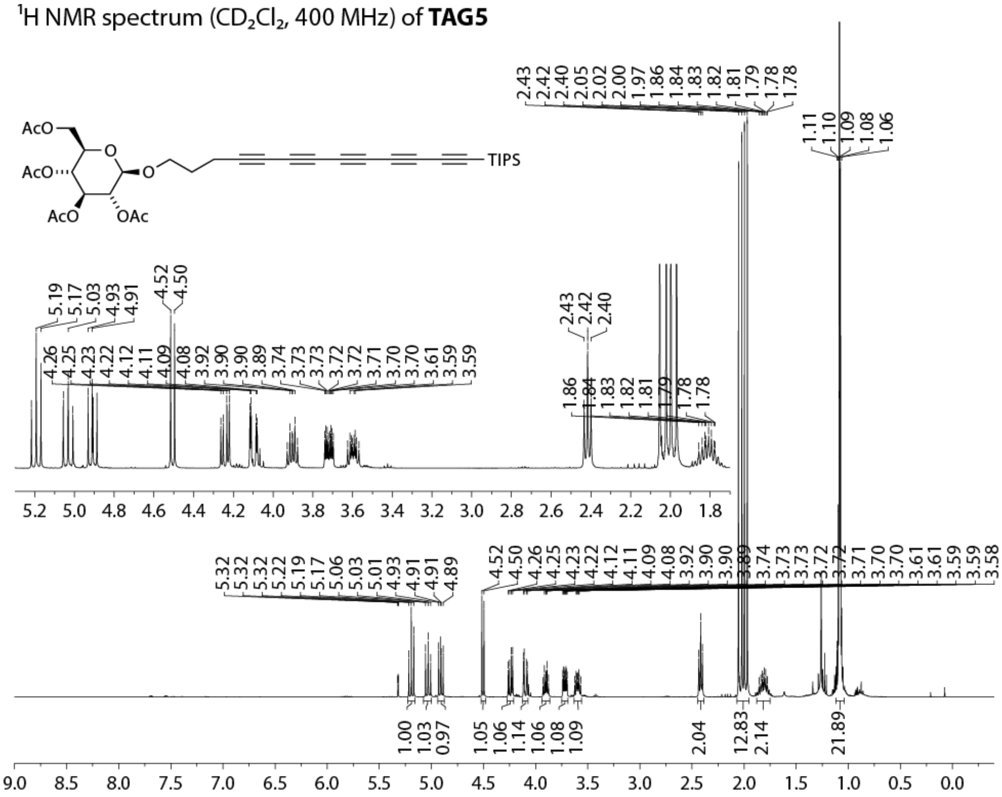

^{13}C NMR spectrum (CD_2Cl_2, 101 MHz) of **TAG5**

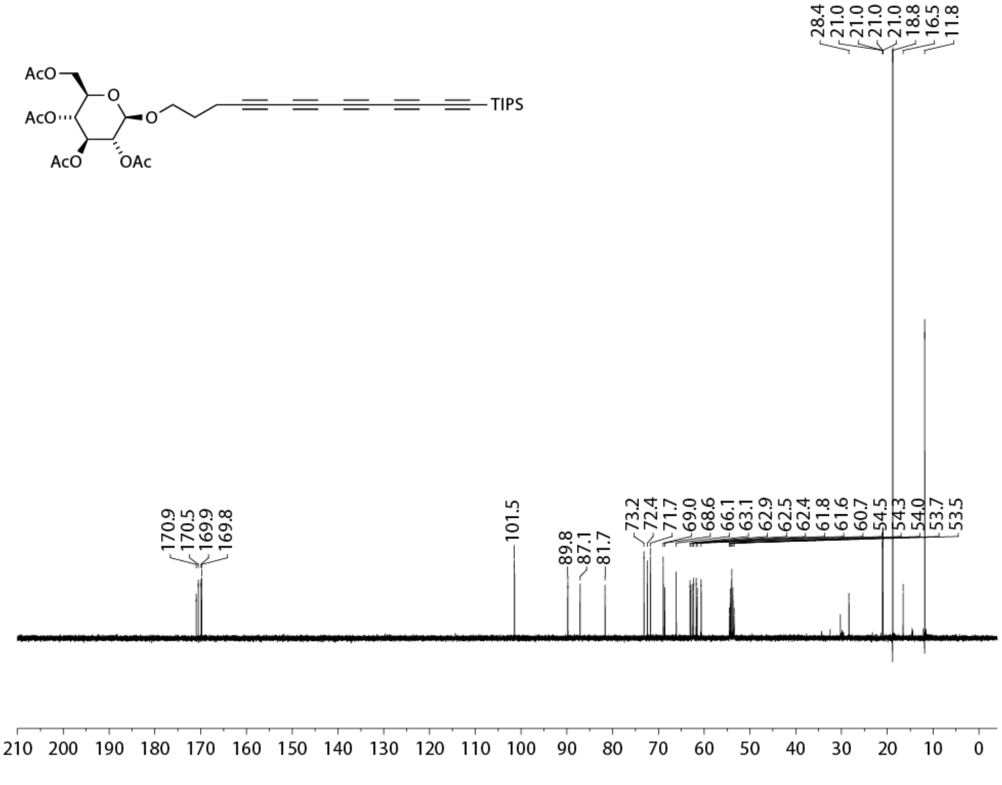

^{1}H NMR spectrum (CD_2Cl_2, 400 MHz) of **TAG6**

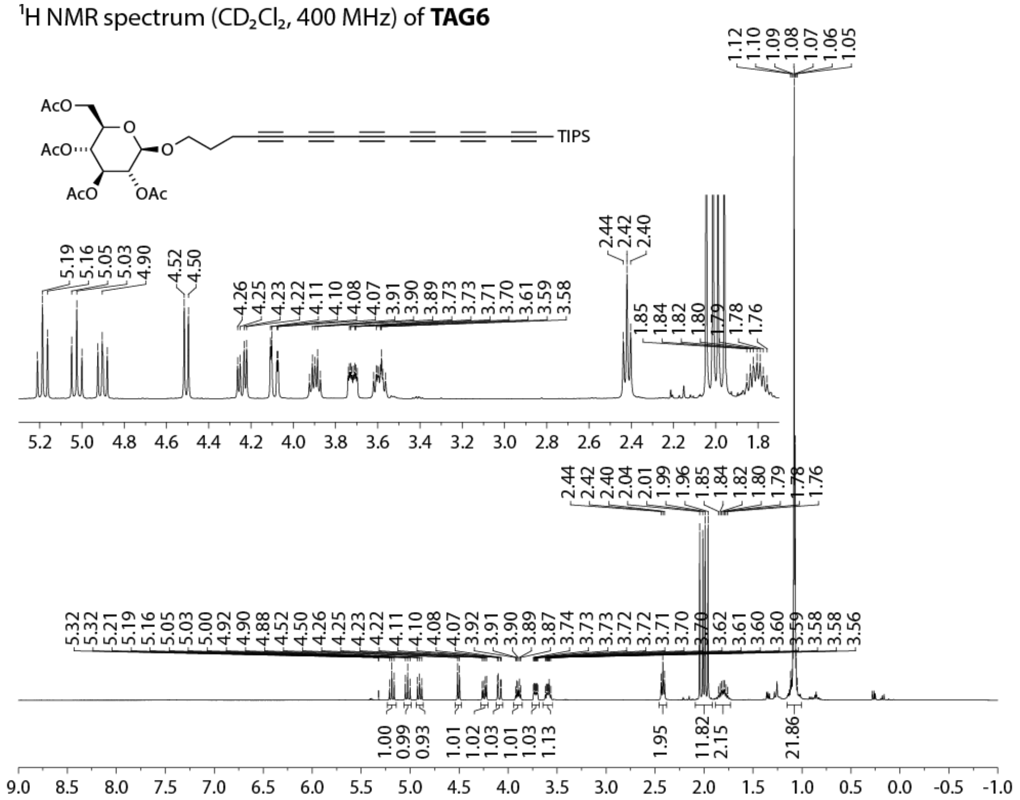

^{13}C NMR spectrum (CD_2Cl_2, 101 MHz) of **TAG6**

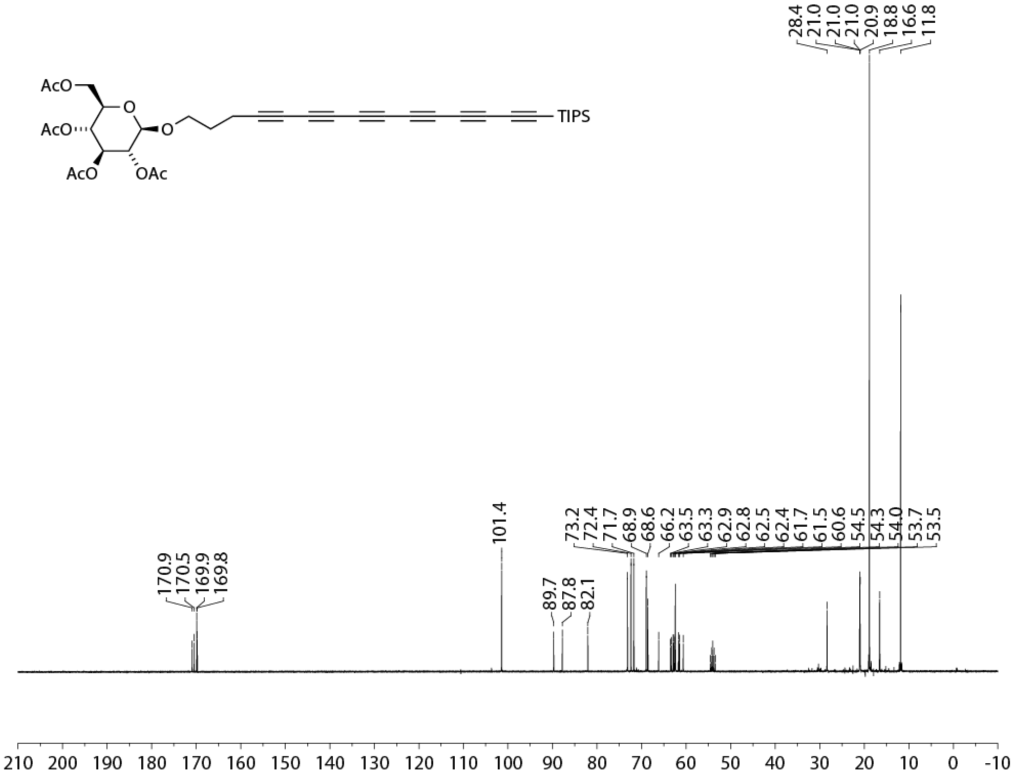

^{1}H NMR spectrum (CD_2Cl_2, 400 MHz) of **TPC2**

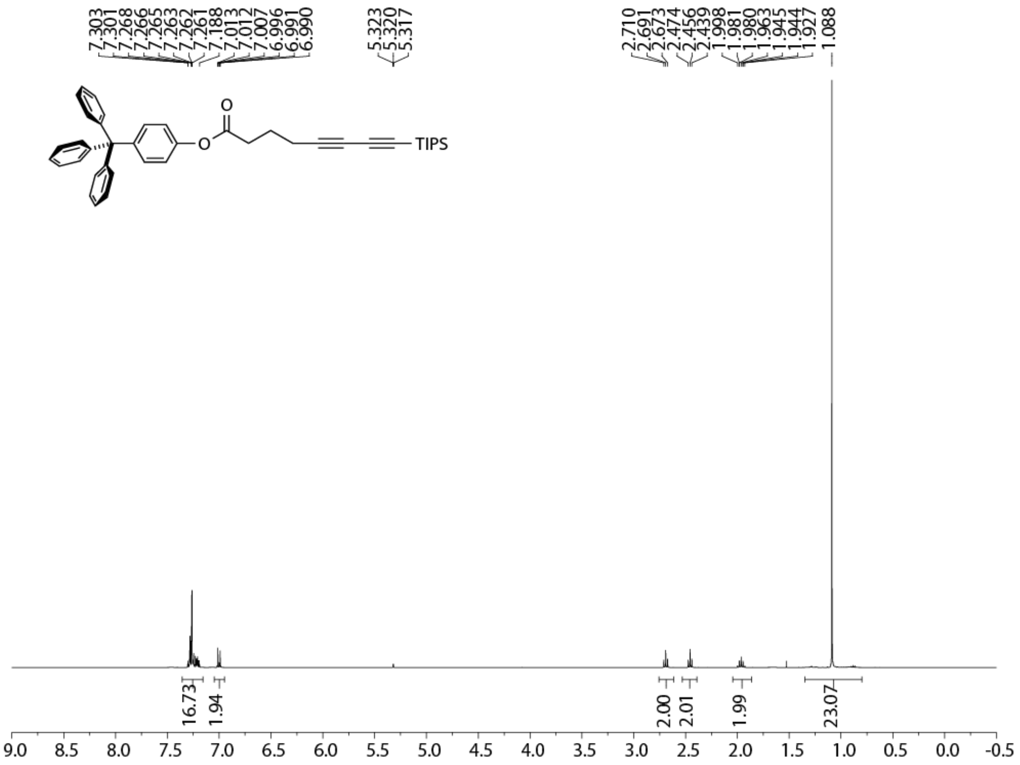

^{13}C NMR spectrum (CD_2Cl_2, 101 MHz) of **TPC2**

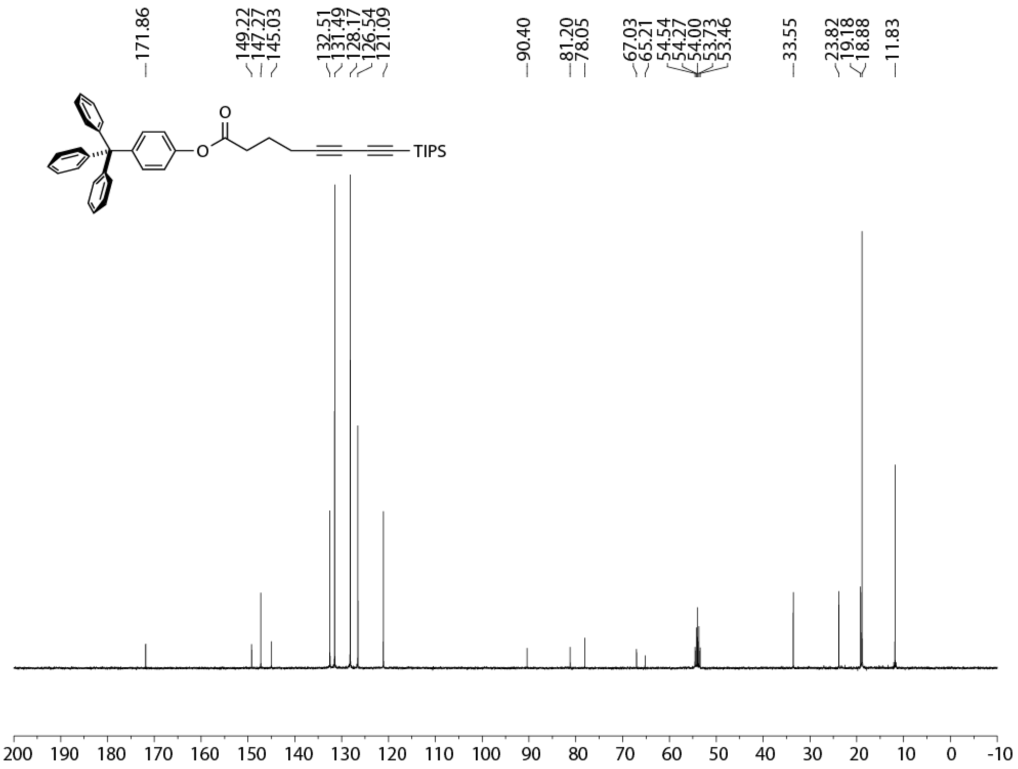

^{1}H NMR spectrum ($CDCl_3$, 400 MHz) of **TPC3**

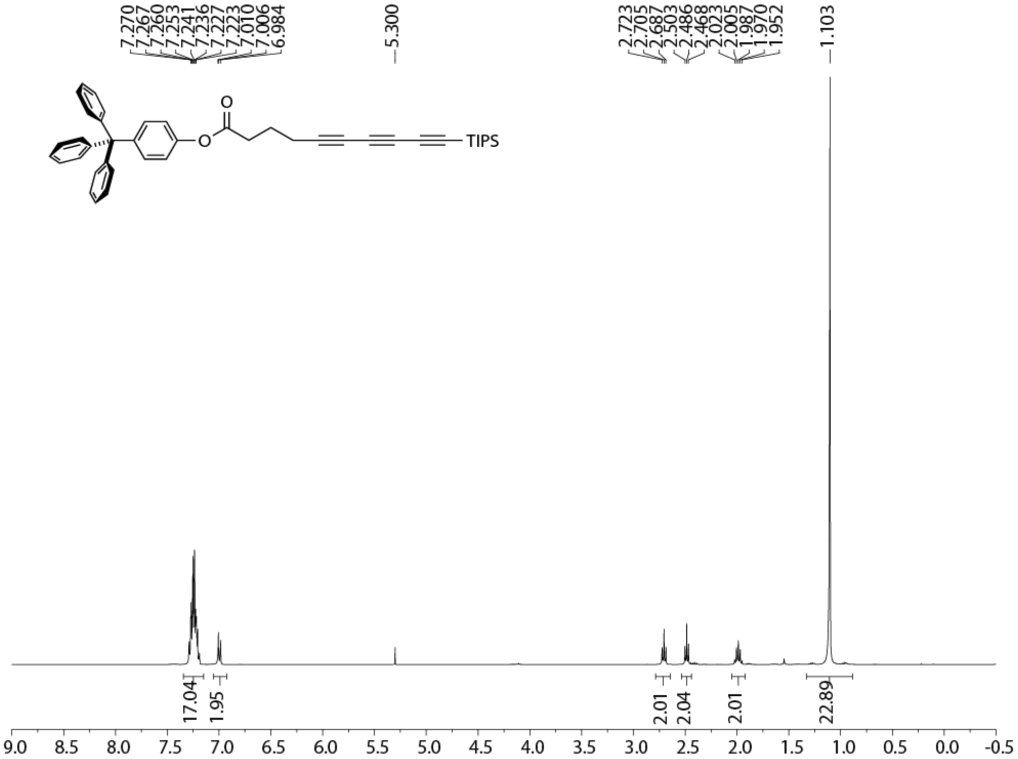

^{13}C NMR spectrum ($CDCl_3$, 101 MHz) of **TPC3**

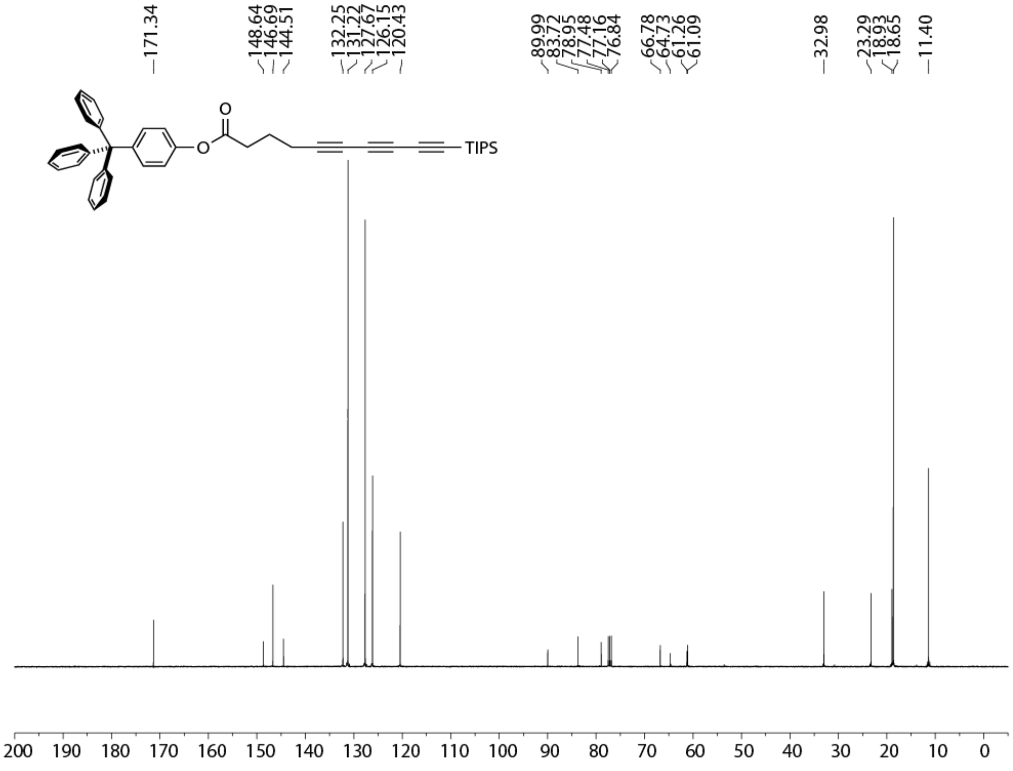

^{1}H NMR spectrum ($CDCl_3$, 400 MHz) of **TPC4**

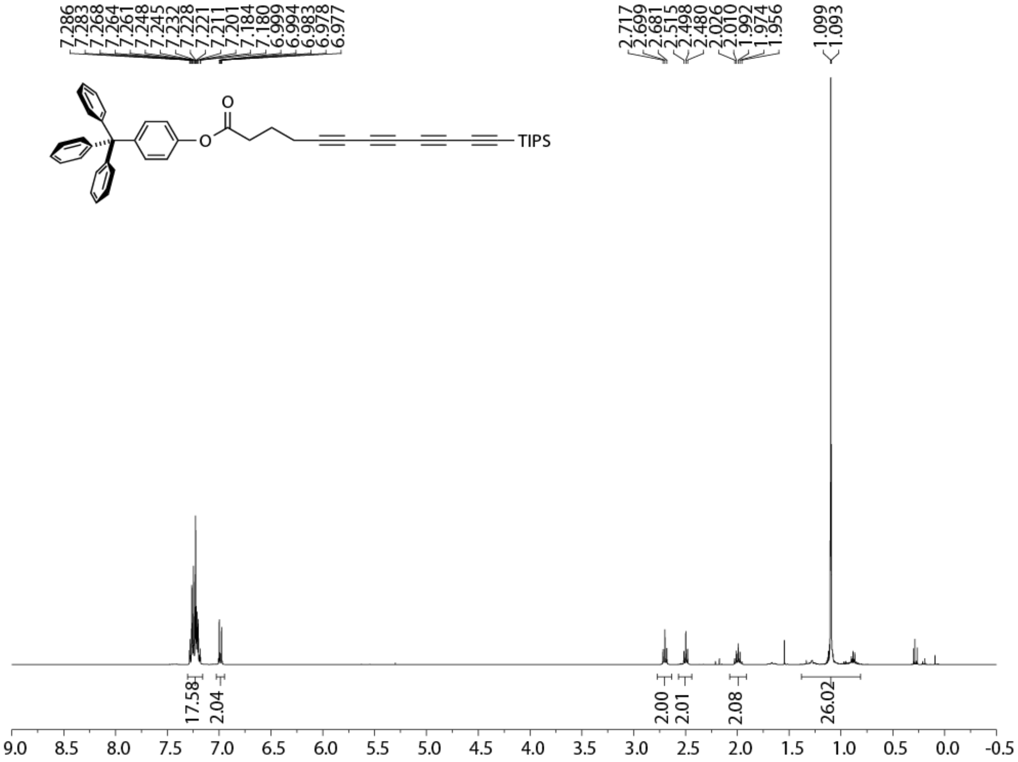

^{13}C NMR spectrum ($CDCl_3$, 101 MHz) of **TPC4**

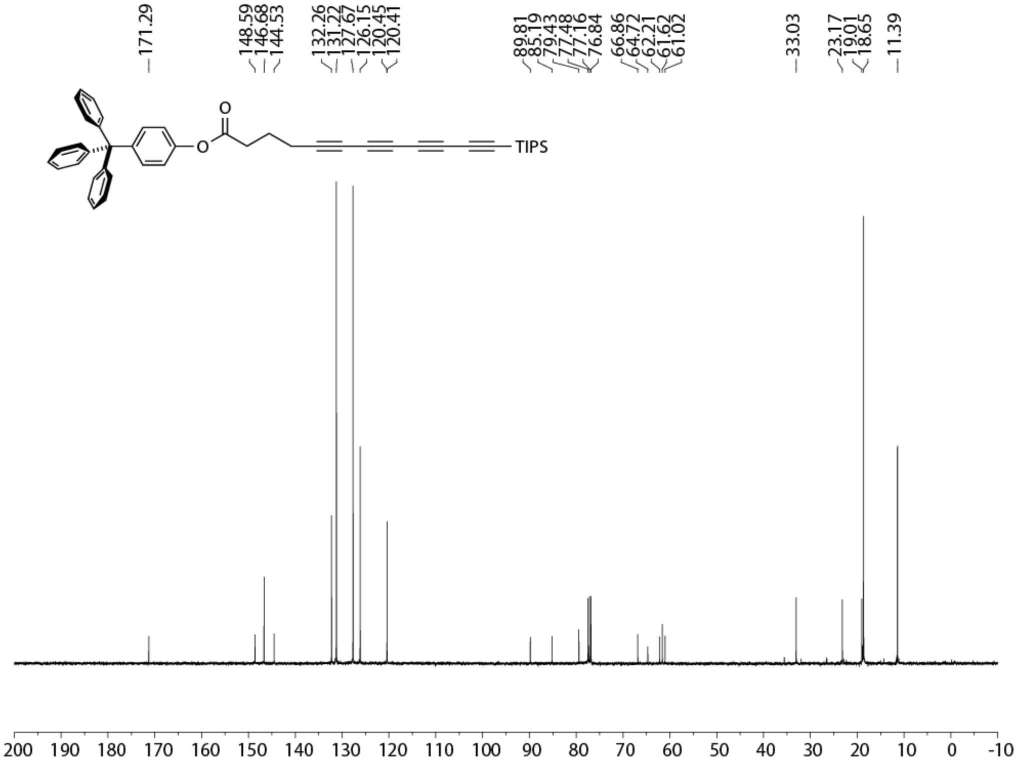

^{1}H NMR spectrum ($CDCl_3$, 400 MHz) of **TPC5**

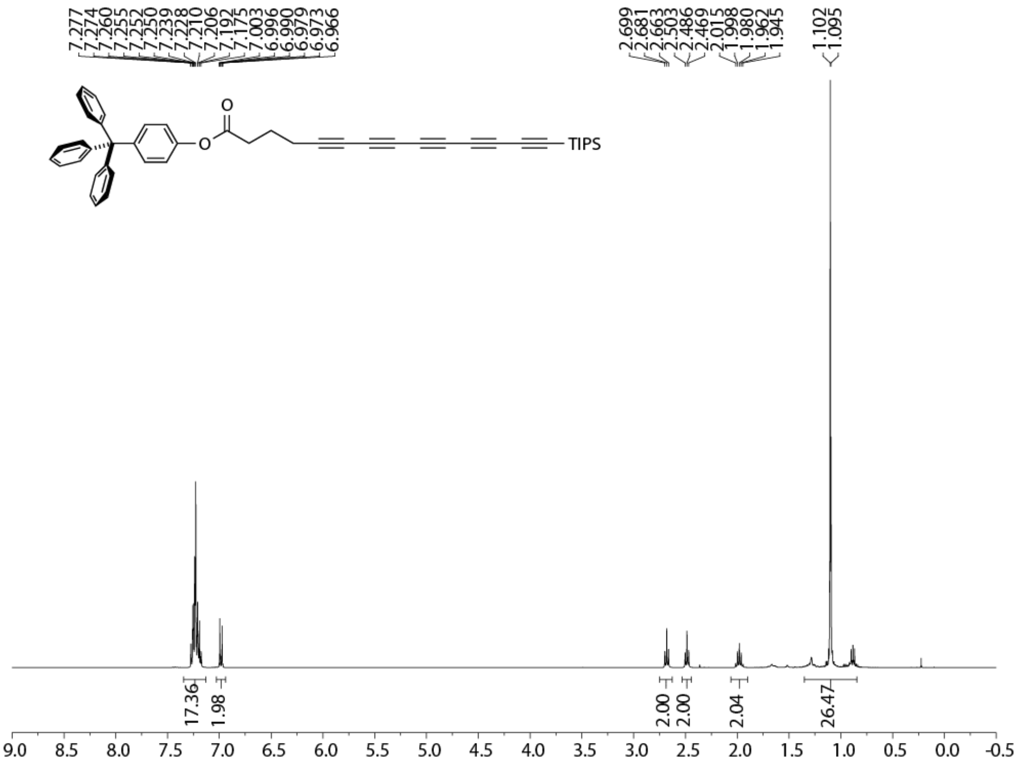

^{13}C NMR spectrum ($CDCl_3$, 101 MHz) of **TPC5**

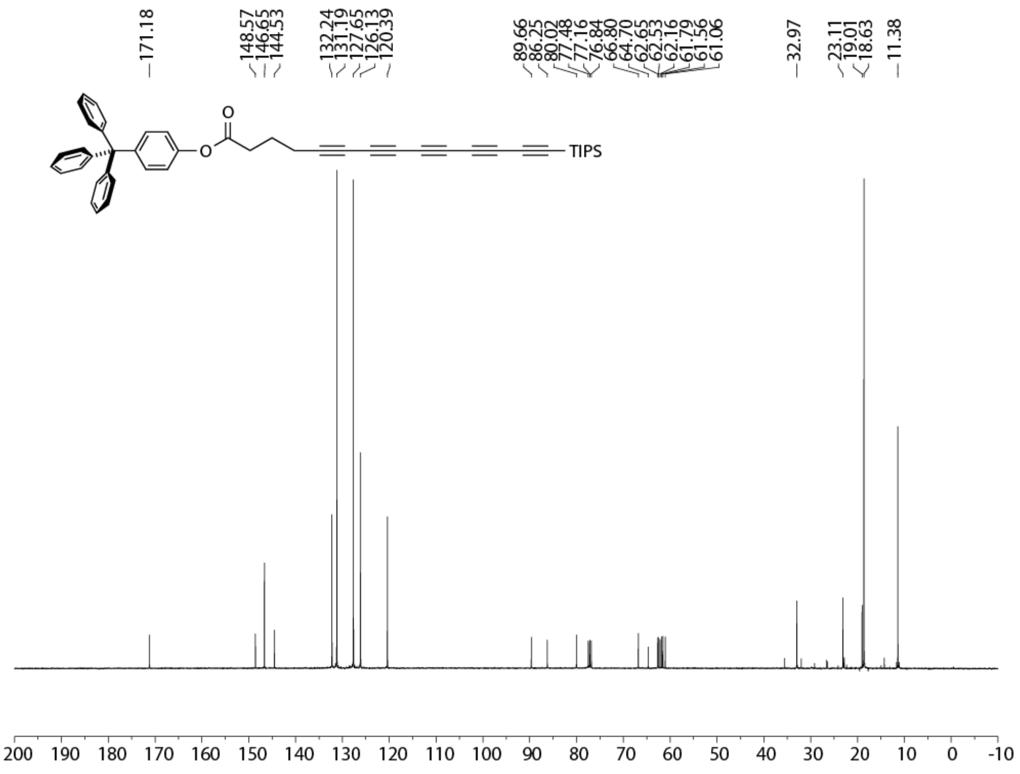

^1H NMR spectrum (CD_2Cl_2, 400 MHz) of **TPC6**

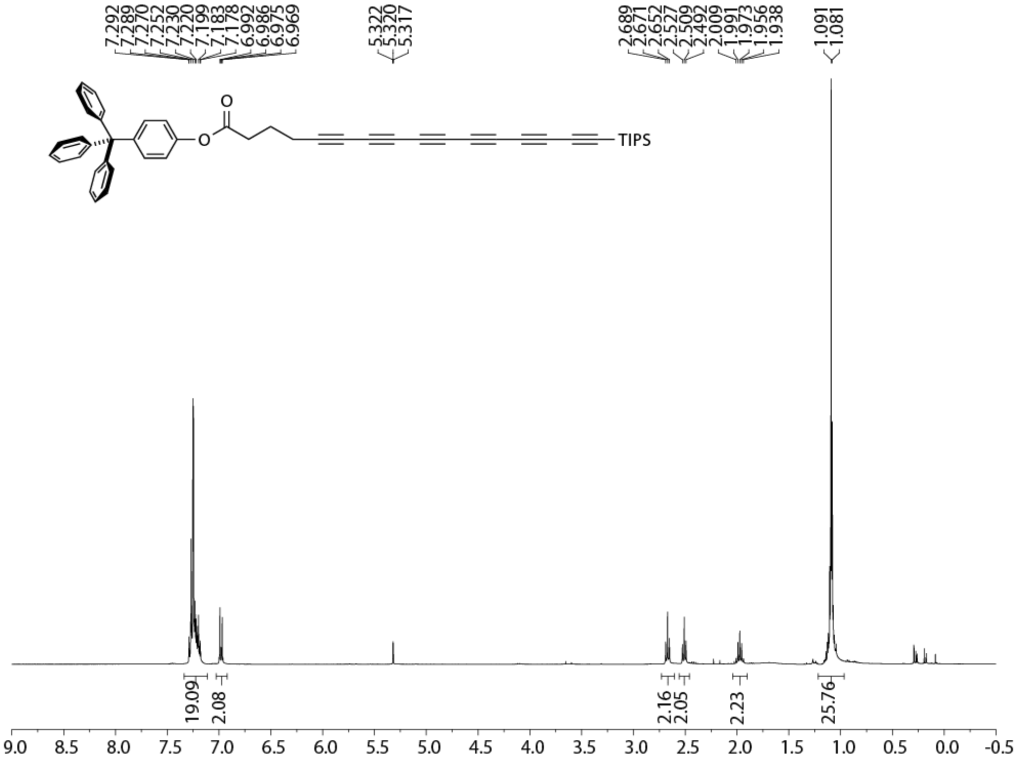

^{13}C NMR spectrum (CD_2Cl_2, 101 MHz) of **TPC6**

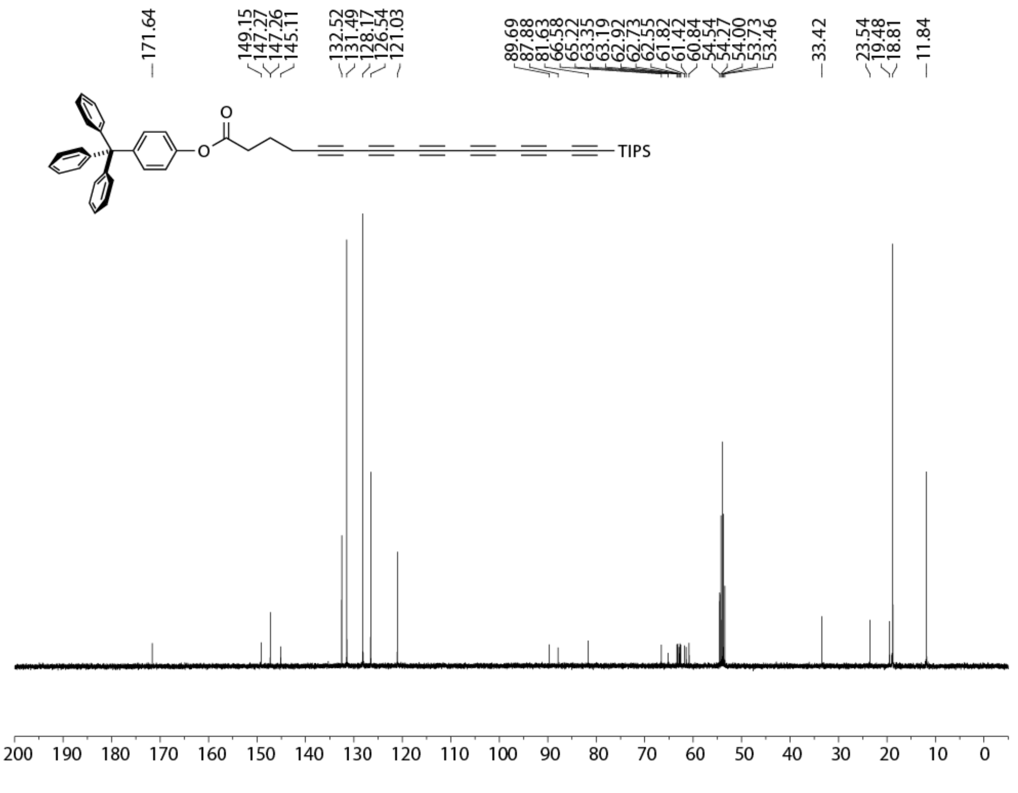

^{1}H NMR spectrum (DMSO-d$_6$, 400 MHz) of **GLU6-TIPS**

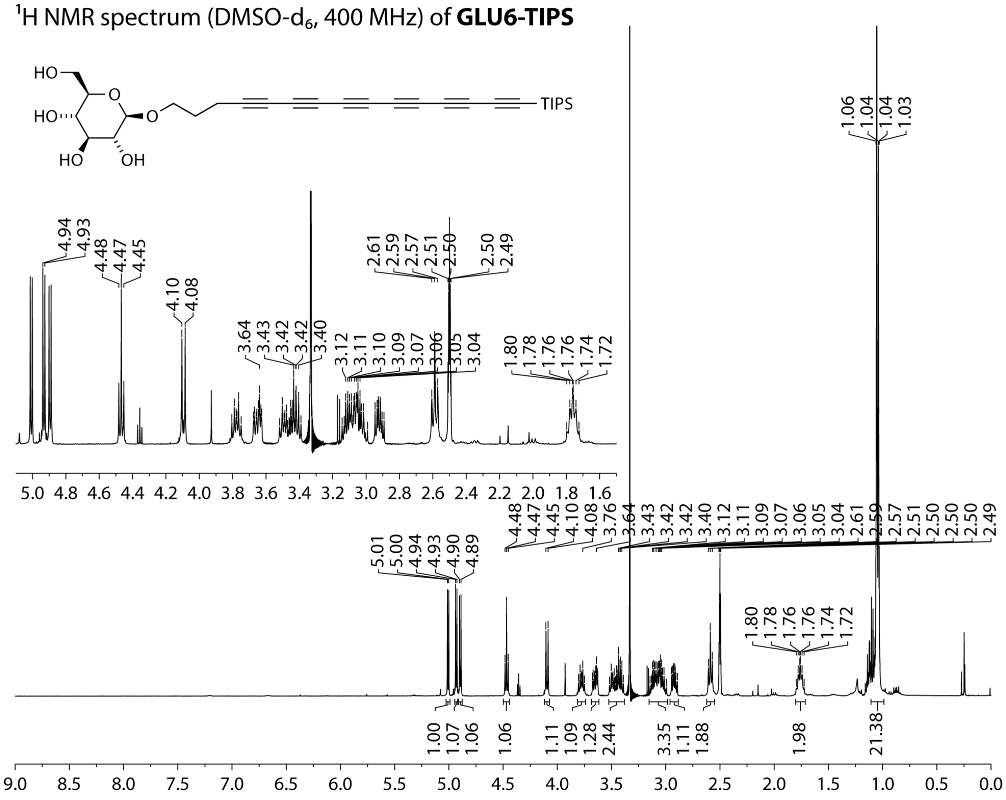

^{13}C NMR spectrum (DMSO-d$_6$, 101 MHz) of **GLU6-TIPS**

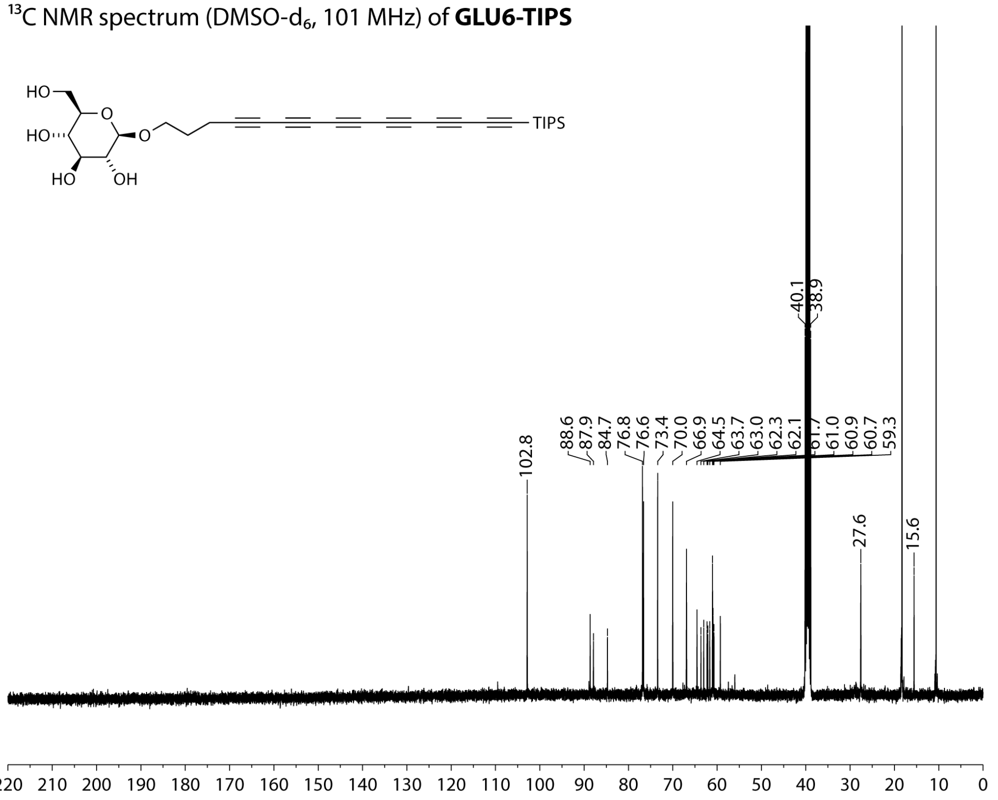

^{1}H NMR spectrum ($CDCl_3$, 400 MHz) of **MEC6-TIPS**

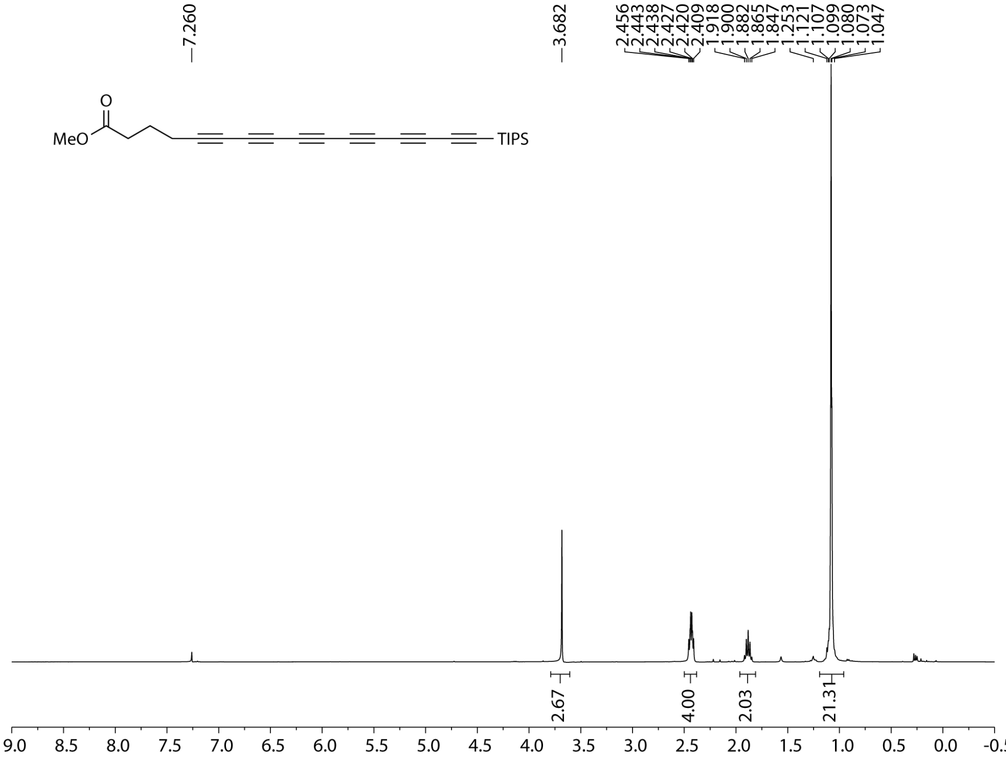

^{13}C NMR spectrum ($CDCl_3$, 101 MHz) of **MEC6-TIPS**

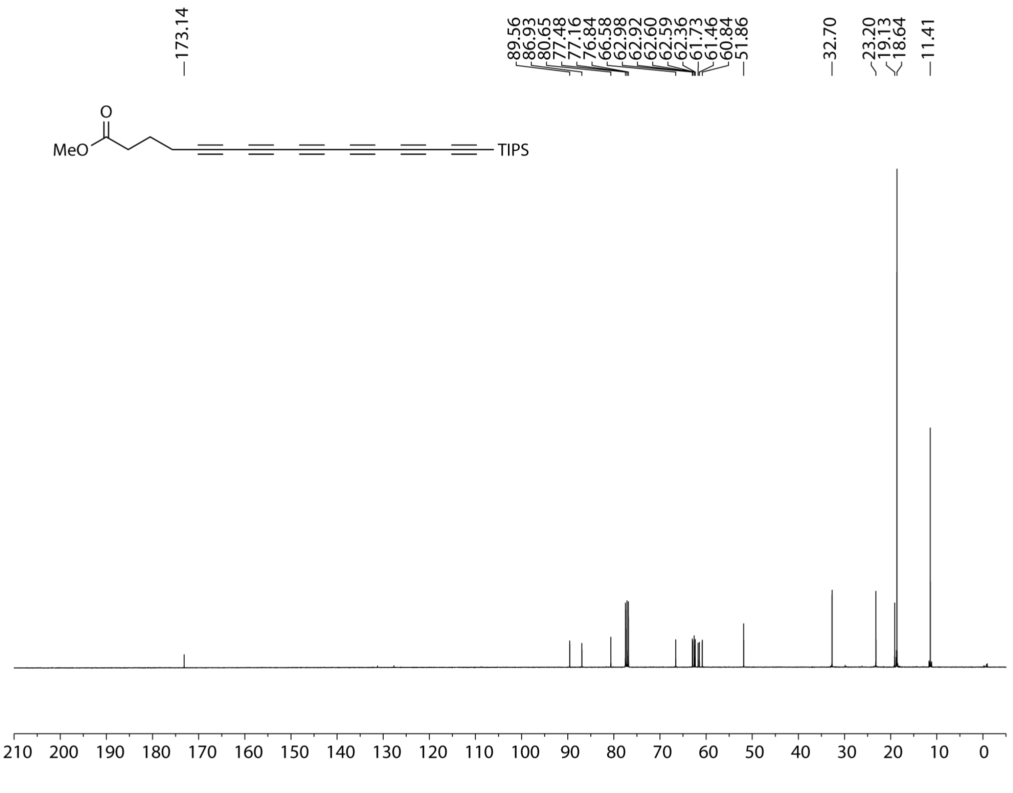

^{1}H NMR spectrum ($CDCl_3$, 400 MHz) of intermediate for **TAG6TAG**

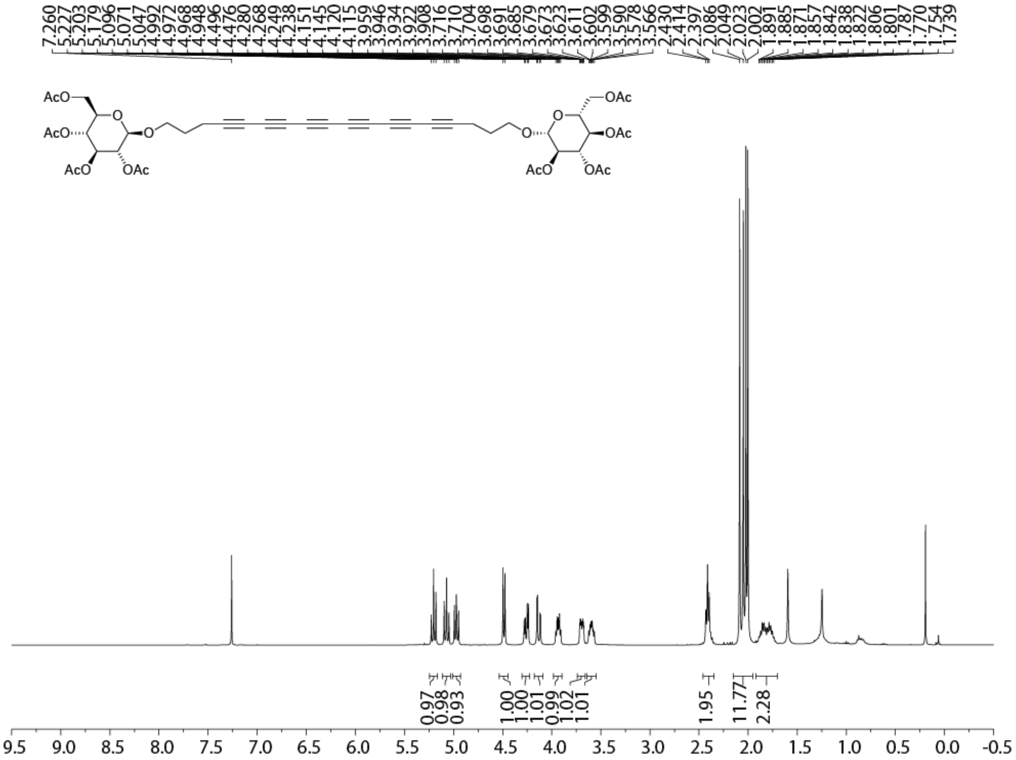

^{13}C NMR spectrum ($CDCl_3$, 101 MHz) of intermediate for **TAG6TAG**

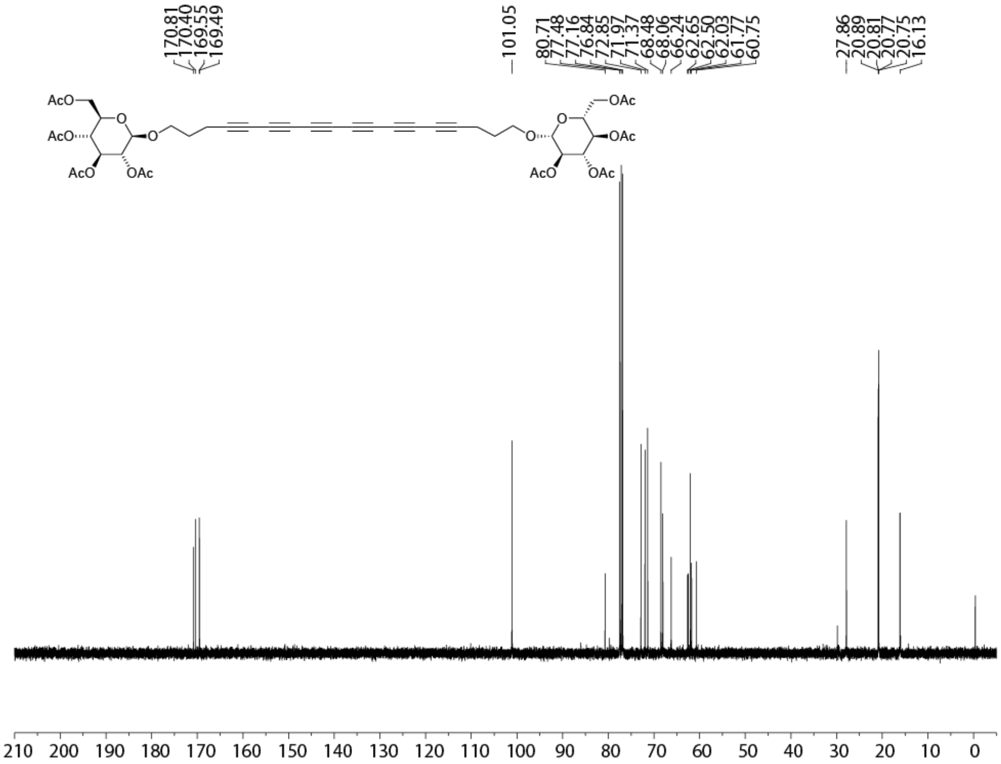

^{1}H NMR spectrum (MeOD, 400 MHz) of **GLU6GLU**

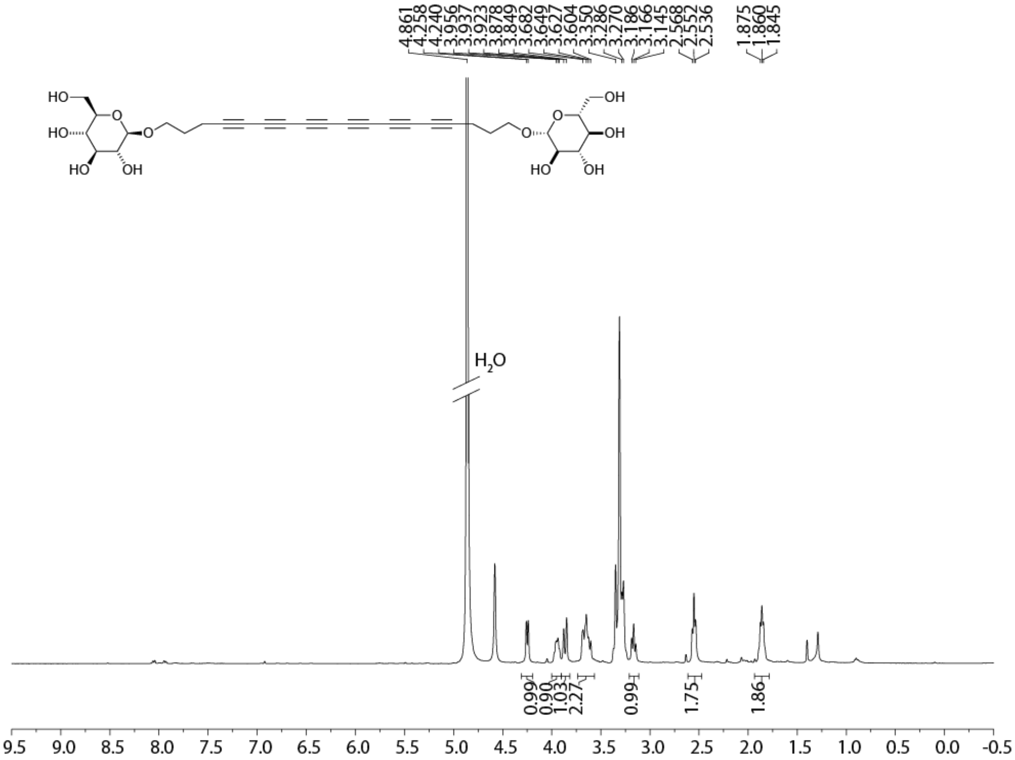

^{13}C NMR spectrum (MeOD, 101 MHz) of **GLU6GLU**

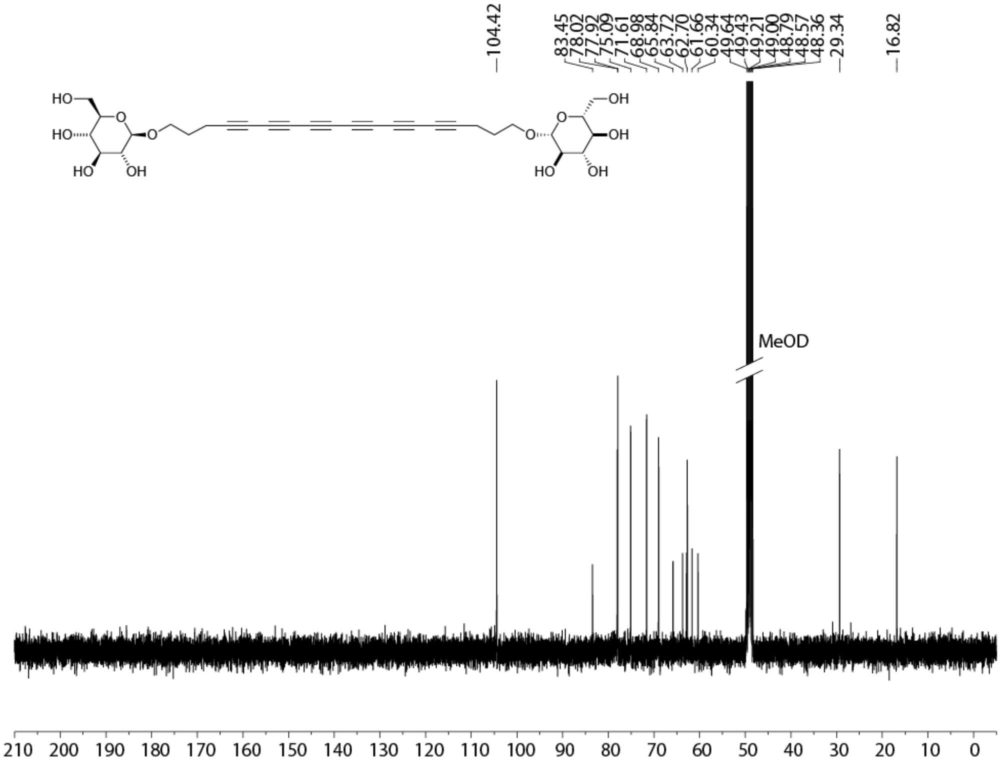

^{1}H NMR spectrum (D_2O, 400 MHz) of **GLU6GLU · 2 CD**

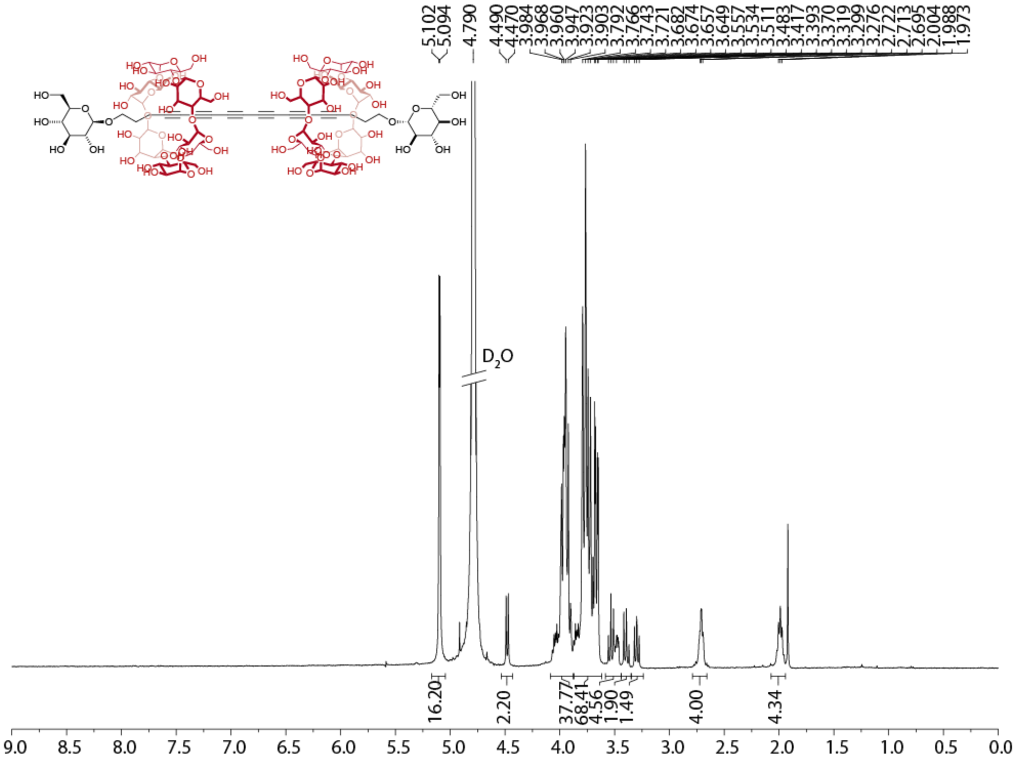

^{13}C NMR spectrum (D_2O, 101 MHz) of **GLU6GLU · 2 CD**

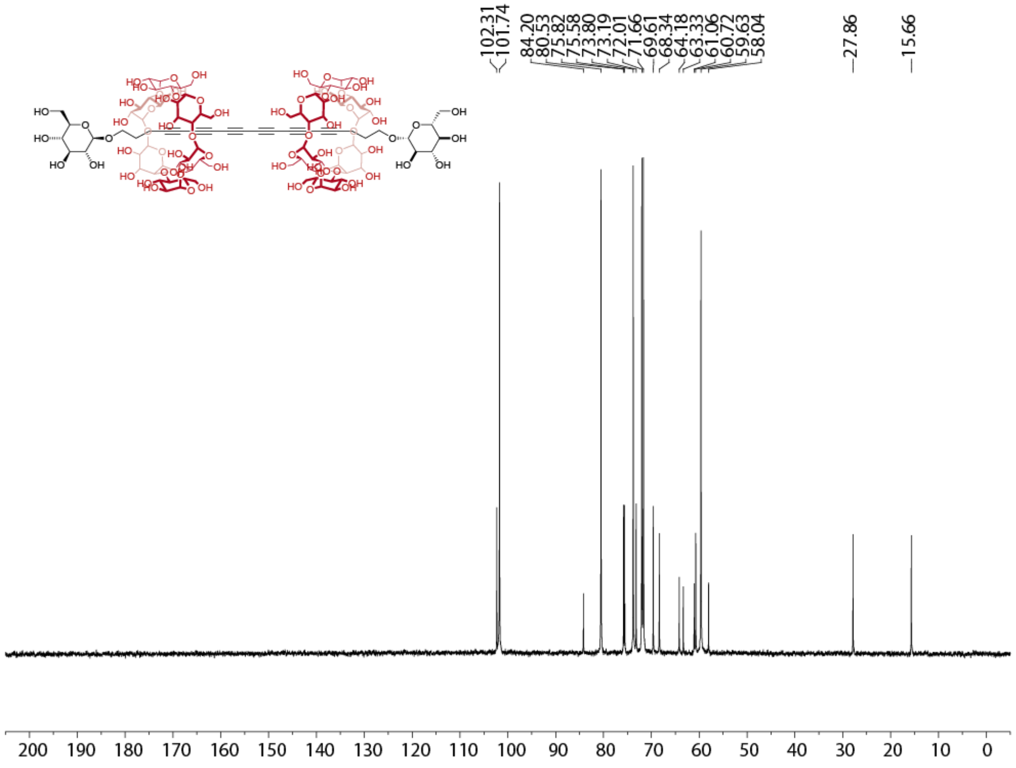

4. References

1. T. Luu, R. McDonald and R. R. Tykwinski, *Org. Lett.*, 2006, **8**, 6035-6038.
2. S. Ijadi-Maghsoodi and T. J. Barton, *Macromolecules*, 1990, **23**, 4485-4486.
3. D. H. Ballard and H. Gilman, *J. Organomet. Chem.*, 1968, **15**, 321-327.
4. S. Schmidt-Schaffer, L. Grubert, U. W. Grummt, K. Buck and W. Abraham, *Eur. J. Org. Chem.*, 2006, 378-398.
